# Polysulfamates as “Macroisosteres” of Polyurethanes with Improved Degradability

**DOI:** 10.1002/anie.202510841

**Published:** 2025-08-22

**Authors:** Srutashini Das, Katarzyna Doktor, Biswajit Saha, Felipe Cesar Sousa e Silva, Rachel M. Wynn, Quentin Michaudel

**Affiliations:** ^1^ Department of Chemistry Texas A&M University College Station Texas 77843 USA; ^2^ Department of Materials Science and Engineering Texas A&M University College Station Texas 77843 USA

**Keywords:** Degradable polymers, Macroisostere, Polysulfamates, Polyurethanes, SuFEx click chemistry

## Abstract

Addressing the environmental persistence of plastics requires the development of next‐generation polymers that combine high performance with enhanced degradability. Progress toward this grand challenge has been impeded, in part, by the absence of a general blueprint for the macromolecular design of such materials. Herein, we introduce a “macroisostere” design strategy, where the carbonyl group (–CO–) in polyurethanes (PUs) is replaced with a sulfonyl group (–SO_2_–), resulting in a virtually unknown family of polymers called polysulfamates. This approach, inspired by the use of bioisosteres in drug discovery, aims to preserve key interchain interactions that contribute to thermomechanical performance while enhancing the hydrolytic lability of the polymer backbone. The optimization of a Sulfur(VI) Fluoride Exchange (SuFEx) polymerization allowed the synthesis of ten polysulfamates structurally analogous to common PUs. Comparative analysis of one PU and its polysulfamate analog showed that this isosteric substitution increases thermal stability, slightly lowers the glass transition temperature, and retains similar hardness and reduced Young's modulus. Notably, the S(VI)‐based polysulfamate demonstrated significantly enhanced hydrolytic degradability. These results highlight the potential of the “macroisostere” approach as a generalizable strategy for designing high‐performance, degradable alternatives to traditional plastics.

## Graphical abstract

This study reports the synthesis of polysulfamates—sulfur(VI) isomeric analogs of polyurethanes in which the carbonyl group (–CO–) in the repeating unit is replaced by a sulfonyl group (–SO_2_–). Analysis of these polyurethane “macroisosteres” revealed enhanced hydrolytic degradability, improved thermal stability, and retained mechanical properties.

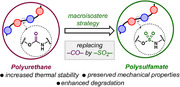

The make–use–dispose paradigm prevalent in plastic consumption has created a global environmental crisis, prompting the call for the development of sustainable alternatives with embedded degradability for chemical recycling or upcycling.^[^ ^1^, ^2^, ^3^ ^]^ However, the vastness of chemical space makes the identification of next‐generation polymers challenging in the absence of guiding design principles, as it remains difficult to predict which structural motifs in a polymer backbone will simultaneously provide desirable thermomechanical properties and enhanced degradability.^[^ ^4^, ^5^ ^]^ While the optimization of machine learning models will help accelerate the development of sustainable polymers, targeted complementary synthetic efforts remain essential to inform and guide their predictions.^[^ ^6^, ^7^ ^]^ Drawing inspiration from drug development—where bioisosteres, structural replacements designed to maintain the function of a pharmaceutical ingredient while enhancing key physicochemical properties, are widely used^[^ ^8^ ^]^—we hypothesized that isosteric replacement could lead to polymers with enhanced degradability from high‐commodity building blocks. As a case study, we report the synthesis of polysulfamates, an unprecedented class of polymers analogous to polyurethanes (PUs) through isosteric modification of the carbonyl (–CO–) functional group by a sulfonyl (–SO_2_–) motif. PU is one of the most versatile classes of synthetic polymers and is composed of repeating carbamate units arising from the condensation of diisocyanates with diols or polyols.^[^ ^9^ ^]^ PU ranks as the sixth most produced polymer, with a global market volume of ∼56 billion pounds (∼$87 billion market value) in 2023 and a projected compound annual growth rate of 4.2%.^[^ ^10^, ^11^ ^]^ Its tunable properties and modular synthesis has generalized its uses in coatings, adhesives, sealants, and elastomer industries, as well as in foams for construction and comfort.^[^ ^9^, ^10^, ^11^, ^12^ ^]^ However, conventional disposal strategies are incineration and landfilling.^[^ ^13^, ^14^ ^]^ Mechanical recycling applied to PU waste typically provides materials with lower values,^[^ ^15^ ^]^ which has recently prompted the development of catalytic methods to reprocess postconsumer PU foams with exchange chemistry.^[^ ^16^, ^17^ ^]^ Alternatively, chemical recycling approaches have been investigated to depolymerize PU and recover its precursors.^[^ ^18^, ^19^ ^]^ For instance, Dow's RENUVA™^[^ ^20^ ^]^ and Covestro's Evocycle® CQ^[^ ^21^ ^]^ programs are promising initiatives, although they are currently limited to linear, flexible PU, and in the case of RENUVA, focus solely on the recovery of the polyol component. Most reported deconstruction conditions for PUs require high temperature (>150 °C) and pressure (20–80 atm),^[^ ^14^, ^22^, ^23^ ^]^ or expensive transition metal catalysts,^[^ ^24^, ^25^, ^26^, ^27^ ^]^ which likely preclude large‐scale implementation. Additionally, hydrolysis under chemical or biological conditions releases carbon dioxide, a greenhouse gas.^[^ ^14^, ^23^, ^28^ ^]^

We envisioned that a –SO_2_– for –CO– isosteric modification of the PU backbone would maintain the desirable physical properties of PUs while enhancing their degradability. The unique behavior of thermoplastic PU elastomers, for example, arises from the presence of soft and hard segments—the latter aggregating into crystalline microdomains through hydrogen bonding.^[^ ^29^, ^30^ ^]^ The targeted substitution leading to a sulfamate ester motif in the repeating unit would preserve a polar motif including hydrogen‐bond donors and acceptors,^[^ ^31^, ^32^, ^33^ ^]^ while introducing a group more susceptible to hydrolysis (Figure 1). Computational studies by Chataigner et al. suggest a decreased donation of the nitrogen lone pair to the sulfonyl group compared to the carbonyl, which should increase the electrophilicity of the S(VI) group, making it more susceptible to water or hydroxide attack.^[^ ^34^ ^]^ Additionally, the increased lability of –SO_2_– has been experimentally demonstrated through a comparative hydrolytic study of diphenylsulfamide and the isosteric diphenylurea,^[^ ^35^ ^]^ as well as in macromolecular settings with the recent synthesis of degradable polysulfamides.^[^ ^36^, ^37^ ^]^ Importantly, the presence of a monosubstituted sulfamate ester motif (RNHSO_2_OR′) is crucial for efficient hydrolysis, in contrast to the hydrolytically stable disubstituted variant (R_2_NSO_2_OR′).^[^ ^38^ ^]^ While polysulfamates are virtually unknown,^[^ ^39^ ^]^ the recent advent of Sulfur(VI) Fluoride Exchange click chemistry (SuFEx)^[^ ^40^, ^41^, ^42^, ^43^, ^44^, ^45^ ^]^ has opened the door to the synthesis of S(VI)‐containing polymers.^[^ ^40^, ^41^, ^46^, ^47^, ^48^, ^49^, ^50^, ^51^, ^52^, ^53^ ^]^ Herein, we report the development of a SuFEx polymerization combining bis(sulfamoyl fluoride) and bis(silyl ether) monomers to access a variety of monosubstituted polysulfamates as “macroisosteres” of diverse PUs. This approach enabled the successful development of structurally diverse polysulfamates with high thermal stability, tunable glass transition temperatures through modulation of the polymer backbone, and improved degradability profiles compared to PUs. Importantly, the reported SuFEx polymerization leverages building blocks that are already produced on an industrial scale for the manufacturing of PUs.

**Figure 1:**
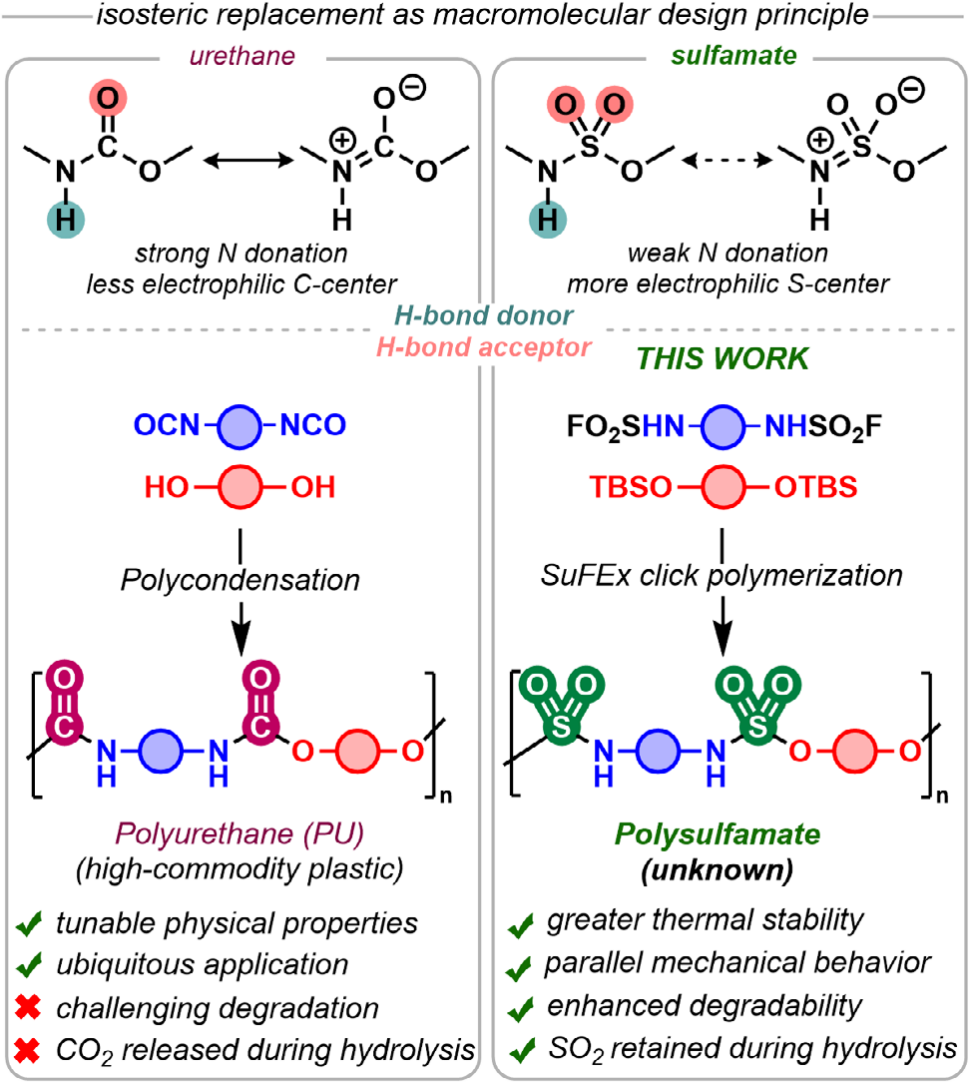
Polysulfamates as “macroisosteres” for PUs: design hypothesis and SuFEx polymerization.

Although SuFEx polymerizations have been successfully developed for a variety of S(VI)‐based polymers including polysulfates,^[^ ^47^, ^49^, ^53^, ^54^, ^55^, ^56^ ^]^ polysulfonates,^[^ ^56^, ^57^ ^]^ and polysulfamides,^[^ ^36^, ^37^, ^58^ ^]^ polysulfamates present a unique challenge due to the presence of two different heteroatoms (O and N) that require selective incorporation through suitable SuFEx nucleophiles. Two strategies can be envisioned (see Table 1): (1) Formation of the S(VI)–OR bond via a sulfamoyl fluoride and an alcohol or (2) Formation of the S(VI)–NHR bond using a fluorosulfate ester and an amine. Importantly, the S(VI)–OR bond is known to be labile, particularly with phenol derivatives (R = Ar), which has been leveraged in the click process Sulfur(VI) Phenolate Exchange (SuPhenEx).^[^ ^59^, ^60^ ^]^ To mitigate the risk of backbone scrambling through an undesired combination of SuFEx and SuPhenEx, we opted to start our synthetic exploration with the former route involving the combination of a diol and bench‐stable bis(monosubstituted sulfamoyl fluoride). This monomer can be synthesized on multi‐gram scale in one step from commercially available diamines and SuFEx‐IT, a crystalline solid source of “SO_2_F^+^”.^[^ ^36^, ^40^, ^61^ ^]^ Monosubstituted sulfamoyl fluoride derivatives are known to generate highly electrophilic azasulfene intermediates in the presence of a base,^[^ ^36^, ^62^, ^63^ ^]^ which can be efficiently trapped by amine or alcohol nucleophiles without the need of stoichiometric activators (e.g., calcium triflimide^[^ ^64^ ^]^ or hydroxybenzotriazole^[^ ^65^ ^]^) typically used with fluorosulfates (ROSO_2_F). This approach was thus anticipated to minimize the production of byproducts, potentially leading to tedious purifications. Combination of **1a** and bisphenol A (**2**) with DBU (0.5 equiv) in MeCN led to the formation of oligomers (Strategy 1, Table 1, Entry 1). Screening of reaction parameters did not drastically improve the obtained molar masses (Table S1). Silylated alcohols have been showed to facilitate SuFEx chemistry with sulfonyl fluorides,^[^ ^41^ ^]^ putatively through a concerted nucleophilic addition and fluoride elimination pathway.^[^ ^66^ ^]^ Another advantage of using silyl ethers as nucleophiles is their ability to sequester fluoride ions as R_3_SiF during the SuFEx reaction.^[^ ^67^ ^]^ Although silyl ethers have not been previously reported in combination with sulfamoyl fluorides, this prior work motivated us to synthesize bis(silyl‐ether) monomer **3a**. While the molar mass of **P1** remained low when using **1a** and **3a** in MeCN, a stark enhancement was observed in DMF (*M*_w_ = 13.7 kg mol^−1^) and DCM (*M*_w_ = 19.3 kg mol^−1^) (Table 1, Entries 2–4). Increasing the temperature to 35 °C led to a *M*_w_ of 36.6 kg mol^−1^ (Table 1, Entry 5). Other bases including pyridine and triethylamine did not perform as well as DBU (Table 1, Entries 6, 7 and Table S1). To evaluate our initial hypothesis regarding the polysulfamate synthesis strategy, we applied the optimized conditions to the reaction between bis(fluorosulfonate) **5** and diamine **4** (Strategy 2, Table 1, Entries 8, 9). Although small oligomers of **P1** were obtained, this second set of monomers led to the formation of a variety of contaminating side‐products alongside **P1**.

**Table 1:** Optimization for polysulfamate sythesis.

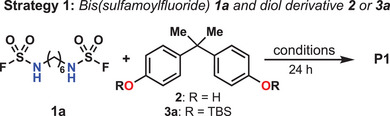						
Entry	R	Solvent	Base^a)^	Temp. (°C)	*M*_w_ ^b)^ (kg mol^−1^)	*Ɖ*
1	H	MeCN	DBU	rt	2.0	2.08
2	TBS	MeCN	DBU	rt	3.8	2.55
3	TBS	DMF	DBU	rt	13.7	1.74
4	TBS	DCM	DBU	rt	19.3	1.87
5	TBS	DCM	DBU	35	36.6	2.35
6	TBS	DCM	Pyridine	35	–	–
7	TBS	DCM	Et_3_N	35	9.4	1.79

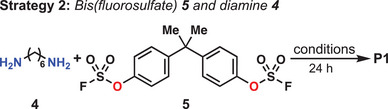						
Entry	R	Solvent	Base^a)^	Temp. (°C)	*M*_w_ ^b)^ (kg mol^−1^)	*Ɖ*
8	–	DCM	DBU	35	6.6	4.37
9	–	DMF	DBU	50	3.0	1.75

^a)^ 0.5 equiv of base.

^b)^ *M*_w_’s and *Đ*’s were determined by SEC (DMF + 0.01% LiBr) using poly(methyl methacrylate) standards.

To demonstrate the modularity of the SuFEx polymerization, a variety of bis(sulfamoyl fluoride)s **1a–1d** and silylated diols **3a–3d** (*see Supporting Information*) were prepared and copolymerized (Figure 2). Aliphatic bis(sulfamoyl fluoride)s **1a** and **1c** reacted with aromatic bis(silyl ether)s **3a** and **3c** led to four different polysulfamates with alternating hard‐soft units (**P1–P4**). When two aryl‐based monomers were combined, slightly higher molar masses were obtained in DMF at 50 °C for polysulfamates **P9** and **P10** featuring alternating hard–hard motifs. Finally, using two aliphatic monomers provided flexible polysulfamates **P5–P8**, albeit with lower molar masses, potentially because of limited solubility—an issue also encountered with aliphatic polysulfamides.^[^ ^37^ ^]^ All synthesized polysulfamates (**P1–P10**) exhibited high thermal stability as shown by thermogravimetric analysis (TGA) with decomposition temperatures (*T*_d_) at 5% weight loss ranging from 190–290 °C. Polymers **P1–P4** and **P9–P10** exhibited a higher *T*_d_ than their aliphatic counterparts **P5–P8**, likely due to the combined influence of greater molar mass and increased aromatic content. Differential scanning calorimetry (DSC) revealed a broad range of glass transition temperatures (*T*_g_) matching the flexibility of the backbone of the macromolecules. Polysulfamate **P8,** featuring highly flexible aliphatic chains showed *T*_g_ of –6 °C, whereas polymers more rigid **P9** and **P10** displayed *T*_g_ values exceeding 100 °C. All solid polysulfamates were found to be amorphous via DSC and powder X‐ray diffraction (Figures S5–S16) except for **P5**, which exhibited some degree of semi‐crystallinity—potentially due to its linear and flexible backbone facilitating interchain hydrogen bonding and self‐assembly.^[^ ^58^ ^]^ With access to a range of polysulfamates, we aimed to evaluate the effect of the isosteric substitution on the physicochemical properties of the polymer. Polyurethane **PU1** containing soft‐hard segments was prepared to serve as benchmark comparison to “macrosisotere” polysulfamate **P1** (Figure 3). To deconvolute any isosteric effect from other structural parameters, **PU1** was synthesized using literature conditions selected to yield a polymer with a molar‐mass distribution comparable to that of **P1** (*M*_w_ = 23.0−30.1 kg mol^−1^, see *Supporting Information*).^[^ ^68^, ^69^ ^]^ TGA analysis revealed a significant increase in thermal stability for **P1** compared to **PU1** (*T*_d_ = 290 °C vs. 210 °C), accompanied by a decrease in the glass transition temperature (*T*_g_ = 70 °C vs. 94 °C, Figure 3a,b). The lack of crystalline or melting transitions in DSC suggests that both **P1** and **PU1** are amorphous, which was further supported by powder X‐ray diffraction measurements (Figure 3c). Films of both **P1** and **PU1** (thickness = 0.6 mm) were subsequently prepared using a hot press at 110 °C under a maximum pressure of 5,000 psi. While slight discoloration of **P1** was observed during hot pressing (Figure 3d)—potentially due to oxidation—SEC and NMR analyses revealed minimal structural changes (Figures S1f and S1g). The mechanical performances of both films were quantitatively evaluated using nanoindentation. **P1** and **PU1** exhibited similar load‐displacement response behavior when subjected to a load of 10,000 µN with a measured displacement of **P1** after unloading indicating a slightly greater deformability (Figure 3e). The hardness (*H*) and reduced Young's modulus (*E*_r_) were then determined from the slope of the unloading segments of the load‐displacement curves using the standard Oliver and Pharr analysis.^[^ ^70^ ^]^ Both materials displayed hardness (*H* = 0.20 GPa for **P1**; *H* = 0.19 GPa for **PU1**) and elastic response (*E*_r_ = 3.82 GPa for **P1**; *E*_r_ = 3.16 GPa for **PU1**) in the same range (Figure 3f). Nanoindentation measurements were also performed on **P5** and **P10** as representative examples of soft–soft and hard–hard polysulfamates, respectively (Figure S1e). The hardness of **P5** (*H* = 0.026 GPa) was expectedly lower than that of **P1** and **P10** (*H* = 0.23 GPa), which can be attributed to its composition of flexible, aliphatic chains that are more prone to deformation (Table S3). Taken together, this data indicates that replacing all –CO– groups with –SO_2_– maintain desirable and tunable mechanical properties.

**Figure 2:**
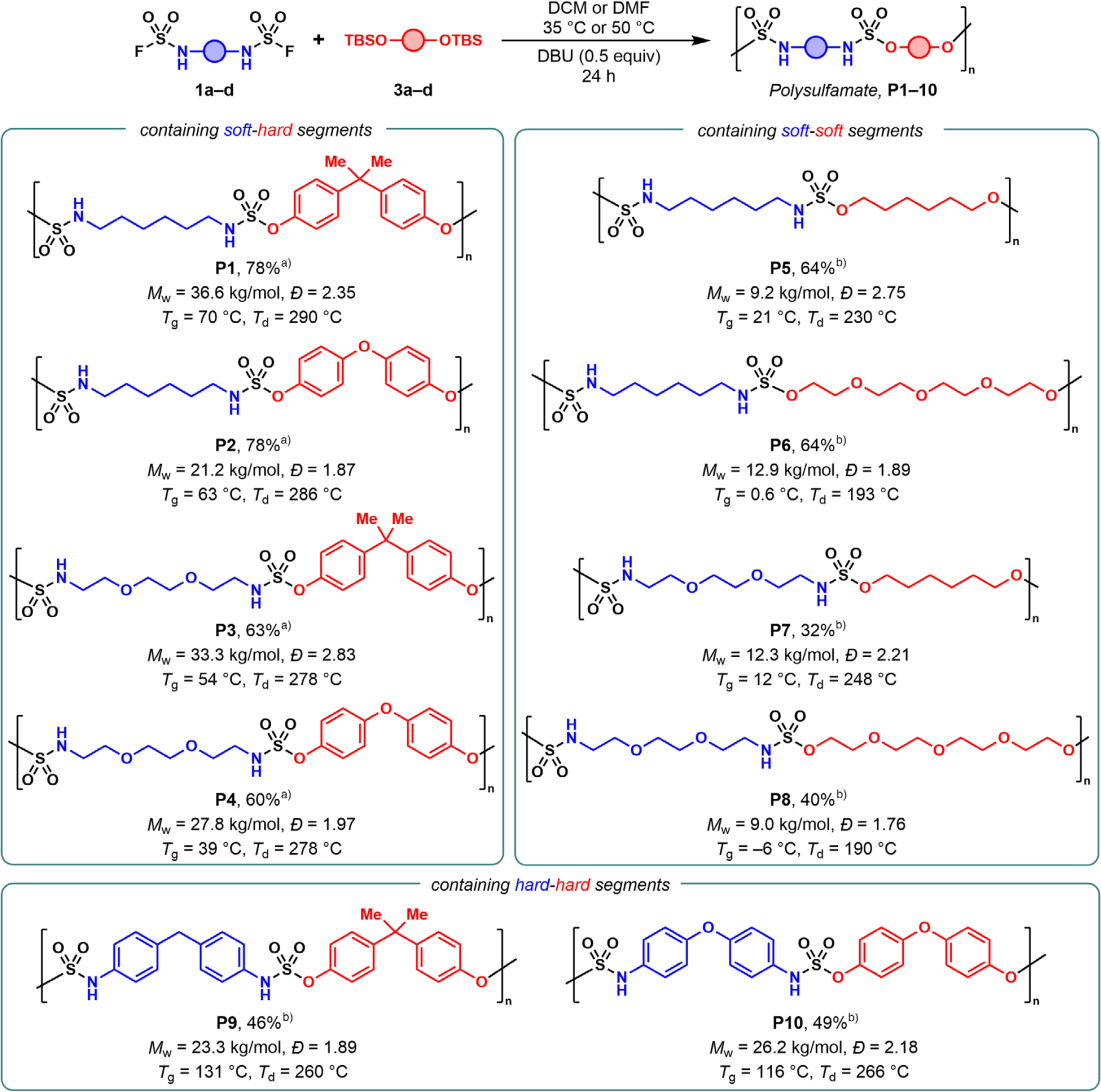
Synthesized polysulfamates via SuFEx click chemistry. *M*_w_’s and *Đ*’s were determined by SEC (DMF + 0.01% LiBr) using poly(methyl methacrylate) standards. *T*_d_ = 5% weight loss temperature. ^a)^ Synthesized in DCM at 35 °C. ^b)^ Synthesized in DMF at 50 °C.

**Figure 3:**
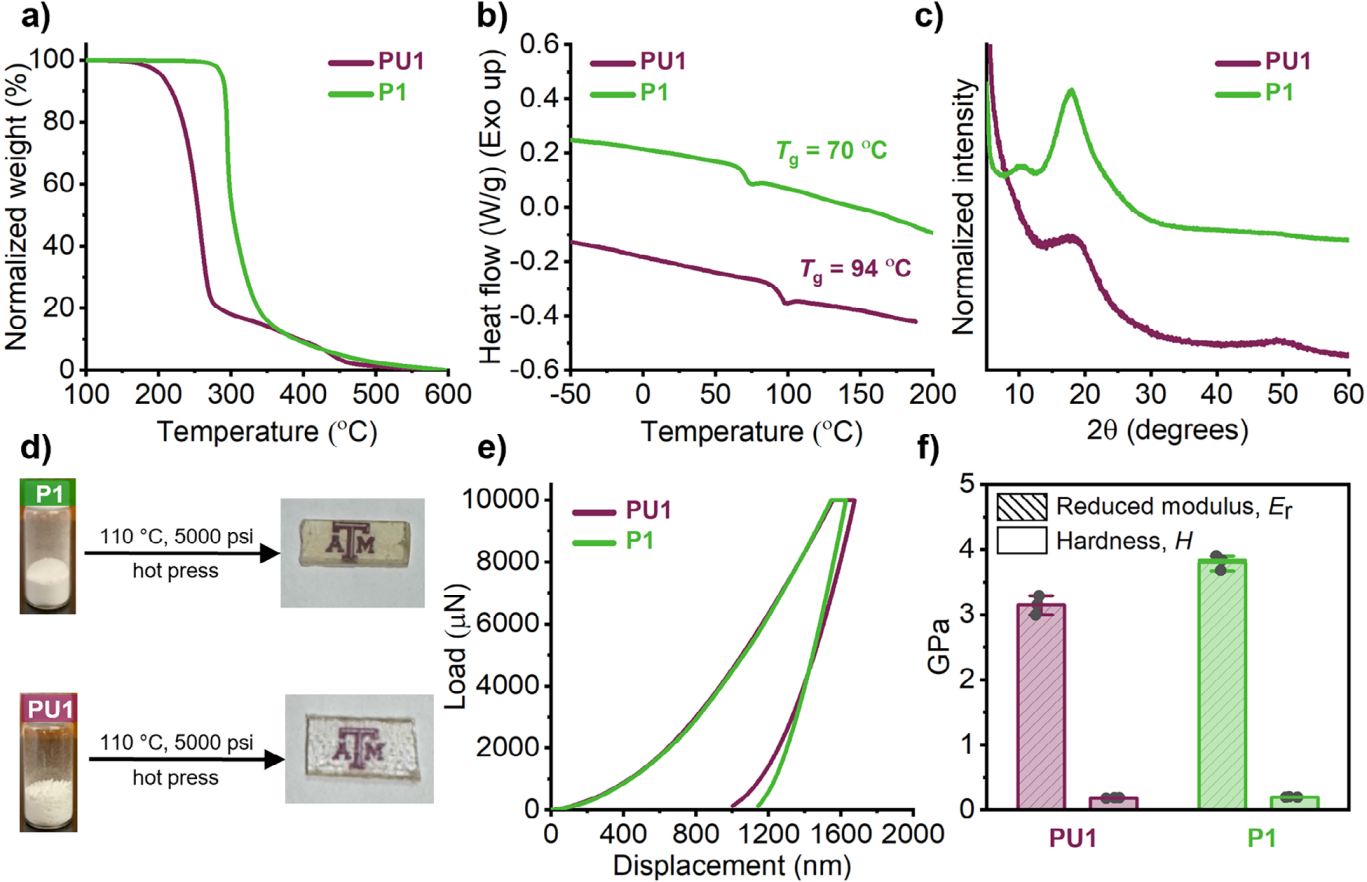
Comparative study of the physicochemical properties of **P1** and **PU1**. a) TGA thermogram; b) DSC thermogram; c) Powder XRD pattern; d) Polymer thin samples prepared via hot press technique; e) load‐displacement curves obtained via nanoindentation; and f) hardness (*H*) and reduced Young's modulus (*E*_r_).

**Figure 4:**
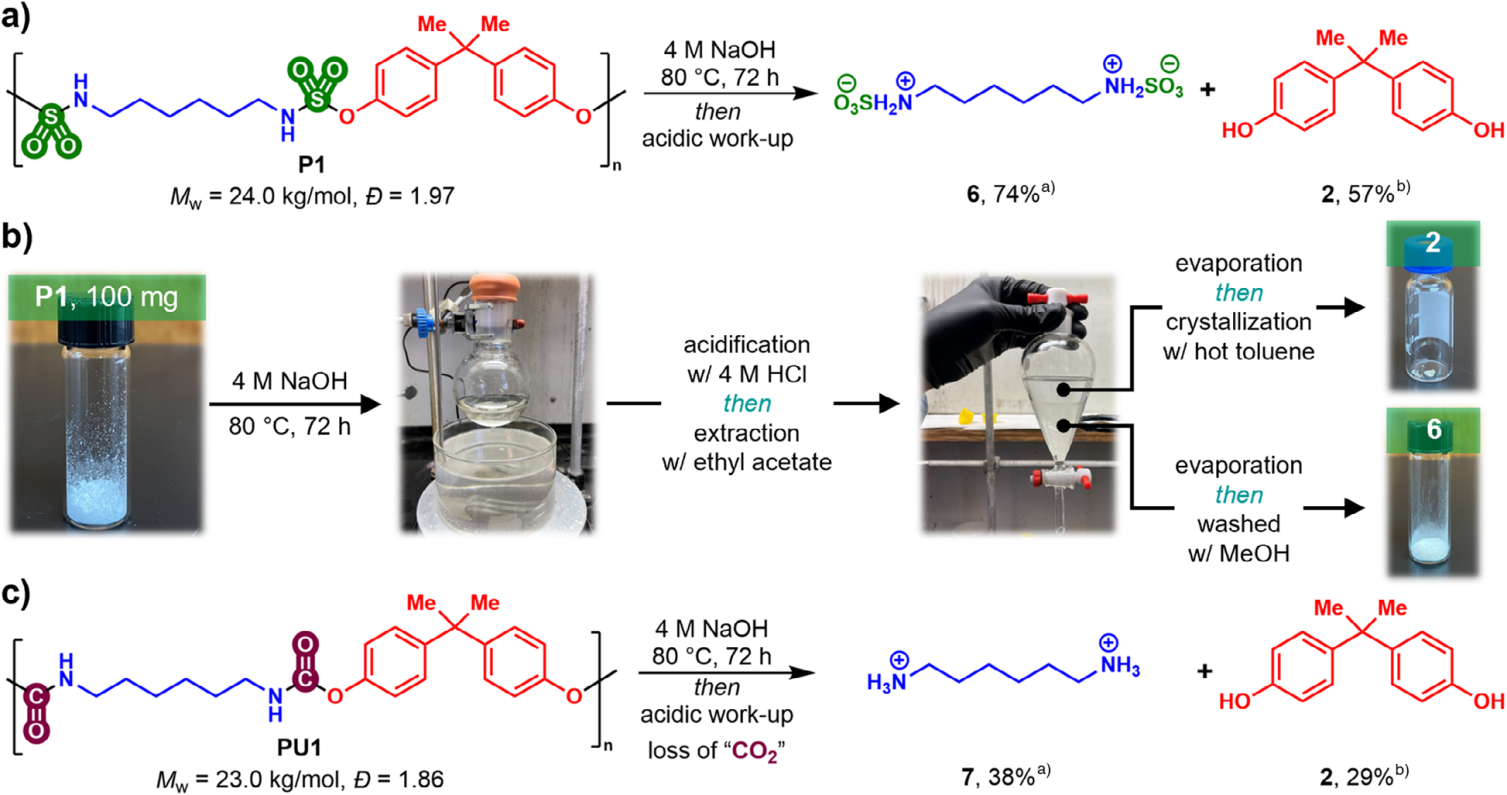
a) Degradation of **P1** in *aqueous* sodium hydroxide; b) Procedure for the isolation of the building blocks; c) Comparative degradation of **PU1** in identical conditions. ^a)^ Yield determined via ^1^H NMR using phenyltrimethylsilane as an internal standard; ^b)^ isolated yield.

The hydrolytic degradability of polysulfamate **P1** was investigated by monitoring the recovery of diol (**2**) in both acidic and basic aqueous media (Table S4). Interestingly, polysulfamates were found to be less prone to acidic degradation compared to polysulfamides, and multiple side products were observed in *aq*. H_2_SO_4_ (C = 6 M). In contrast, clean breakdown of **P1** was observed in aqueous NaOH (C = 4 M) at 60 °C, resulting in up to 50% recovery of bisphenol A (**2**). Raising the temperature to 80 °C further enhanced the recovery yield of **2** to 57%; however, increasing it to 100 °C resulted in the formation of side products, complicating the purification of **2**. With optimized degradation conditions in hand, a protocol was devised to isolate both components of polysulfamate **P1** (Figure 4a). Following basic hydrolysis, the aqueous mixture was acidified to pH ∼3 using HCl and diol **2** was extracted using EtOAc and further purified via recrystallization in hot toluene (Figure 4b). Evaporation of the aqueous phase delivered bis(sulfamic acid) **6** in 74% yield, presumably in its zwitterionic form, as evidenced by NMR and IR spectroscopies combined with HRMS analysis.^[^ ^71^ ^]^ The stability of the sulfamic acid motif is in sharp contrast with the analogous carbamic acid that undergo decarboxylation in the hydrolysis of PUs. Indeed, degradation of **PU1** following the same protocol led to 29% of bisphenol A (**2**) along 38% of protonated 1,6‐hexanediamine (**7**) through loss of 2 equivalents of CO_2_ (Figure 4c). Importantly, **PU1** underwent incomplete degradation, with particles remaining in suspension (Figure S4a). Monitoring the degradation over 72 h in triplicate confirmed the improved degradation kinetics of **P1** vs. **PU1** in basic aqueous conditions (Figure S4b). Soft‐hard PU analogs **P2**–**P4** led to similar recovery yields (Table S5), whereas aliphatic soft‐soft **P5**–**P8** were much less prone to degradation under these conditions. This difference likely stems from the decreased nucleofugality of aliphatic diols and bis(amines), as previously observed in aliphatic polysulfamides.^[^ ^37^ ^]^ Finally, hard‐hard **P9** and **P10** could be degraded as well, albeit with lower recovery yields, potentially due to side reactions.

In summary, the development of SuFEx polymerization of bis(sulfamoyl fluoride)s and bis(silyl ether)s enabled the synthesis of ten polysulfamates with diverse backbones, high thermal stability, and tunable glass transition temperatures starting from commercially available and inexpensive building blocks. A comparative examination of the degradability profile, crystallinity, and thermomechanical performance of a polyurethane (PU) and a polysulfamate differing only by the presence of a –CO– or –SO_2_– motif in the repeating unit suggests that polysulfamates hold promise as a sustainable alternative to PUs, with enhanced degradability, increased thermal stability, and preserved mechanical properties. More broadly, the isosteric replacement of a carbonyl group with a sulfonyl analog in macromolecular systems—a strategy termed “macroisostere”—may guide the design of other families of degradable plastic replacements.

## Supporting Information

The authors have cited additional references within the Supporting Information.^[^ ^72^ ^]^

## Conflict of Interests

The authors declare no conflict of interest.

## Supporting information



Supporting Information

## Data Availability

The data that support the findings of this study are available in the supplementary material of this article.

## References

[1] D. E. Fagnani , J. L. Tami , G. Copley , M. N. Clemons , Y. D. Y. L. Getzler , A. J. McNeil , ACS Macro Lett. 2021, 10, 41–53. 35548997 10.1021/acsmacrolett.0c00789

[2] R. K. Jha , B. J. Neyhouse , M. S. Young , D. E. Fagnani , A. J. McNeil , Chem. Sci. 2024, 15, 5802–5813. 38665509 10.1039/d3sc06758k PMC11041365

[3] A. P. Dove , M. Hong , J. Lamb , H. Sardon , J. Polym. Sci. 2022, 60, 3253–3255.

[4] M. B. Wandel , C. A. Bell , J. Yu , M. C. Arno , N. Z. Dreger , Y.‐H. Hsu , A. Pitto‐Barry , J. C. Worch , A. P. Dove , M. L. Becker , Nat. Commun. 2021, 12, 446. 33469013 10.1038/s41467-020-20610-5 PMC7815890

[5] J. C. Worch , A. P. Dove , Acc. Chem. Res. 2022, 55, 2355–2369. 36006902 10.1021/acs.accounts.2c00293 PMC9454099

[6] J. Kern , Y. Su , W. Gutekunst , R. Ramprasad , Npj Comput. Mater. 2025, 11, 182.

[7] C. Atasi , J. Kern , R. Ramprasad , J. Chem. Inf. Model. 2024, 64, 9249–9259. 39625382 10.1021/acs.jcim.4c01530 PMC11683875

[8] N. A. Meanwell , J. Agric. Food Chem. 2023, 71, 18087–18122. 36961953 10.1021/acs.jafc.3c00765

[9] F. M. de Souza , P. K. Kahol , R. K. Gupta , ACS Symp. Ser. 2021, 1380, 1–24.

[10] Fortune business insights, www.fortunebusinessinsights.com/industry-reports/polyurethane-pu-market-101801.(accessed May 2025).

[11] Statista https://www.statista.com/statistics/720449/global‐polyurethane-market-size-forecast/#:~:text=The%20market%20value%20of%20polyurethane,billion%20U.S.%20dollars%20in%202022. (accessed May 2025).

[12] J. O. Akindoyo , M. D. H. Beg , S. Ghazali , M. R. Islam , N. Jeyaratnam , A. R. Yuvaraj , RSC Adv. 2016, 6, 114453–114482.

[13] C. O. Adetunji , O. T. Olaniyan , O. A. Anani , A. Inobeme , J. T. Mathew , ACS Symp. Ser. 2021, 1380, 393–411.

[14] A. Kemona , M. Piotrowska , Polym. J. 2020, 12, 1752. 10.3390/polym12081752 PMC7464512 32764494

[15] C. Liang , U. R. Gracida‐Alvarez , E. T. Gallant , P. A. Gillis , Y. A. Marques , G. P. Abramo , T. R. Hawkins , J. B. Dunn , Environ. Sci. Technol 2021, 55, 14215–14224. 34618441 10.1021/acs.est.1c03654

[16] S. Kim , K. Li , A. Alsbaiee , J. P. Brutman , W. R. Dichtel , Adv. Mater. 2023, 35, 2305387. 10.1002/adma.202305387 37548061

[17] D. T. Sheppard , K. Jin , L. S. Hamachi , W. Dean , D. J. Fortman , C. J. Ellison , W. R. Dichtel , ACS Cent. Sci. 2020, 6, 921–927. 32607439 10.1021/acscentsci.0c00083 PMC7318067

[18] B. Liu , Z. Westman , K. Richardson , D. Lim , A. L. Stottlemyer , T. Farmer , P. Gillis , N. Hooshyar , V. Vlcek , P. Christopher , M. M. Abu‐Omar , ACS Sustainable Chem. Eng. 2024, 12, 4435–4443. 38516400 10.1021/acssuschemeng.3c07040 PMC10952008

[19] G. Rossignolo , G. Malucelli , A. Lorenzetti , Green Chem. 2024, 26, 1132–1152.

[20] Depolymerization (Polyurethane Chemical Recycling), https://corporate.dow.com/en-us/purpose-in-action/circular-economy/renuva-program.html (accessed: May 2025).

[21] Global corporate website, https://www.covestro.com/press/covestro‐develops‐innovative‐recycling‐technologies/ (accessed: May 2025).

[22] L. R. Mahoney , S. A. Weiner , F. C. Ferris , Environ. Sci. Technol 1974, 8, 135–139.

[23] G. A. Campbell , W. C. Meluch , Environ. Sci. Technol 1976, 10, 182–185.

[24] A. Kumar , N. von Wolff , M. Rauch , Y.‐Q. Zou , G. Shmul , Y. Ben‐David , G. Leitus , L. Avram , D. Milstein , J. Am. Chem. Soc. 2020, 142, 14267–14275. 32706584 10.1021/jacs.0c05675 PMC7441490

[25] V. Zubar , A. T. Haedler , M. Schütte , A. S. K. Hashmi , T. Schaub , ChemSusChem. 2022, 15, e202101606. 34342135 10.1002/cssc.202101606

[26] W. Zhou , P. Neumann , M. Al Batal , F. Rominger , A. S. K. Hashmi , T. Schaub , ChemSusChem 2021, 14, 4176–4180. 33174664 10.1002/cssc.202002465

[27] L. Gausas , S. K. Kristensen , H. Sun , A. Ahrens , B. S. Donslund , A. T. Lindhardt , T. Skrydstrup , JACS Au 2021, 1, 517–524. 34467313 10.1021/jacsau.1c00050 PMC8395660

[28] J. Banik , D. Chakraborty , M. Rizwan , A. H. Shaik , M. R. Chandan , Waste Manage. Res. 2023, 41, 1063–1080. 10.1177/0734242X221146082 36644994

[29] T. Tanaka , T. Yokoyama , Y. Yamaguchi , J. Polym. Sci. Part A: Polym. Chem. 1968, 6, 2137–2152.

[30] Y.‐H. Hsu , D. Luong , D. Asheghali , A. P. Dove , M. L. Becker , Biomacromolecules 2022, 23, 1205–1213. 35044744 10.1021/acs.biomac.1c01473

[31] M. Kubicki , P. W. Codding , S. A. Litster , M. B. Szkaradziñska , H. A. R. Bassyouni , J. Mol. Struct. 1999, 474, 255–265.

[32] L. Gavernet , J. L. Gonzalez Funes , L. B. Blanch , G. Estiu , A. Maresca , C. T. Supuran , J. Chem. Inf. Model. 2010, 50, 1113–1122. 20481572 10.1021/ci100112s

[33] A. C. Brooks , L. Martin , P. Day , E. B. Lopes , M. Almeida , K. Kikuchi , W. Fujita , K. Sasamori , H. Aktusu , J. D. Wallis , Dalton Trans. 2013, 42, 6645–6654. 23487259 10.1039/c3dt32430c

[34] I. Chataigner , C. Panel , H. Gérard , S. R. Piettre , Chem. Commun. 2007, 3288–3290. 10.1039/b705034h 17668103

[35] W. J. Spillane , J. A. Barry , F. L. Scott , J. Chem. Soc., Perkin Trans. 2. 1973, 481–483.

[36] R. W. Kulow , J. W. Wu , C. Kim , Q. Michaudel , Chem. Sci. 2020, 11, 7807–7812. 34094153 10.1039/d0sc03606d PMC8163303

[37] J. W. Wu , R. W. Kulow , M. J. Redding , A. J. Fine , S. M. Grayson , Q. Michaudel , ACS Polym. Au 2023, 3, 259–266. 37334193 10.1021/acspolymersau.2c00060 PMC10273414

[38] D. R. Edwards , R. Wolfenden , J. Org. Chem. 2012, 77, 4450–4453. 22486328 10.1021/jo300386u PMC3345139

[39] During the preparartion of this manuscript, a synthesis of disubstituted polysulfamates (—R_2_NSO_2_O—) was reported: X. Ma , P. Liang , Z. Zhao , J. Chen , X. Wang , Y. Zhou , X. Jiang , W. Zhu , Polym. Chem. 2025, 16, 1578–1583.

[40] J. Dong , L. Krasnova , M. G. Finn , K. B. Sharpless , Angew. Chem. Int. Ed. 2014, 53, 9430–9448. 10.1002/anie.201309399 25112519

[41] L. Xu , J. Dong , in Click Chemistry in Polymer Science: Designs to Applications *, Vol*. 39, (Eds.: N. K. Singha , P. Mondal , R. Hoogenboom ), Royal Society of Chemistry, 2024, pp. 156–176.

[42] D. Zeng , W.‐P. Deng , X. Jiang , Natl. Sci. Rev. 2023, 10, nwad123. 10.1093/nsr/nwad123 PMC10335383 37441224

[43] L. Xu , P. Wu , J. Dong , in Synthetic Polymer Chemistry: Innovations and Outlook (Eds.: Z. Zhao , R. Hu , A. Qin , B. Z. Tang ), The Royal Society of Chemistry, 2019, pp. 1–31.

[44] A. S. Barrow , C. J. Smedley , Q. Zheng , S. Li , J. Dong , J. E. Moses , Chem. Soc. Rev. 2019, 48, 4731–4758. 31364998 10.1039/c8cs00960k

[45] J. A. Homer , L. Xu , N. Kayambu , Q. Zheng , E. J. Choi , B. M. Kim , K. B. Sharpless , H. Zuilhof , J. Dong , J. E. Moses , Nat. Rev. Method. Prim. 2023, 3, 58. PMC11171465 38873592

[46] M. P. Kim , M. K. Sahoo , J.‐H. Chun , S. Y. Hong , Synth. 2024, 57, 1551–1568.

[47] M. P. Kim , S. Kayal , C. Hwang , J. Bae , H. Kim , D. G. Hwang , M. H. Jeon , J. K. Seo , D. Ahn , W. Lee , S. Seo , J.‐H. Chun , Y. Yu , S. Y. Hong , Nat. Commun. 2024, 15, 3381. 38643182 10.1038/s41467-024-47567-z PMC11032359

[48] C. Yang , J. P. Flynn , J. Niu , Angew. Chem. Int. Ed. 2018, 57, 16194–16199. 10.1002/anie.201811051 30326185

[49] H. Fan , Y. Ji , Q. Xu , F. Zhou , B. Wu , L. Wang , Y. Li , J. Lu , ChemPlusChem 2018, 83, 407–413. 31957370 10.1002/cplu.201800067

[50] S. Li , G. Li , B. Gao , S. P. Pujari , X. Chen , H. Kim , F. Zhou , L. M. Klivansky , Y. Liu , H. Driss , D.‐D. Liang , J. Lu , P. Wu , H. Zuilhof , J. Moses , K. B. Sharpless , Nat. Chem. 2021, 13, 858–867. 34400816 10.1038/s41557-021-00726-x PMC8713280

[51] H. Kim , J. Zhao , J. Bae , L. M. Klivansky , E. A. Dailing , Y. Liu , J. R. Cappiello , K. B. Sharpless , P. Wu , ACS Cent. Sci. 2021, 7, 1919–1928. 34841062 10.1021/acscentsci.1c01015 PMC8614101

[52] D.‐D. Liang , S. P. Pujari , M. Subramaniam , M. Besten , H. Zuilhof , Angew. Chem. Int. Ed. 2022, 61, e202116158. 10.1002/anie.202116158 PMC9303861 34919320

[53] H. Wan , Q. Xu , J. Wu , C. Lian , H. Liu , B. Zhang , J. He , D. Chen , J. Lu , Angew. Chem. Int. Ed. 2022, 61, e202208577. 10.1002/anie.202208577 35751405

[54] J. Dong , K. B. Sharpless , L. Kwisnek , J. S. Oakdale , V. V. Fokin , Angew. Chem. Int. Ed. 2014, 53, 9466–9470. 10.1002/anie.201403758 PMC4442796 25100330

[55] H. Wan , S. Zhou , P. Gu , F. Zhou , D. Lyu , Q. Xu , A. Wang , H. Shi , Q. Xu , J. Lu , Polym. Chem. 2020, 11, 1033–1042.

[56] B. Gao , L. Zhang , Q. Zheng , F. Zhou , L. M. Klivansky , J. Lu , Y. Liu , J. Dong , P. Wu , K. B. Sharpless , Nat. Chem. 2017, 9, 1083–1088. 29064495 10.1038/nchem.2796 PMC5972039

[57] H. Wang , F. Zhou , G. Ren , Q. Zheng , H. Chen , B. Gao , L. Klivansky , Y. Liu , B. Wu , Q. Xu , J. Lu , K. B. Sharpless , P. Wu , Angew. Chem. Int. Ed. 2017, 56, 11203–11208. 10.1002/anie.201701160 PMC5665377 28792119

[58] Z. Wu , J. W. Wu , Q. Michaudel , A. Jayaraman , Macromolecules 2023, 56, 5033–5049. 38362140 10.1021/acs.macromol.3c01093 PMC10865372

[59] A. F. J. van den Boom , M. Subramaniam , H. Zuilhof , Org. Lett. 2022, 24, 8621–8626. 36383144 10.1021/acs.orglett.2c03421 PMC9724081

[60] Y. Chao , A. Krishna , M. Subramaniam , D.‐D. Liang , S. P. Pujari , A. C.‐H. Sue , G. Li , F. M. Miloserdov , H. Zuilhof , Angew. Chem. Int. Ed. 2022, 61, e202207456. 10.1002/anie.202207456 PMC9540147 35819248

[61] T. Guo , G. Meng , X. Zhan , Q. Yang , T. Ma , L. Xu , K. B. Sharpless , J. Dong , Angew. Chem. Int. Ed. 2018, 57, 2605–2610. 10.1002/anie.201712429 29276888

[62] W. J. Spillane , F. A. McHugh , P. O. Burke , J. Chem. Soc., Perkin Trans. 2, 1998, 13–18.

[63] T. Guo , W. Wang , L. Xu , J. Dong , Synth. 2025, 57, 978–990.

[64] S. Mahapatra , C. P. Woroch , T. W. Butler , S. N. Carneiro , S. C. Kwan , S. R. Khasnavis , J. Gu , J. K. Dutra , B. C. Vetelino , J. Bellenger , C. W. am Ende , N. D. Ball , Org. Lett. 2020, 22, 4389–4394. 32459499 10.1021/acs.orglett.0c01397 PMC7294807

[65] M. Wei , D. Liang , X. Cao , W. Luo , G. Ma , Z. Liu , L. Li , Angew. Chem. Int. Ed. 2021, 60, 7397–7404. 10.1002/anie.202013976 33337566

[66] C. J. Smedley , J. A. Homer , T. L. Gialelis , A. S. Barrow , R. A. Koelln , J. E. Moses , Angew. Chem. Int. Ed. 2022, 61, e202112375. 10.1002/anie.202112375 PMC8867595 34755436

[67] V. Gembus , F. Marsais , V. Levacher , Synlett 2008, 2008, 1463–1466.

[68] H. Sardon , A. C. Engler , J. M. W. Chan , J. M. García , D. J. Coady , A. Pascual , D. Mecerreyes , G. O. Jones , J. E. Rice , H. W. Horn , J. L. Hedrick , J. Am. Chem. Soc. 2013, 135, 16235–16241. 24083673 10.1021/ja408641g

[69] A. Basterretxea , Y. Haga , A. Sanchez‐Sanchez , M. Isik , L. Irusta , M. Tanaka , K. Fukushima , H. Sardon , Eur. Polym. J. 2016, 84, 750–758.

[70] W. C. Oliver , G. M. Pharr , J. Mater. Res. 1992, 7, 1564–1583.

[71] W. Spillane , J.‐B. Malaubier , Chem. Rev. 2014, 114, 2507–2586. 24341435 10.1021/cr400230c

[72] A. Abri , S. Ranjdar , J. Chin. Chem. Soc. 2014, 61, 929–934.

