# Supplementary material for: Polysulfamates as “Macroisosteres” of Polyurethanes with Improved Degradability
Source: Angew Chem Int Ed Engl. 2025 Aug 22;64(39):e202510841. doi: 10.1002/anie.202510841 (PMC12455453; doi:10.1002/anie.202510841)
Supplement: Supplementary file 1 — Supporting Information [file ANIE-64-e202510841-s001.pdf]

Polysulfamates as “Macroisosteres” of Polyurethanes with Improved Degradability

Srutashini Das,[a]† Katarzyna Doktor,[a]† Biswajit Saha,[a] Felipe Cesar Sousa e Silva,[a] Rachel M. Wynn,[a] Quentin Michaudel[a],[b]*

[a] Department of Chemistry, Texas A&M University, College Station, Texas 77843, United States

[b] Department of Materials Science and Engineering, Texas A&M University, College Station, Texas 77843, United States

Supporting Information

Table of Contents

1. General Reagent Information S2
2. General Analytical Information S2–S3
3. Experimental Procedure S4
   - 3.1. Synthesis of bis (sulfamoyl fluoride)s S4–S5
   - 3.2. Synthesis of bis (silyl ether)s S5–S6
   - 3.3. Synthesis of bis(fluorosulfonate) S6
   - 3.4. Synthesis of polysulfamates S7–12
   - 3.5. Synthesis of polyurethane S12
   - 3.6. Mechanical Properties: polysulfamate *vs* polyurethane S13–S16
   - 3.7. Degradation study: polysulfamate *vs* polyurethane S17–S24
4. Characterization of polymers S25–S37
5. NMR spectroscopy of synthesized monomers and polymers S38–S53
6. References S54

1. General Reagent Information

All reactions were carried out under an inert nitrogen atmosphere with dry solvents under anhydrous conditions unless otherwise stated. Dry dichloromethane (DCM), *N,N*-dimethyl-formamide (DMF) and acetonitrile (MeCN) were obtained by passing the previously degassed solvents through activated alumina columns. Reagents were purchased at the highest commercial quality and used without further purification, unless otherwise stated. 1,8-Diazabicyclo[5.4.0]undec-7-ene (DBU) was purified by vacuum distillation with calcium hydride (CaH_2) and stored over activated 4 Å molecular sieves under nitrogen atmosphere inside a glovebox. SuFEx-IT was synthesized following previously reported procedure.[36] Reactions were monitored by thin layer chromatography (TLC) carried out on 250 µm SiliCycle SiliaPlate™ silica plates (F254), using UV light as the visualizing agent and an acidic solution of *p*-anisaldehyde and heat, or $KMnO_4$ and heat, or ninhydrin and heat as developing agents. Flash silica gel chromatography was performed using SiliCycle SiliaFlash® Irregular Silica Gel (60 Å, particle size 40–063 µm). Polymers were isolated after precipitation using an Eppendorf Model 5804 centrifuge and dried using a VWR Model 1410 vacuum oven.

2. General analytical information

Size-exclusion chromatography (SEC) of polymer samples was performed by EcoSEC Elite® HLC–8420 GPC with refractive index (RI) and column TSKgel® GMH_{HR}-H sequence at a flow rate of 0.35 mL/min. *N*,*N*-dimethylformamide (DMF) with 0.01% LiBr was used as eluent. Weight-average molecular weight (M_w), and dispersities (*Đ*) were calculated from RI chromatograms against poly(methyl methacrylate) (PMMA) standards.

Nuclear magnetic resonance (NMR) ^{1}H NMR spectra were recorded on two systems: Bruker Avance NEO 400 MHz and a Bruker Avance 500 MHz; ^{13}C NMR spectra were recorded on a Bruker Avance 500 MHz; ^{19}F NMR spectra were recorded on a Bruker Avance NEO 400 MHz. All ^{1}H NMR and ^{13}C NMR spectra were calibrated using residual deuterated solvent as an internal reference ($CDCl_3$ @ 7.26 ppm ^{1}H NMR, 77.16 ppm ^{13}C NMR; d_6-DMSO @ 2.50 ppm ^{1}H NMR, 39.52 ppm ^{13}C NMR; CD_3OD @ 3.31 ppm ^{1}H NMR, 49.00 ppm ^{13}C NMR; $(CD_3)_2CO$ @ 2.05 ppm ^{1}H NMR, 29.84 ppm ^{13}C NMR). The following abbreviations were used to explain NMR peak multiplicities: s = singlet, d = doublet, t = triplet, q = quartet, m = multiplet, br = broad.

Fourier transform–infrared (FT-IR) spectra were acquired using a JASCO FTIR spectrometer, model FTIR-4600LE, in the ATR-transmittance mode with a resolution of 2 cm^{-1} by averaging 16 scans in the range of 4000–500 cm^{-1}.

Powder X-Ray diffractions (PXRD) patterns were collected using Bruker D8 Endeavor diffractometer at room temperature (Cu-Kα radiation, fixed divergence slit 0.4 mm, sample to anti-air scatter slit distance of 2 mm, LynxEye XE-T detector and PSD opening of 4°) in the two-theta (2θ) range 3-70° with a step size and time of 0.03° and 0.5 s respectively. Basic analyses were carried out using Bruker Diffrac.EVA software.

Thermogravimetric analysis (TGA) was performed on a TA Instruments TGA 5500 Thermogravimetric Analyzer or an Instruments Q500 Analyzer. Typically, samples (3-5 mg) were heated at 20 °C/min to 100 ºC followed by an isothermal period of 10 min and then heated at 20 ºC/min to 600 °C under nitrogen. Data were processed using Universal Analysis 2000 for windows software.

Differential scanning calorimetry (DSC) was performed using a TA Instruments DSC 2500. Samples (5–8 mg) were sealed in aluminum pans and cooled to –80 ºC at 10 ºC/min, followed by an isothermal period of 3 min. The samples were then heated to a temperature below their decomposition point (defined as 5.0% weight loss) at 10 ºC/min, followed by an isothermal period of 3 min. This process was repeated three times. Thermograms were taken from the second heating cycle, and the data were processed using TA Instruments TRIOS for Windows software.

Nanoindentation was performed using a Bruker TI 950 Triboindenter equipped with a diamond Berkovich indenter tip. The tip shape was calibrated to a fused quartz standard using the Oliver and Pharr methodology prior to testing.[70] 5 x 5 Arrays of indentations with 20 μm spacing between neighboring indents were performed. All indents were load-controlled with a maximum applied load of 10000 μN, a loading and holding time of 10 s, and an unloading time of 2 s. The hardness (H) and reduced modulus (E_r) were then calculated from the unloading segments of the load-displacement curves using the standard Oliver and Pharr analysis.

3. Experimental procedure

3.1 Synthesis of bis(sulfamoyl fluoride)s

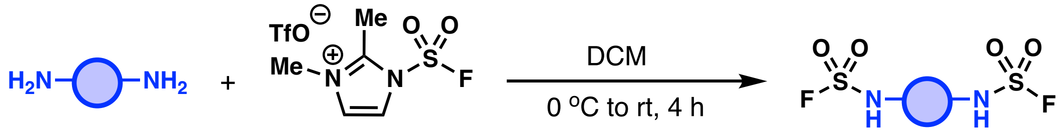

Previously known bis(sulfamoyl fluoride) **1a** and **1b** were synthesized from commercially available diamines and SuFEx-IT **(S1)** as previously reported. The spectroscopic data of the product is identical to that reported in the literature.[36, 58]

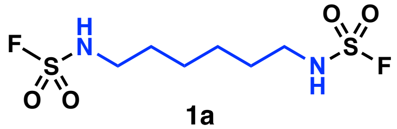

1a

^{1}H NMR (CDCl3, 400 MHz) δ: 5.05 (br, 2 H), 3.34–3.29 (m, 4 H), 1.69–1.52 (m, 4 H), 1.44–1.39 (m, 4 H) ppm.

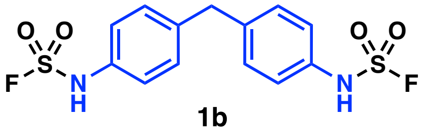

1b

^{1}H NMR (CDCl3, 400 MHz) δ: 7.24–7.19 (m, 8 H), 6.78 (br, 2 H), 3.99 (s, 2 H) ppm.

General Procedure A

Taking 1c as an example

Bis(sulfamoyl fluoride) **1c** was synthesized using a procedure directly adapted from our previous report. [36] **S1** (8.8 g, 27.0 mmol, 2.0 equiv) was added to a solution of 2,2′-(ethylenedioxy) bis(ethylamine) (3.0 g, 20.2 mmol, 1.0 equiv) in MeCN (80.8 mL, C = 0.25 M) at 0 °C. The mixture was then allowed to warm to room temperature and stirred until full consumption of starting material (monitored by TLC, 4 hours in most cases). After completion, the reaction mixture was concentrated under vacuo and diluted with ethyl acetate (20 mL) and washed with 1 M HCl (20 mL). The organic phase was washed with brine (20 mL), dried over $MgSO_4$ and then filtered. The solvent was evaporated *in vacuo* and the crude material was purified by column chromatography to afford the desired product.

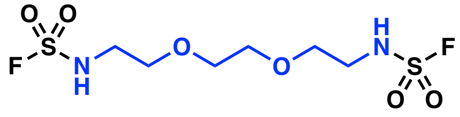

1c

1c was prepared from 2,2′-(ethylenedioxy) bis(ethylamine) (3.0 g, 20.2 mmol) and **S1** (8.8 g, 27.0 mmol) in MeCN following ***general procedure A***. Column chromatography (SiO_2, 30:70 to 50:50 EtOAc:hexanes) afforded **1c** as a colorless viscous oil (3.16 g, 50%).

R_f: 0.47 (50:50 EtOAc:hexanes)

^{1}H NMR ($CDCl_3$, 500 MHz) δ: 6.02 (br, 2 H), 3.70 (t, J = 10 Hz, 8 H), 3.51–3.46 (m, 4 H) ppm.

^{13}C NMR ($CDCl_3$, 126 MHz) δ: 70.8, 69.1, 44.4 ppm.

^{19}F NMR ($CDCl_3$, 470 MHz) δ: 51.0 ppm.

HRMS(+ESI) calc'd for $C_6H_{15}F_2N_2O_6S_2$ $[M+H]^+$ 313.0334, found 313.0330.

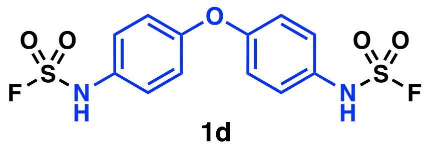

1d

1d was prepared from 4,4′-diaminodiphenyl ether (3.0 g, 14.9 mmol) and **S1** (9.8g, 29.9 mmol) following ***general procedure A*** with a mixture of DCM and DMF (1:1 v/v) instead of MeCN. Column chromatography (SiO_2, 30:70 EtOAc:hexanes) afforded **1d** as a pale off-white solid (2.85 g, 52%).

R_f: 0.45 (30:70 EtOAc:hexanes)

^{1}H NMR ($CDCl_3$, 500 MHz) δ: 7.31(d, *J* = 8.9 Hz, 4 H), 7.05 (d, *J* = 9.0 Hz, 4 H), 6.71 (br, 2 H) ppm.

^{13}C NMR ($CDCl_3$, 126 MHz) δ: 156.4, 129.2, 126.4, 120.4 ppm.

^{19}F NMR ($CDCl_3$, 470 MHz) δ: 50.6 ppm.

HRMS(+ESI) calc'd for $C_{12}H_{11}F_2N_2O_5S_2$ $[M+H]^+$ 362.9915, found 362.9932.

3.2 Synthesis of bis(silyl ether)s: General Procedure B

Taking 3a as an example

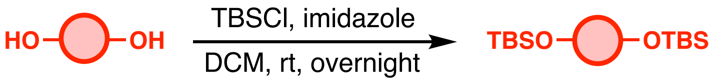

The bis(silyl ether) monomer **3a** was synthesized according to a previously reported procedure, with minor modifications.[54] To a flame-dried round-bottom flask, equipped with a PTFE-coated stirring bar, were added the diol (3.0 g, 13.1 mmol, 1.0 equiv), imidazole (2.3 g, 34.1 mmol, 2.6 equiv), *tert*-butyldimethylsilyl chloride (2.7 g, 31.5 mmol, 2.4 equiv), followed by dichloromethane (DCM) (26.2 mL, C = 0.5 M). The reaction mixture was stirred at room temperature for about 3–4 h. Upon completion of the reaction, the solid residues were removed through filtration and concentrated under reduced pressure. The resulting reaction mixture was then dissolved in EtOAc (~ 20 mL) and sequentially washed with 1 M HCl (3 x 20 mL), saturated $NaHCO_3$ (20 mL) and brine (20 mL). The organic phase was then dried over $MgSO_4$, filtered, and concentrated to obtain the product, which was then subsequently purified using column chromatography or recrystallization (see below).

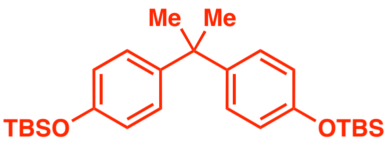

3a

3a was prepared following ***general procedure B***. A few drops of DMF were added to the reaction mixture until bisphenol A was completely dissolved. The removal of the volatiles resulted in the formation of **3a** as a pale-yellow oil, which then formed white solid upon being dried under vacuum. The compound was further purified by recrystallization. It was dissolved in hot methanol, and a few drops of DCM were added until complete dissolution was achieved. The solution was then left to stand undisturbed on the bench to cool and allow partial evaporation of DCM, facilitating crystal formation. The resulting crystals were collected and dried under vacuum (5.5 g, 92%).

The spectroscopic data of the product were identical to those reported in the literature.[54]

^{1}H NMR ($CDCl_3$, 400 MHz) δ: 7.05 (d, *J* = 8.7 Hz, 4 H), 6.71 (d, *J* = 8.6 Hz, 4 H), 1.61 (s, 6 H), 0.97 (s, 18 H), 0.18 (s, 12 H).

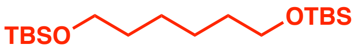

3b

3b was prepared from 1,6-hexane diol (2.0 g, 16.9 mmol) following ***general procedure B***. Column chromatography (SiO_2, 0:100–10:90 EtOAc:hexanes) afforded **3b** a colorless oil (4.9 g, 83%).

The spectroscopic data of the product were identical to those reported in the literature.[72]

^{1}H NMR ($CDCl_3$, 400 MHz) δ: 3.60 (t, J = 6.6 Hz, 4 H), 1.55–1.48 (m, 4 H), 1.35–1.31 (m, 4 H), 0.89 (s, 18 H), 0.04 (s, 12 H) ppm.

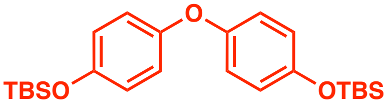

3c

3c was prepared from 4,4'-oxydiphenol (1.5 g, 7.4 mmol) following ***general procedure B***. Column chromatography (SiO_2, 100% hexanes) afforded **3c** as a pale-yellow solid (2.8 g, 90%).

The spectroscopic data of the product were identical to those reported in the literature.[54]

^{1}H NMR ($CDCl_3$, 400 MHz) δ: 6.84 (d, J = 8.9 Hz, 4 H), 6.77 (d, J = 8.8 Hz, 4 H), 0.89 (s, 18 H), 0.18 (s, 12 H) ppm.

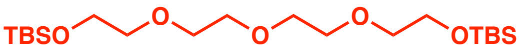

3d

3d was synthesized from tetraethylene glycol (3.0 g, 15.4 mmol) using the ***general procedure B***. Column chromatography (SiO_2, 10:90–20:80 EtOAc:hexanes) afforded **3d** as a colorless oil (6.0 g, 79%).

R_f: 0.6 (30:70 EtOAc:hexanes)

^{1}H NMR ($CDCl_3$, 500 MHz) δ: 3.76 (t, J = 11.0 Hz, 4 H), 3.65 (s, 8 H), 3.55 (t, J = 11.0 Hz, 4 H), 0.89 (s, 18 H), 0.06 (s, 12 H) ppm.

^{13}C NMR ($CDCl_3$, 126 MHz) δ: 72.8, 70.8, 62.9, 26.1, 18.5, –5.1 ppm.

HRMS(+ESI) calc'd for $C_{20}H_{47}O_5Si_2$ $[M+H]^+$ 423.2957, found 423.2950.

3.3 Synthesis of bis(fluorosulfonate)

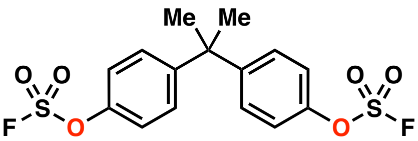

5

Previously known bis(fluorosulfonate) **5** was synthesized from commercially available bisphenol A (**2**) and SO_2F_2 (g). The spectroscopic data of the product is identical to that reported in the literature.[54]

^{1}H NMR ($CDCl_3$, 400 MHz) δ: 7.32–7.25 (m, 8H), 1.70 (s, 6H) ppm.

3.4 Synthesis of polysulfamates

Table S1. Additional Reactions Performed in the Optimization of the Synthesis of P1

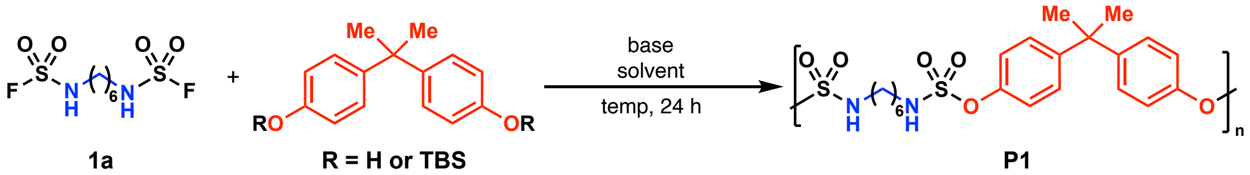

Entry	R	Solvent	Base (equiv)	Temp. (°C)	M_w (kg/mol)	*Đ*
1	H	DCM	DBU (0.5)	rt	1.48	1.85
2	H	DMF	DBU (0.5)	rt	1.27	1.74
3	TBS	MeCN	DBU (0.5)	rt, 12 h → 80, 12 h	20.0	2.15
4	TBS	MeCN	DBU (0.5)	rt, 4 h → 80, 20 h	19.8	2.05
5	TBS	MeCN	DBU (0.5)	80	8.8	1.83
6	TBS	DMF	DBU (0.5)	50	14.5	1.72
7	TBS	DMF	DBU (0.5)	70	13.7	1.74
8	TBS	NMP	DBU (0.5)	rt	10.8	2.10
9	TBS	NMP	DBU (0.5)	80	13.6	2.37
10	TBS	DCM	DBU (1.0)	35	32.2	8.40
11	TBS	DCM	DBU (2.0)	35	Insoluble	
12	TBS	DCM	DABCO (0.5)	35	Insoluble	

Table S2. Comparison of the Synthesis of P1–10 in DCM at 35 °C or DMF at 50 °C

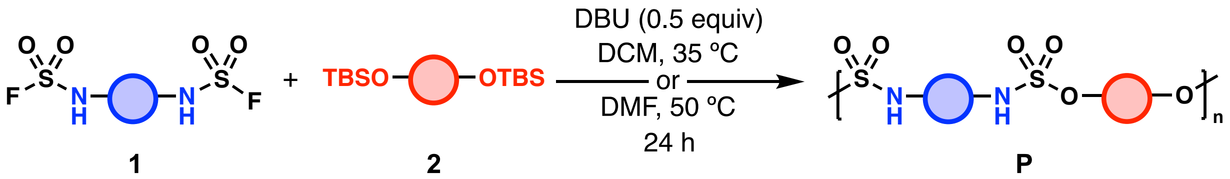

Polymer	DCM at 35 °C		DMF at 50 °C	
	M_w (kg/mol)	*Đ*	M_w (kg/mol)	*Đ*
P1	36.6	2.35	14.5	1.72
P2	21.2	1.87	4.8	7.73
P3	33.3	2.83	5.5	8.80
P4	27.8	1.97	4.6	1.91

P5	4.5	1.75	9.2	2.75
P6	12.5	1.67	12.9	1.89
P7	9.4	1.50	12.3	2.21
P8	16.1[a]	1.28	9.0	1.76
P9	–[b]	–	23.3	1.89
P10	–[b]	–	26.2	2.18

[a] Low yield (<10 %); [b] NMR not promising.

General Procedure C

Taking P1 as an example

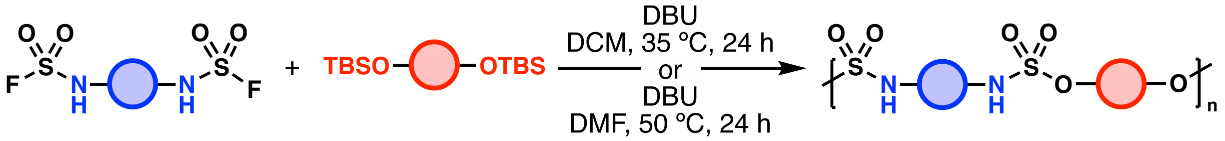

1 **3** **P**

To a flame-dried screw capped reaction tube containing a stir bar, were added bis(sulfamoyl fluoride) **1a** (200 mg, 0.7 mmol, 1.0 equiv) and bis(silyl ether) **3a** (307 mg, 0.7 mmol, 1.0 equiv). The reaction tube was then evacuated under high vacuum and backfilled with nitrogen (three cycles). Anhydrous DCM or DMF (1.4 mL, C = 0.5 M) was added, and the mixture was stirred until complete dissolution of the reactants (~1-2 min). Subsequently, DBU (52 μL, 0.35 mmol, 0.5 equiv) was added, and the reaction mixture was stirred at either 35 ºC (in DCM) or 50 ºC (in DMF). After 24 h, the reaction mixture was precipitated by dropwise addition into a vial containing diethyl ether (~20 mL) under vigorous stirring. The precipitation was performed incrementally through the addition of 10–15 drops of the reaction mixture at a time and decantation of the solvent after each added portion. Following the last decantation, the resulting solid was dried under vacuum at room temperature for 3–4 hours. It was then re-dissolved in DMF and re-precipitated in 1 M HCl solution (~20 mL) portion wise with vigorous stirring and decantation of the HCl solution between each portion. The resulting polymer was dried under a flow of compressed air for about an hour, and then re-dissolved in DMF and subsequently precipitated in water (~20 mL) portion wise with decantation in between each addition to ensure efficient removal of residual DMF and DBU (any residual DMF was further removed by repeatedly washing with diethyl ether). The solid residues were then dried under vacuum at room temperature to deliver the polysulfamate (~18 h). The polymer purity and absence of solvent trace was assessed using NMR spectroscopy. Molecular weight (M_w) and dispersities ($Đ$) were determined using SEC (DMF with 0.01% LiBr using PMMA standards; see general analytical information). This method also enables the gram-scale synthesis of polysulfamates without compromising their molecular weight.

NOTE: For polymers **P1–P4**, conducting the reaction in DCM at 35 °C resulted in the highest molecular weights and yields. In contrast, for polymers **P5–P10**, optimal molecular weights and yields were obtained using DMF at 50 °C.

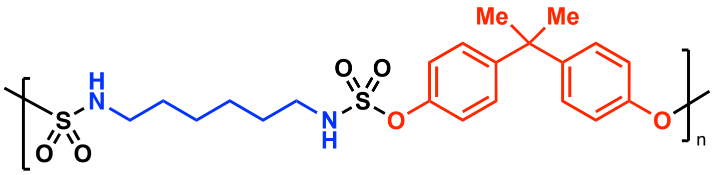

P1

Polysulfamate, **P1** was prepared from **1a** (200 mg, 0.7 mmol) and **3a** (307 mg, 0.7 mmol) following ***general procedure C*** using DCM as a solvent. **P1** was obtained as a white solid (78%)

^{1}H NMR (d_6-DMSO, 500 MHz) δ: 8.28 (br, 2 H), 7.28 (d, J = 8.9 Hz, 4 H), 7.17 (d, J = 8.9 Hz, 4 H), 3.07–3.02 (m, 4 H), 1.64 (s, 6 H), 1.47–1.42 (m, 4 H), 1.28–1.24 (m, 4 H) ppm.

^{13}C NMR (d_6-DMSO, 126 MHz) δ: 148.2, 147.8, 127.8, 121.5, 43.1, 42.0, 30.6, 28.8, 25.5 ppm.

SEC (DMF with 0.01% LiBr, PMMA standards): M_w (kg/mol) = 36.6; $Đ$ = 2.35 (*batch 1*) and M_w = 23.4 kg/mol, $Đ$ = 2.04 (*batch 2*) and M_w = 24.0 kg/mol, $Đ$ = 1.97 (*batch 3*).

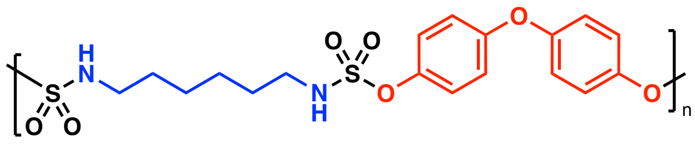

P2

Polysulfamate, **P2** was prepared from **1a** (200 mg, 0.7 mmol) and **3c** (307 mg, 0.7 mmol) following ***general procedure C*** using DCM as a solvent*.* **P2** was obtained as a white solid (78%).

^{1}H NMR (d_6-DMSO, 500 MHz) δ: 8.32 (br, 2 H); 7.30 (d, J = 9.0 Hz, 4 H); 7.10 (d, J = 9.0 Hz, 4 H); 3.06 (app q, J = 7.0 Hz, 4 H), 1.51–1.44 (m, 4 H), 1.30–1.27 (m, 4 H) ppm.

^{13}C NMR (d_6-DMSO, 126 MHz) δ: 154.7; 145.6; 123.7; 119.7; 43.2; 28.8; 25.5 ppm.

SEC (DMF with 0.01% LiBr, PMMA standards): M_w (kg/mol) = 21.2; $Đ$ = 1.87*.*

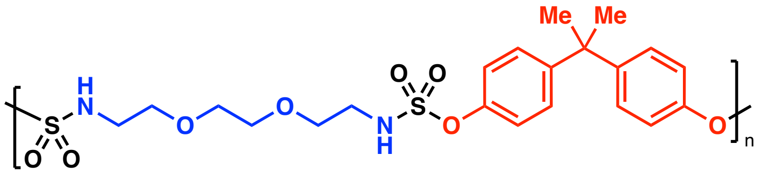

P3

Polysulfamate, **P3** was prepared from **1c** (200 mg, 0.7 mmol) and **3a** (246 mg, 0.7 mmol) following ***general procedure C*** using DCM as a solvent. **P3** was obtained as a colorless solid (63%).

^{1}H NMR (d_6-DMSO, 500 MHz) δ: 8.41 (t, J = 5.6 Hz, 2 H), 7.28 (d, J = 8.8 Hz, 4 H), 7.20 (d, J = 8.9 Hz, 4 H), 3.51 (s, 4 H), 3.48 (t, J = 5.8 Hz, 4 H), 3.20 (app q, J = 5.8 Hz, 4 H), 1.64 (s, 6 H) ppm.

^{13}C NMR (d_6-DMSO, 126 MHz) δ: 148.2, 147.8, 127.8, 121.5, 69.5, 68.6, 43.0, 42.0, 30.3 ppm.

SEC (DMF with 0.01% LiBr, PMMA standards): M_w (kg/mol) = 33.3; $Đ$ = 2.83.

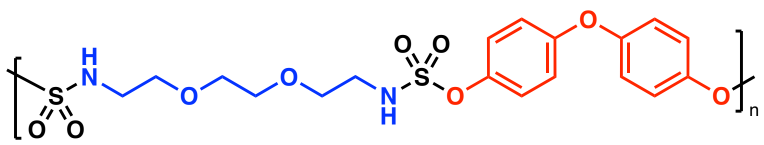

P4

Polysulfamate, **P4** was prepared from **1c** (200 mg, 0.7 mmol) and **3c** (300 mg, 0.7 mmol) following ***general procedure C*** using DCM as a solvent. **P4** was obtained as a colorless solid (60%).

^{1}H NMR (d_6-DMSO, 500 MHz) δ: 8.45 (t, J = 5.5 Hz, 2 H), 7.32 (d, J = 9.1 Hz, 4 H), 7.08 (d, J = 8.9 Hz, 4 H), 3.53–3.49 (m, 8 H), 3.20 (q, J = 5.7 Hz, 4 H) ppm.

^{13}C NMR (d_6-DMSO, 126 MHz) δ: 154.7, 145.5, 123.8, 119.7, 69.5, 68.6, 43.0 ppm.

SEC (DMF with 0.01% LiBr, PMMA standards): M_w (kg/mol) = 27.8; $Đ$ = 1.97.

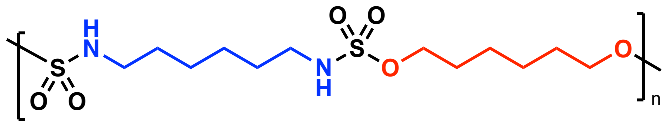

P5

Polysulfamate, **P5** was prepared from **1a** (200 mg, 0.6 mmol) and **3b** (292 mg, 0.6 mmol) following ***general procedure C*** using DMF as a solvent. **P5** was obtained as an off-white solid (64%).

^{1}H NMR (d_6-DMSO, 500 MHz): 7.72 (br, 2 H), 3.99 (t, J = 6.5 Hz, 4 H), 2.90 (app q, J = 6.8 Hz, 4 H), 1.67–1.62 (m, 4 H), 1.48–1.41 (m, 4 H), 1.37–1.34 (m, 4 H), 1.31–1.26 (m, 4 H) ppm.

^{13}C NMR (d_6-DMSO, 126 MHz): 69.1, 42.6, 28.8, 28.1, 25.6, 24.6 ppm.

SEC (DMF with 0.01% LiBr, PMMA standards): M_w (kg/mol) = 9.2; $Đ$ = 2.75.

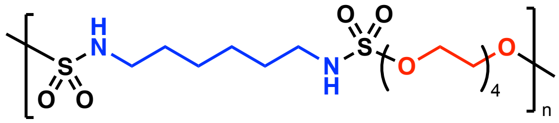

P6

Polysulfamate, **P6** was prepared from **1a** (200 mg, 0.6 mmol) and **3d** (276 mg, 0.6 mmol) following ***general procedure C*** using DMF as a solvent. **P6** was obtained as a colorless viscous oil (64%).

^{1}H NMR (d_6-DMSO, 500 MHz) δ: 7.77 (br, 2 H), 4.08 (t, J = 4 Hz, 4 H), 3.65 (t, J = 4 Hz, 4 H), 3.56–3.52 (m, 8 H), 2.91 (app q, J = 7.1 Hz, 4 H), 1.49–1.43 (m, 4 H), 1.32–1.27 (m, 4 H) ppm.

^{13}C NMR (d_6-DMSO, 126 MHz) δ: 69.7, 68.4, 68.1, 42.6, 28.7, 25.6 ppm.

SEC (DMF with 0.01% LiBr, PMMA standards): M_w (kg/mol) = 12.9; $Đ$ = 1.89.

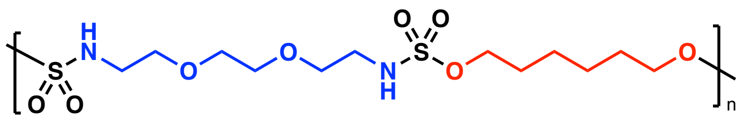

P7

Polysulfamate, **P7** was prepared from **1c** (200 mg, 0.6 mmol) and **3b** (275.6 mg, 0.6 mmol) following ***general procedure C*** using DMF as a solvent. **P7** was obtained as a colorless viscous oil (32%).

^{1}H NMR (d_6-DMSO, 500 MHz) δ: 7.86 (br, 2 H), 4.01 (t, J = 6.5 Hz, 4 H), 3.53 (s, 4 H), 3.48 (t, J = 5.7 Hz, 4 H), 3.07 (app q, J = 5.8 Hz, 4 H), 1.67–1.62 (m, 4 H), 1.39–1.33 (m, 4 H) ppm.

^{13}C NMR (d_6-DMSO, 126 MHz) δ: 69.5, 69.3, 68.7, 42.4, 28.1, 24.6 ppm.

SEC (DMF with 0.01% LiBr, PMMA standards): M_w (kg/mol) = 12.3; $Đ$ = 2.21.

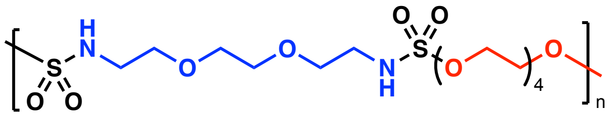

P8

Polysulfamate, **P8** was prepared from **1c** (200 mg, 0.6 mmol) and **3d** (275.6 mg, 0.6 mmol) following ***general procedure C*** using DMF as a solvent. **P8** was obtained as a pale-yellow viscous oil (40%).

^{1}H NMR (d_6-DMSO, 500 MHz) δ: 7.91 (br, 2 H), 4.12 – 4.10 (m, 4 H), 3.66 – 3.64 (m, 4 H), 3.55–3.53 (m, 12 H), 3.48 (t, J = 5.9 Hz, 4 H), 3.08 (app q, J = 5.7 Hz, 4 H) ppm.

^{13}C NMR (d_6-DMSO, 126 MHz) δ: 69.7, 69.5, 68.6, 68.1, 42.4 ppm.

SEC (DMF with 0.01% LiBr, PMMA standards): M_w (kg/mol) = 9.0; $Đ$ = 1.76.

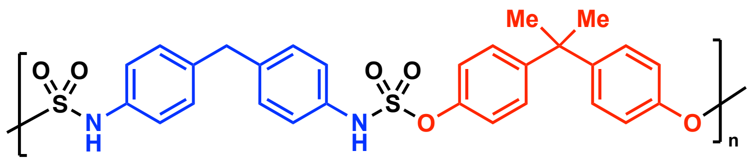

P9

Polysulfamate, **P9** was prepared from **1b** (200 mg, 0.55 mmol) and **3a** (252.1 mg, 0.55 mmol) following ***general procedure C*** using DMF as a solvent. **P9** was obtained as an off-white solid (46%).

^{1}H NMR (d_6-DMSO, 500 MHz) δ: 10.87 (br, 2 H), 7.23–7.17 (m, 8 H), 7.11 (d, J = 8.5 Hz, 4 H), 7.04 (d, J = 8.9 Hz, 4 H), 3.84 (s, 2 H), 1.57 (s, 6 H) ppm.

^{13}C NMR (d_6-DMSO, 126 MHz) δ: 148.7, 147.5, 136.9, 135.0, 129.5, 128.0, 121.4, 119.4, 114.7, 42.1, 30.2 ppm.

SEC (DMF with 0.01% LiBr, PMMA standards): M_w (kg/mol) = 23.3; $Đ$ = 1.89.

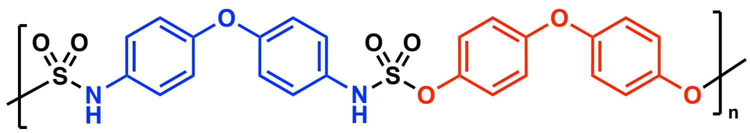

P10

Polysulfamate, **P10** was prepared from **1d** (200 mg, 0.55 mmol) and **3c** (250.8 mg, 0.55 mmol) following ***general procedure C*** using DMF as a solvent. **P10** was obtained as a white solid (49%).

^{1}H NMR (d_6-DMSO, 500 MHz) δ: 10.93 (br, 2 H), 7.25 (d, *J* = 9.0 Hz, 4 H), 7.21 (d, *J* = 9.2 Hz, 4 H), 7.06 (t, *J* = 9.8 Hz, 8 H) ppm.

^{13}C NMR (d_6-DMSO, 126 MHz) δ: 154.9, 153.3, 145.3, 132.3, 123.7, 121.5, 119.9, 119.5 ppm.

SEC (DMF with 0.01% LiBr, PMMA standards): M_w (kg/mol) = 26.2; $Đ$ = 2.18.

3.5 Synthesis of polyurethane (PU1)

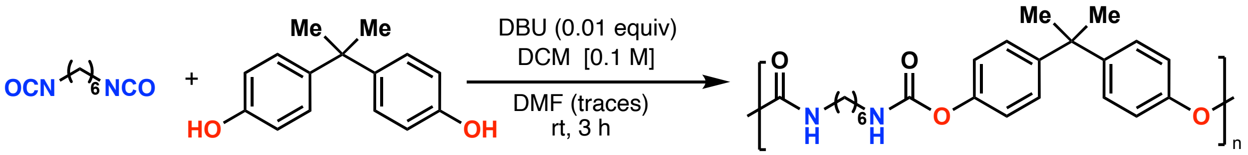

Polyurethane (**PU1**) was synthesized following a reported procedure[69] with slight modifications. To a flame-dried round bottom flask containing a stir bar was added hexamethylene diisocyanate (**S2)** (1.9 mL, 11.9 mmol, 1.0 equiv) and **2** (2.7 g, 11.9 mmol, 1.0 equiv). The flask was then evacuated under high vacuum and backfilled with nitrogen (three cycles). DCM (119 mL, C = 0.1 M) was added to the mixture and stirred for 2-3 min until most of **2** has dissolved. DMF (1.6 ml) was then added to the reaction mixture to help fully dissolve **2**. Upon addition of DBU (17.7 µL, 0.1 mmol, 0.01 equiv), the reaction mixture was stirred at room temperature for 3 h. Precipitation of a white solid was observed over time. The resulting suspension was further precipitated by dropwise and portion wise addition into two 45 mL centrifuge tubes, each containing 30 mL of methanol, followed by centrifugation (10 minutes at 9500 rpm) and removal of the supernatant. This process was repeated until the entire reaction mixture had been transferred. The solid residues were subsequently dried in a vacuum oven at 85 °C overnight. **PU1** was obtained as a white solid in 82% and characterized using NMR spectroscopy and SEC analysis (DMF with 0.01% LiBr using PMMA standards; see general analytical information).

^{1}H NMR (400 MHz, d_6-DMSO) δ: 7.66 (br, 2 H), 7.19 (d, *J* = 8.4 Hz, 4 H), 6.98 (d, *J* = 8.3 Hz, 4 H), 3.05–3.01 (m, 4 H), 1.62 (s, 6 H), 1.47–1.42 (m, 4 H), 1.31–1.28 (m, 4 H) ppm.

^{13}C NMR (126 MHz, d_6-DMSO): 154.3, 148.9, 146.5, 127.1, 121.1, 41.7, 30.5, 29.1, 25.9 ppm.

SEC (DMF with 0.01% LiBr, PMMA standards): M_w (kg/mol) = 23.0; $Đ$ = 1.89 (*batch 1*) and M_w = 30.1 kg/mol, $Đ$ = 2.11 (*batch 2*).

NOTE: To allow triplicate testing of **P1** and **PU1** via nanoindentation and for degradation experiments, several batches were prepared; M_w and $Đ$ of each polymer are specified in the respective analyses.

3.6 Mechanical properties of polysulfamates (P1, P5 and P10) and polyurethane (PU1)

The mechanical properties of polysulfamates **P1**, **P5**, **P10** and polyurethane **PU1** were evaluated using nanoindentation. For these experiments, thin films were prepared via hot pressing technique (*Figure S1a*). The hot press instrument was preheated to 110 °C, and the polymer was placed into a 4 cm × 2 cm × 0.6 mm mold before being subjected to a pressure of maximum 5000 psi. This process involved consecutive cycles of 5–8 minutes of compression followed by evacuation to release any air bubble, repeated approximately 2–3 times for **P1** and shorter cycles of about 2–3 minutes repeated multiple times for **PU1** until all the air bubbles have been removed. Once cooled to room temperature, the films were subsequently visually inspected to ensure the absence of air bubbles. Rectangular samples (approximately 1 cm x 1 cm) were obtained from the films using a sharp cutter. These samples were then glued onto steel AFM disc (1.5 cm in diameter) (*Figure S1b*) which was then subjected into the Bruker TI 950 Triboindenter using a nanoindentation sample stage containing 9 magnetic studs which can hold 9 samples at a time (*Figure S1c*). A maximum load of 10,000 μN was applied with a loading and holding time of 10 s, and an unloading time of 2 s. The hardness (*H*) and reduced modulus (E_r) were then calculated from the unloading segments of the load-displacement curves (*Figure S1d*), following the standard analysis reported by Oliver and Pharr.[70] For each polymer, 3 samples were tested using 25 indents per sample.

(a) (b) (c)

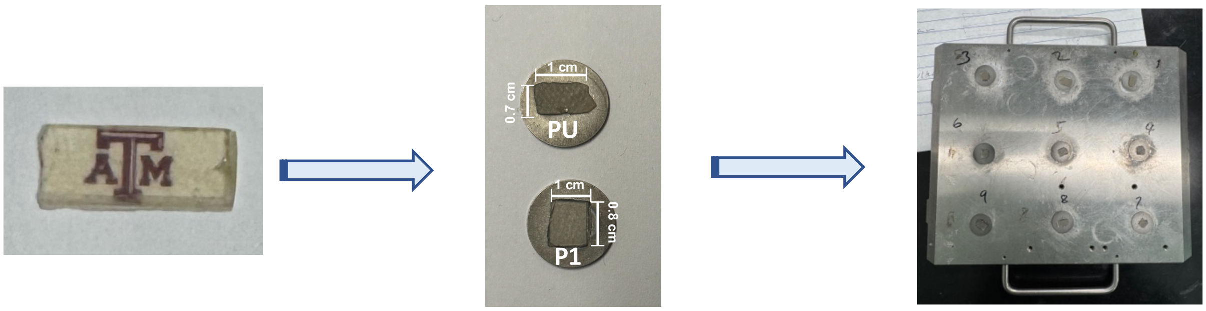

(d) **load-displacement curves for P1**

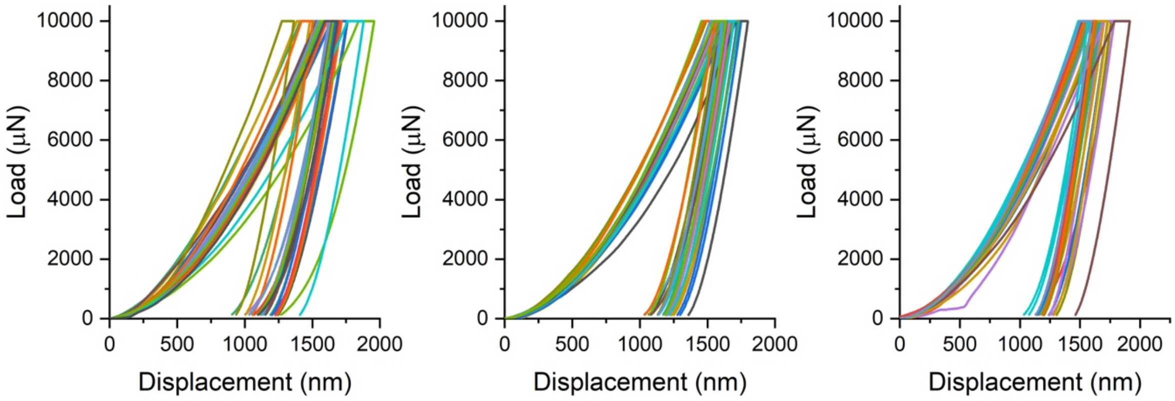

load-displacement curves for P5

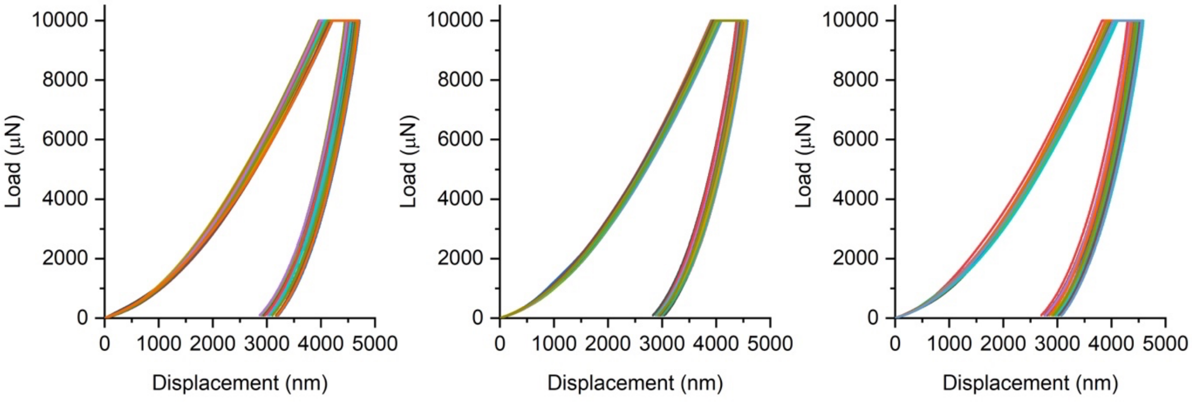

load-displacement curves for P10

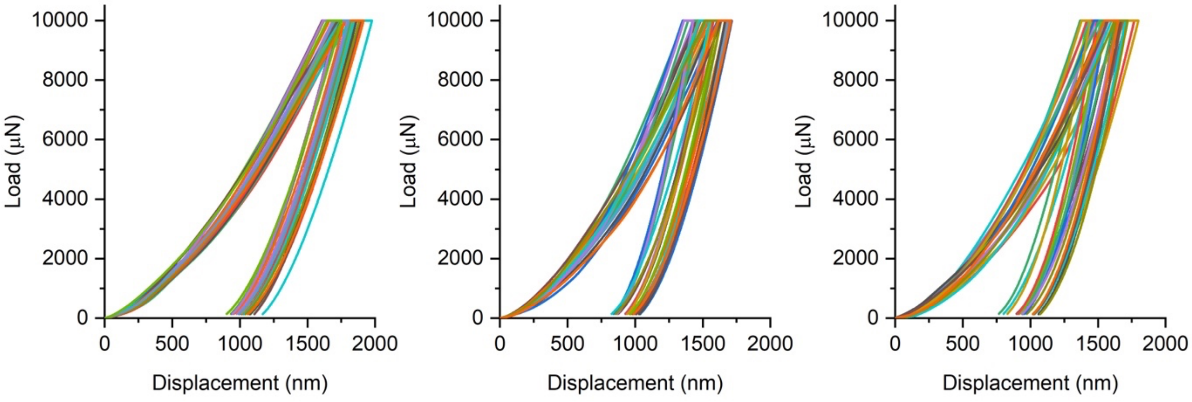

load-displacement curves for PU1

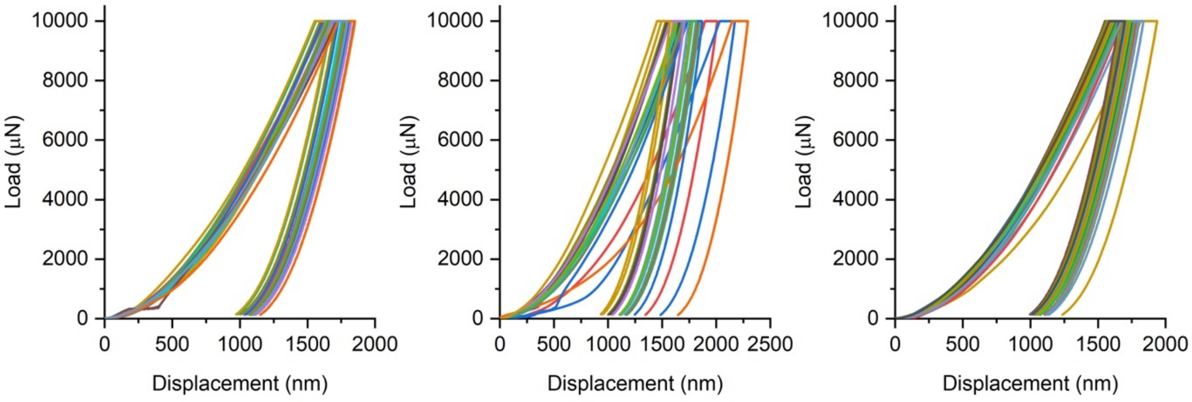

(e)

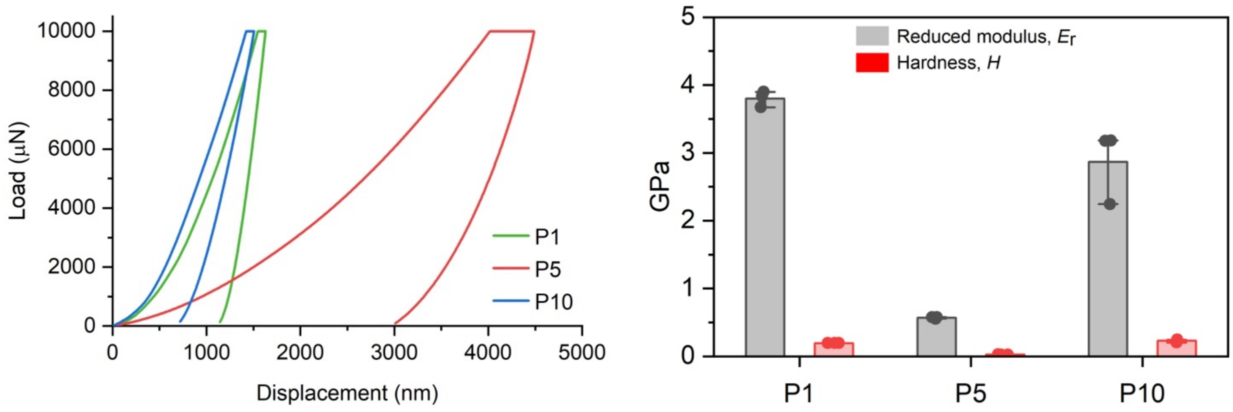

Table S3: Hardness (H) and Reduced Young's modulus (E_r) of polysulfamates P1, P5 and P10

Entry	M_w (kg/mol)	$Đ$	H (GPa)	E_r (GPa)
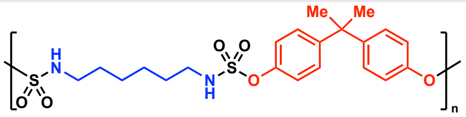 **P1**	23.4	2.04	0.20	3.82
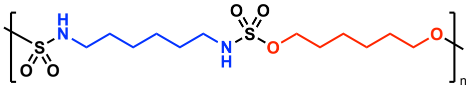 **P5**	8.7	2.30	0.026	0.570
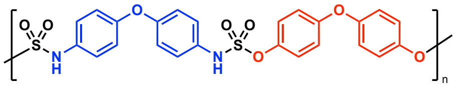 **P10**	17.7	2.30	0.23	2.88

(f)

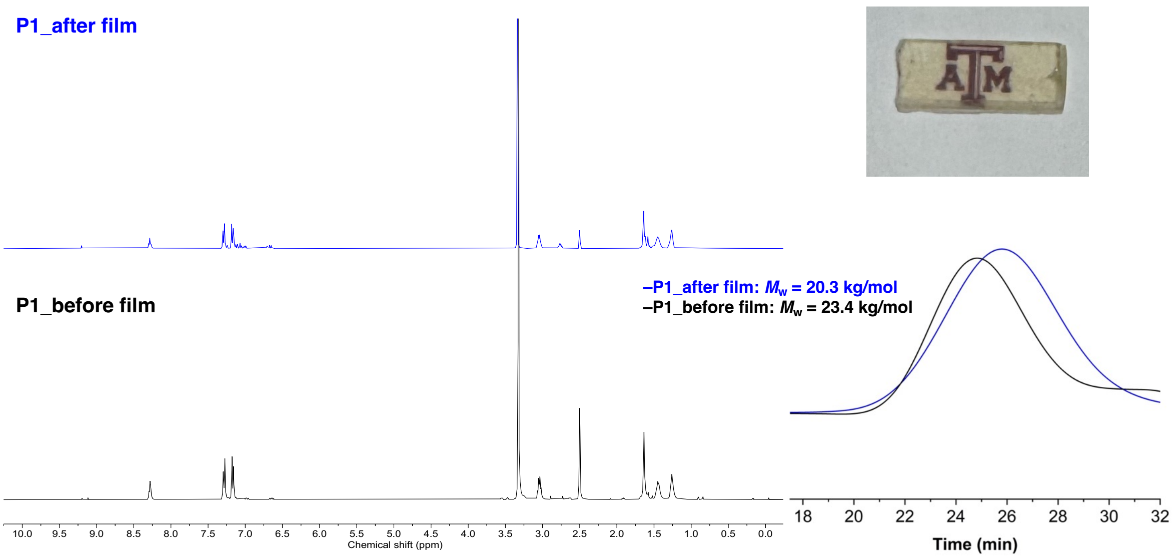

(g)

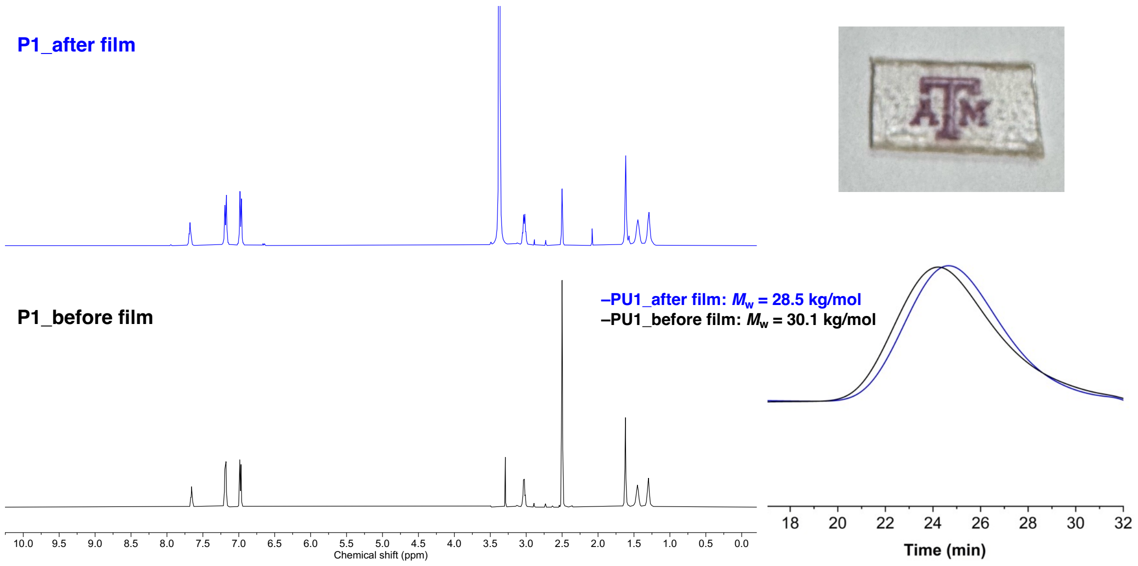

Figure S1: (a) polymer film fabricated utilizing hot press technique with a precisely dimensioned mold (4 cm × 3 cm × 0.6 mm); (b) nanoindentation sample prepared on steel AFM disc; (c) nanoindentation sample stage with magnetic stubs; (d) The load-displacement curves of **P1**, **P5**, **P10** and **PU1** each graph represents the average of ~25 indents from distinct polymer samples; (e) comparing load–displacement, reduced young's modulus (E_r) and hardness (H) of **P1**, **P5** and **P10**, (f) characterization of **P1** film, (g) characterization of **PU1** film.

3.7 Chemical degradation of polysulfamate and polyurethane via aqueous basic hydrolysis

i. Polysulfamate (P1)

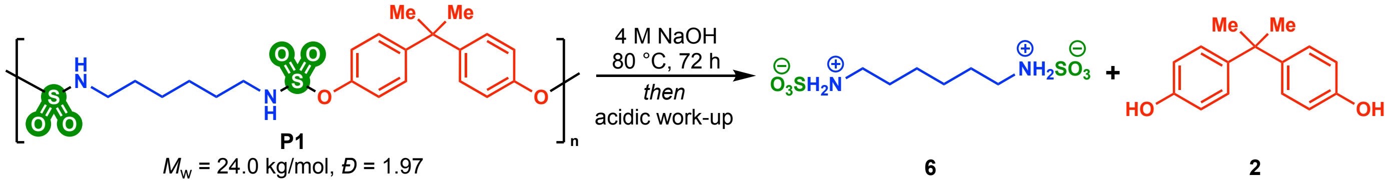

Table S4: Degradation reactions performed on polysulfamate P1

Entry	Aqueous solution [conc]	Temp. (°C)	Time (h)	Observation
		**Acidic aqueous solution**		
1	HCl [4 M]	rt	24	No degradation
2	HCl [4 M]	80	24	No degradation
3	HCl [4 M]	100	24	Multiple side products
4	H_2SO_4 [3 M]	60	24	No degradation
5	H_2SO_4 [6 M]	60	24	No degradation: additional peaks observed
		**Basic aqueous solution**		
6	NaOH [4 M]	rt	24	No degradation
7	NaOH [4 M]	60	24	**2** = 19%[a]
8	NaOH [4 M]	80	24	**2** = 62 %[a]
9	NaOH [4 M]	100	24	Multiple products
10	NaOH [4 M]	60	48	**2** = 50%[a]
11	NaOH [4 M]	80	48	**2** = 49%[a]
12	NaOH [4 M]	rt	1 week	No degradation
13	NaOH [4 M]	40	1 week	**2** = 14%[a]
14	NaOH [4 M]	50	1 week	**2** = 17%[a]
15	NaOH [4 M]	60	1 week	**2** = 63%[a]

[a] NMR yields using phenyltrimehylsilane as the internal standard.

Degradation of P1: General Procedure D

In a 50 mL round-bottom flask, **P1** (100 mg, 0.2 mmol) was suspended in 4 M aqueous NaOH (13.3 mL). The heterogeneous mixture was heated at 80 °C for 72 h under continuous stirring. Upon completion, the resulting transparent solution was cooled to room temperature and neutralized to pH ~7 using 4 M HCl. The formation of thick white precipitate was observed, which was removed by centrifugation. The

supernatant was then acidified to pH ~3 to ensure complete removal of excess salts, following which the resulting solution was extracted multiple times with EtOAc.

Recovery of Bisphenol A (2) from the EtOAc layer: The combined organic phases were dried over anhydrous Na_2SO_4 and evaporated completely. The resultant, yellow-colored residue was then dissolved in 5 mL hot toluene, filtered, and concentrated to yield **2**, as confirmed by ^1H NMR spectroscopy.

(*Note: The image of bisphenol A presented in Figure 4b was taken after recrystallization from toluene*)

Recovery of sulfamic acid (6) from the aqueous layer: The aqueous phase was evaporated and dried under vacuum for 2-3 h. The resulting white solids were suspended in anhydrous MeOH (15 mL) and sonicated for 2 min. The solution was then filtered and evaporated to yield hexane-1,6-diylbis(sulfamic acid), as confirmed by ^1H NMR and FT-IR spectroscopies.

(a)

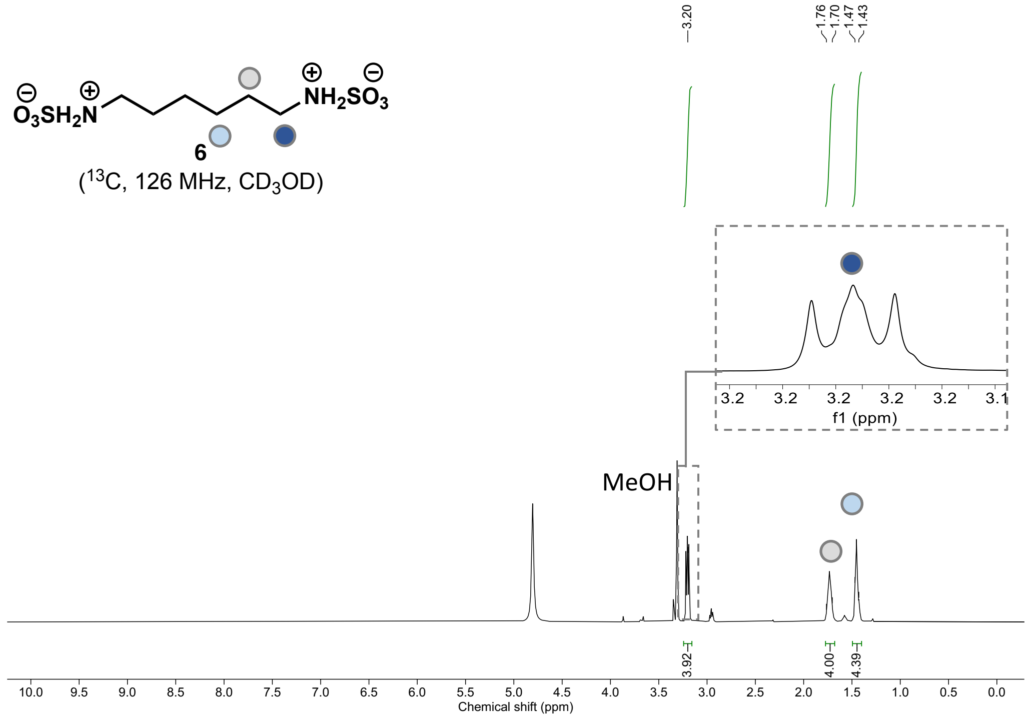

(b)

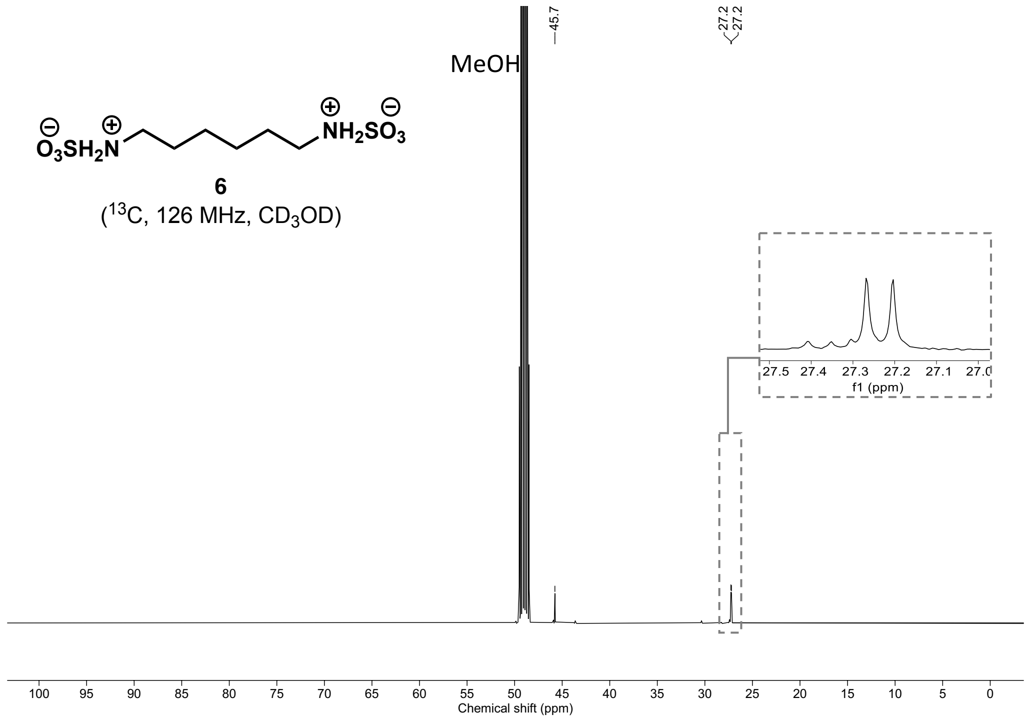

45.7
27.2
27.2
MeOH
6
(^{13}C, 126 MHz, CD_3OD)
27.5 27.4 27.3 27.2 27.1 27.0
f1 (ppm)
100 95 90 85 80 75 70 65 60 55 50 45 40 35 30 25 20 15 10 5 0
Chemical shift (ppm)

(c)

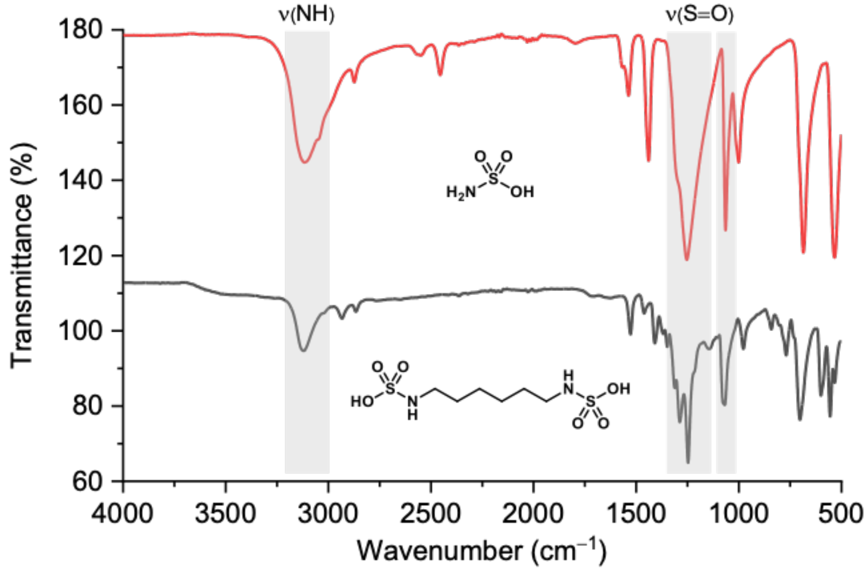

ν(NH)
ν(S=O)
Transmittance (%)
180
160
140
120
100
80
60
4000 3500 3000 2500 2000 1500 1000 500
Wavenumber (cm^{-1})

(d)

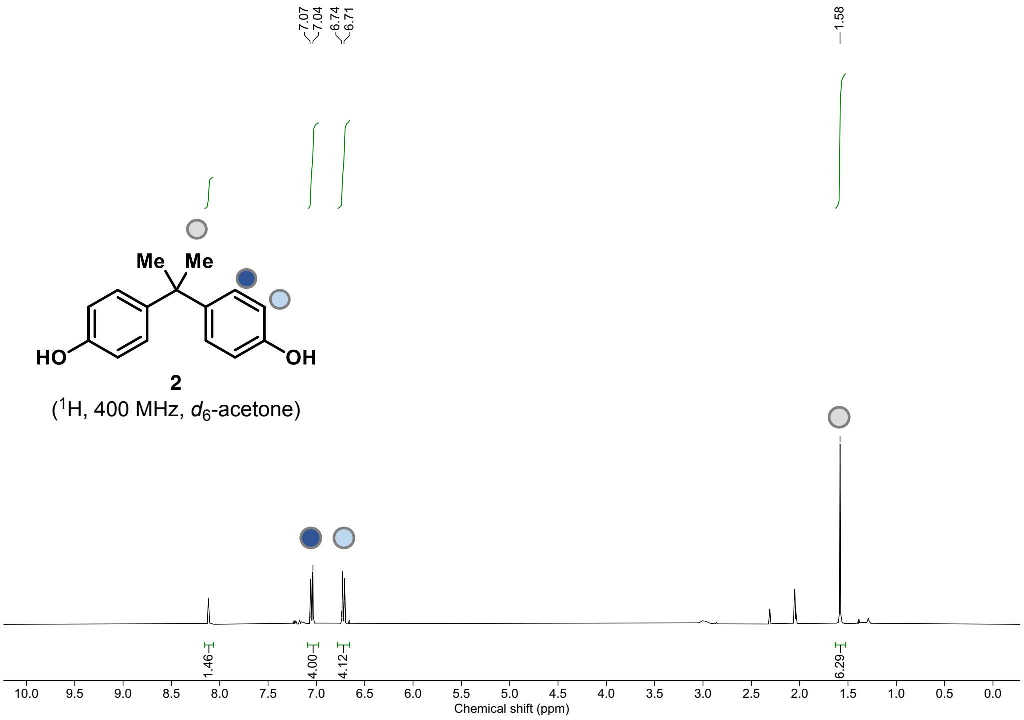

(e)

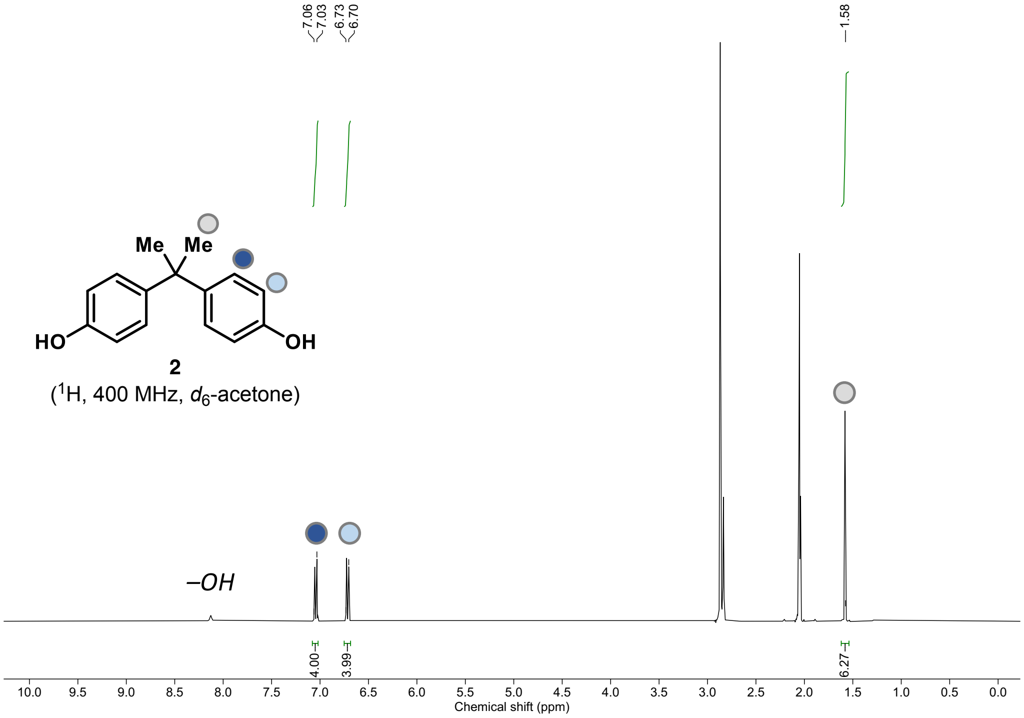

Figure S2. (a) ^{1}H-NMR spectra of **6** obtained after degradation of **P1**; (b) ^{13}C-NMR spectra of **6** obtained after degradation of **P1**; (c) FT-IR spectra of commercially available sulfamic acid stacked with the recovered bis(sulfamic acid) (**6**) obtained after degradation of **P1**; (d) ^{1}H-NMR spectra of **2** obtained after degradation of **P1**; (e) ^{1}H-NMR spectra of *crystallized* **2** obtained after degradation of **P1**.

ii. ***Polyurethane (PU1)***

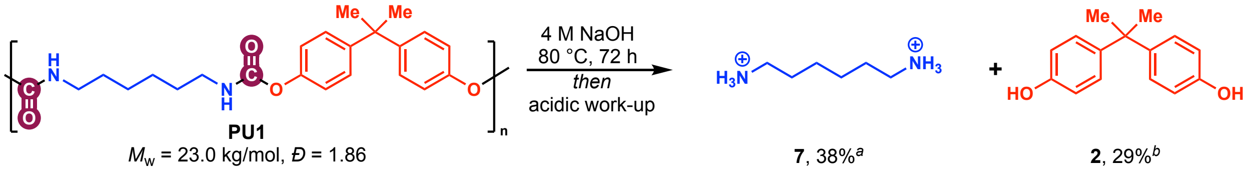

Polyurethane **PU1** was degraded and purified using ***general procedure D*** to allow for comparison of the efficiency of the process.

(a)

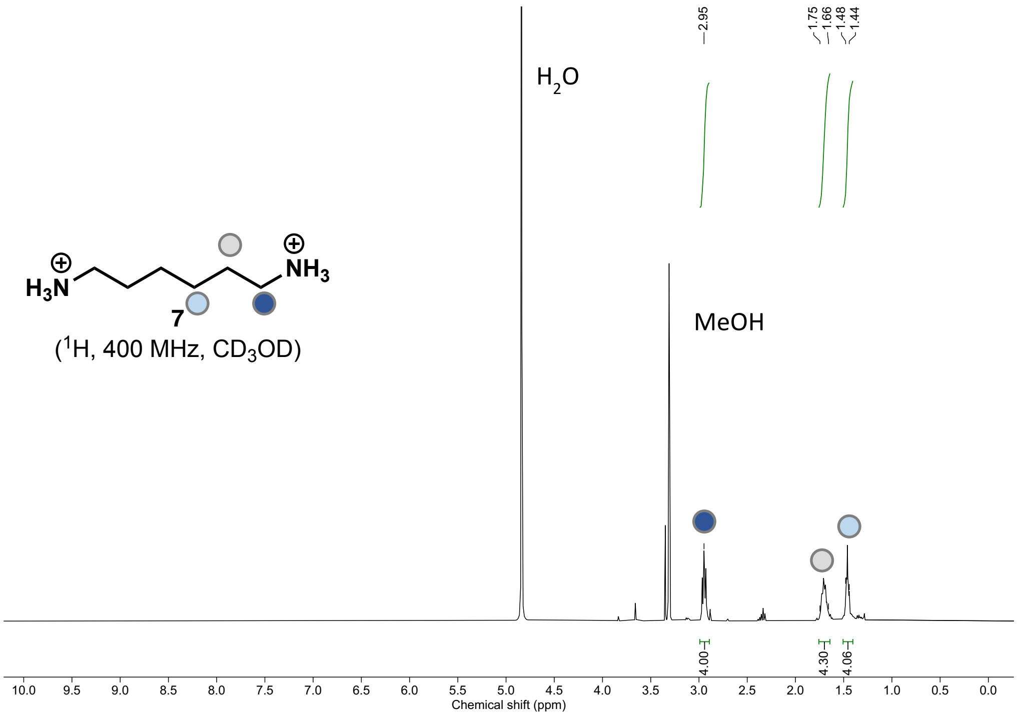

(b)

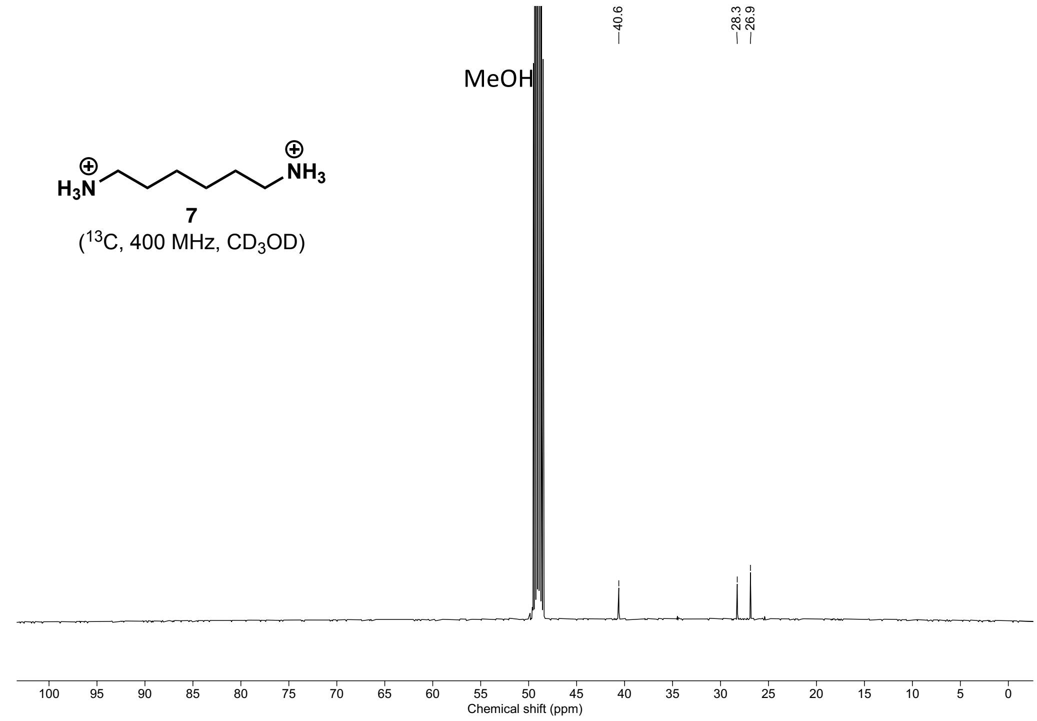

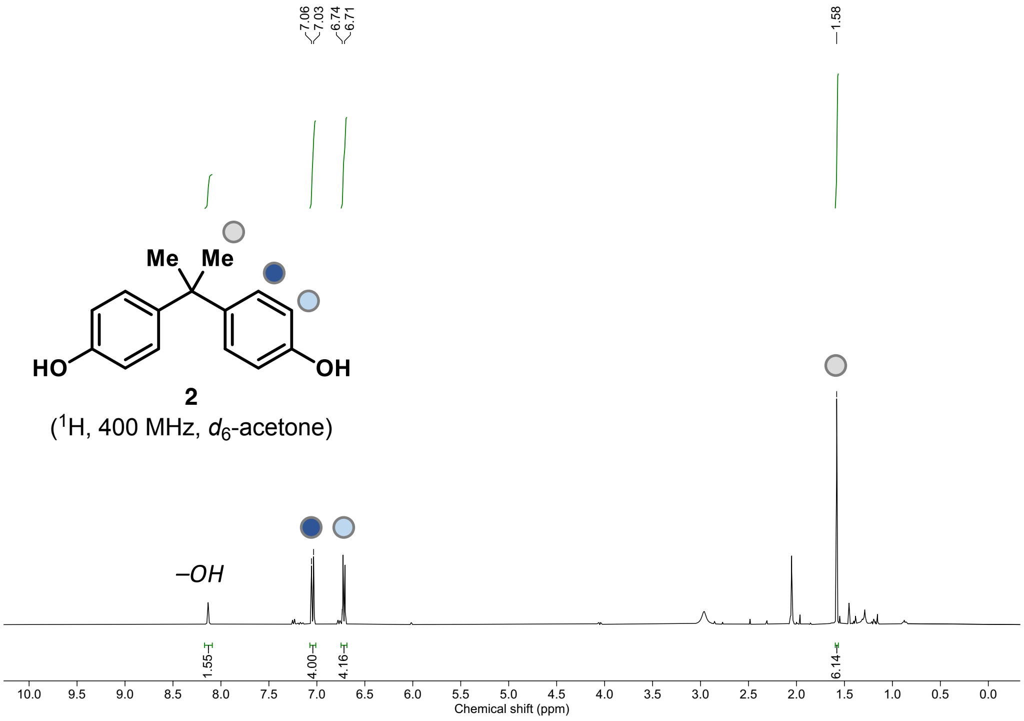

Figure S3. (a) ^{1}H-NMR spectra of diamine (**7**) obtained after degradation of **PU1**; (b) ^{13}C-NMR spectra of diamine (**7**) obtained after degradation of **PU1**; (c) ^{1}H-NMR spectra of **2** obtained after degradation of **PU1**.

iii. P1 vs PU1

Notably, under the same degradation conditions, **PU1** formed an opaque solution, contrary to the transparent solution observed for **P1**.

(a)

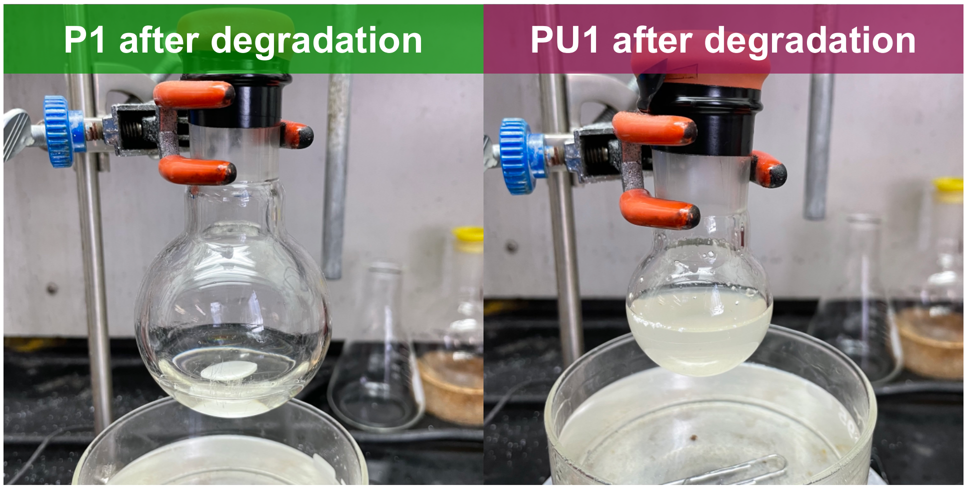

(b)

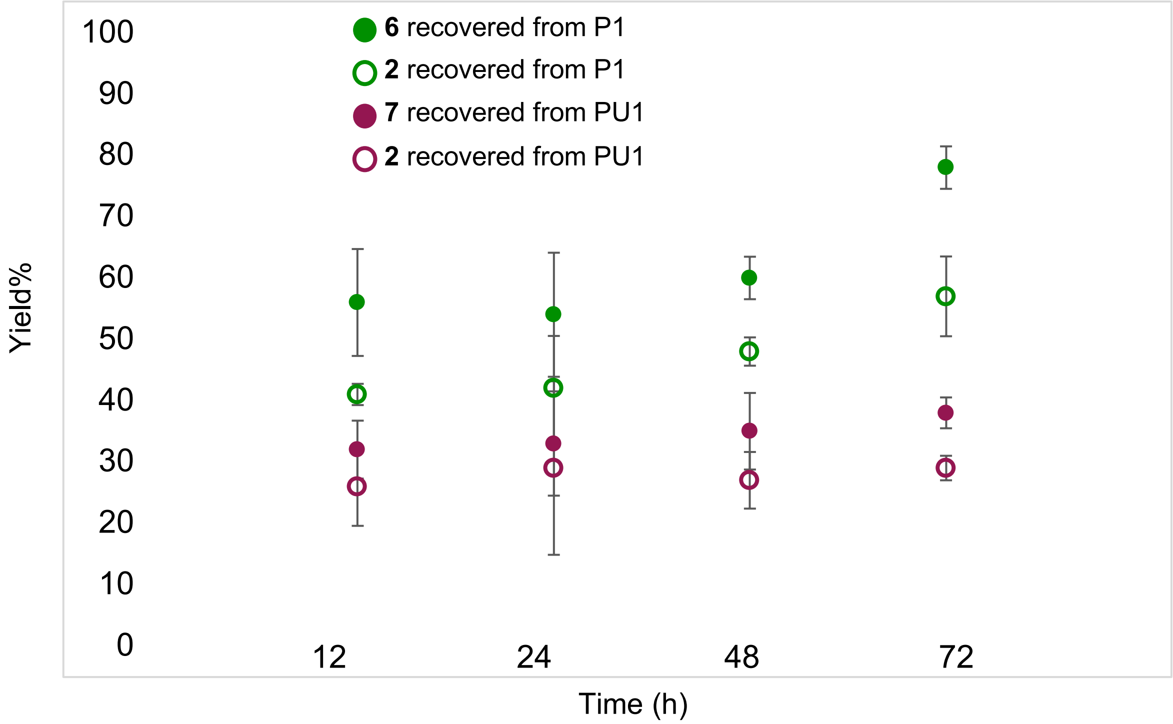

Figure S4. (a) **P1** *vs* **PU1** after 72 h of degradation; (b) Time-dependent monomer recovery from **P1** and **PU1**: graphical comparison.

iv. Degradation of soft–hard polysulfamates (P1, P2, P3 and P4)

The degradation for the following polysulfamates were conducted according to ***general procedure D***.

Table S5: Monomer recovery from the degradation of polysulfamates P1, P2,P3 and P4

Soft-hard polysulfamates	Structure of recovered diol with yield	Structure of recovered bis(sulfamic acid) with yield
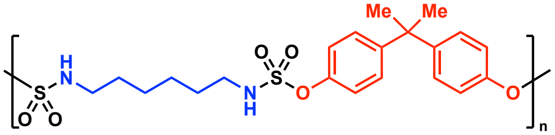 **P1**	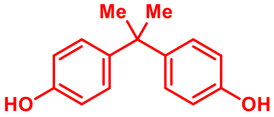 **57%**	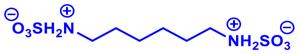 **74%**
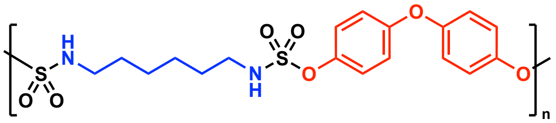 **P2**	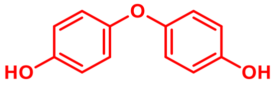 **87%**	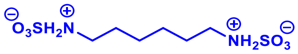 **39%**
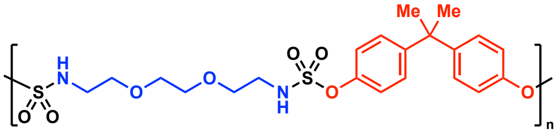 **P3**	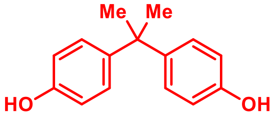 **73%**	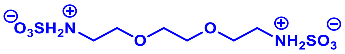 **46%**
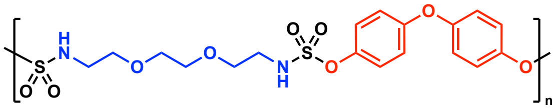 **P4**	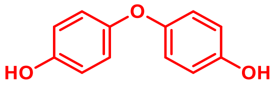 **76%**	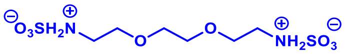 **47%**

NOTE: In the case of soft–soft (**P5–P8**) and hard–hard (**P9** and **P10**) polysulfamates, the polymers exhibited limited degradability, resulting in lower yields of the corresponding diols and sulfamic acids, with the optimized reaction condition.

4. Characterization of synthesized polymers

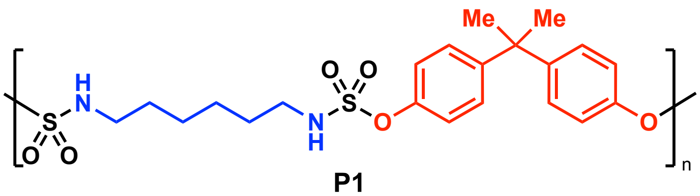

P1

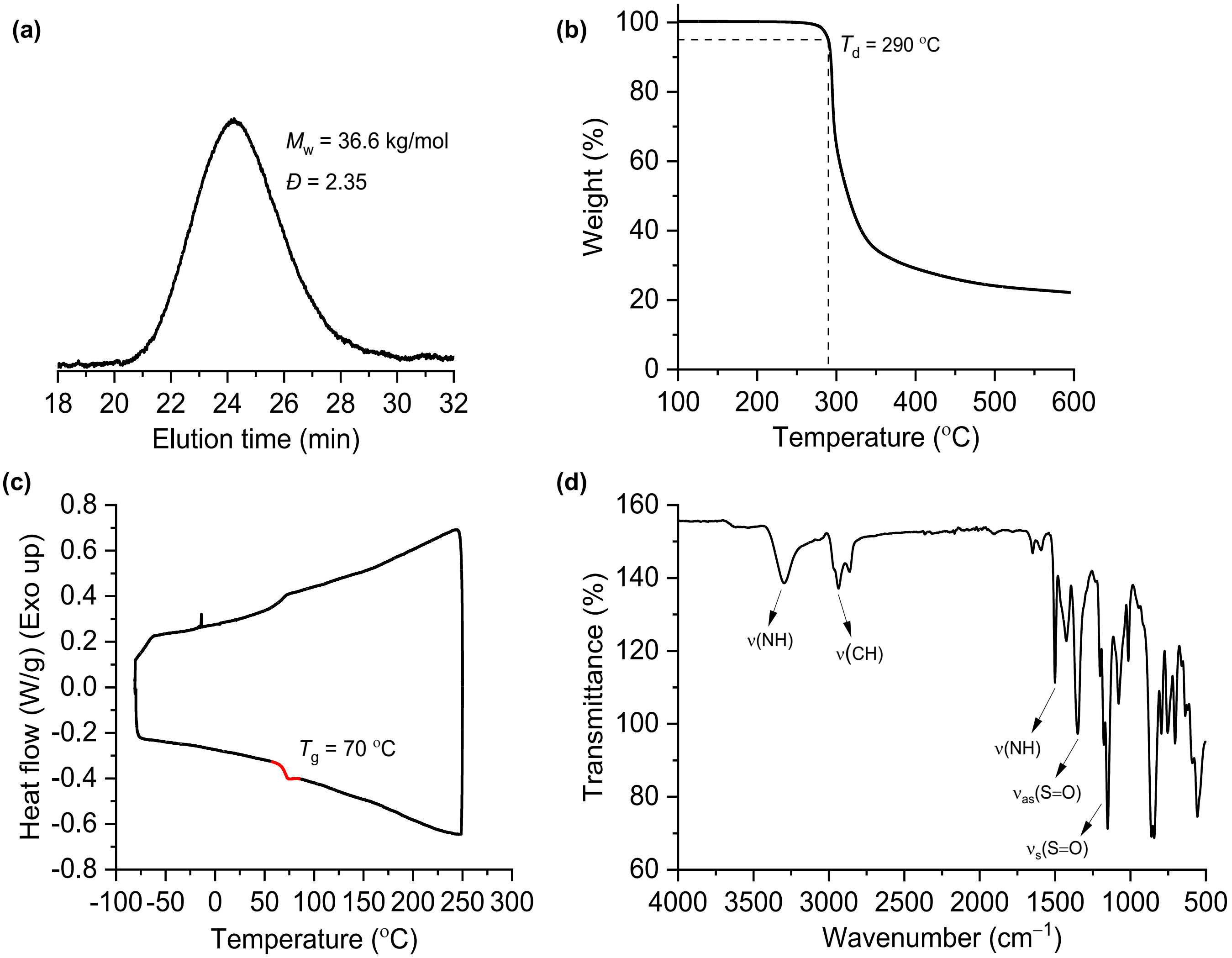

Figure S5. (a) SEC trace; (b) TGA thermogram; (c) DSC thermogram; (d) FT-IR spectrum.

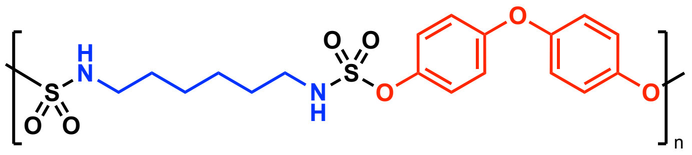

P2

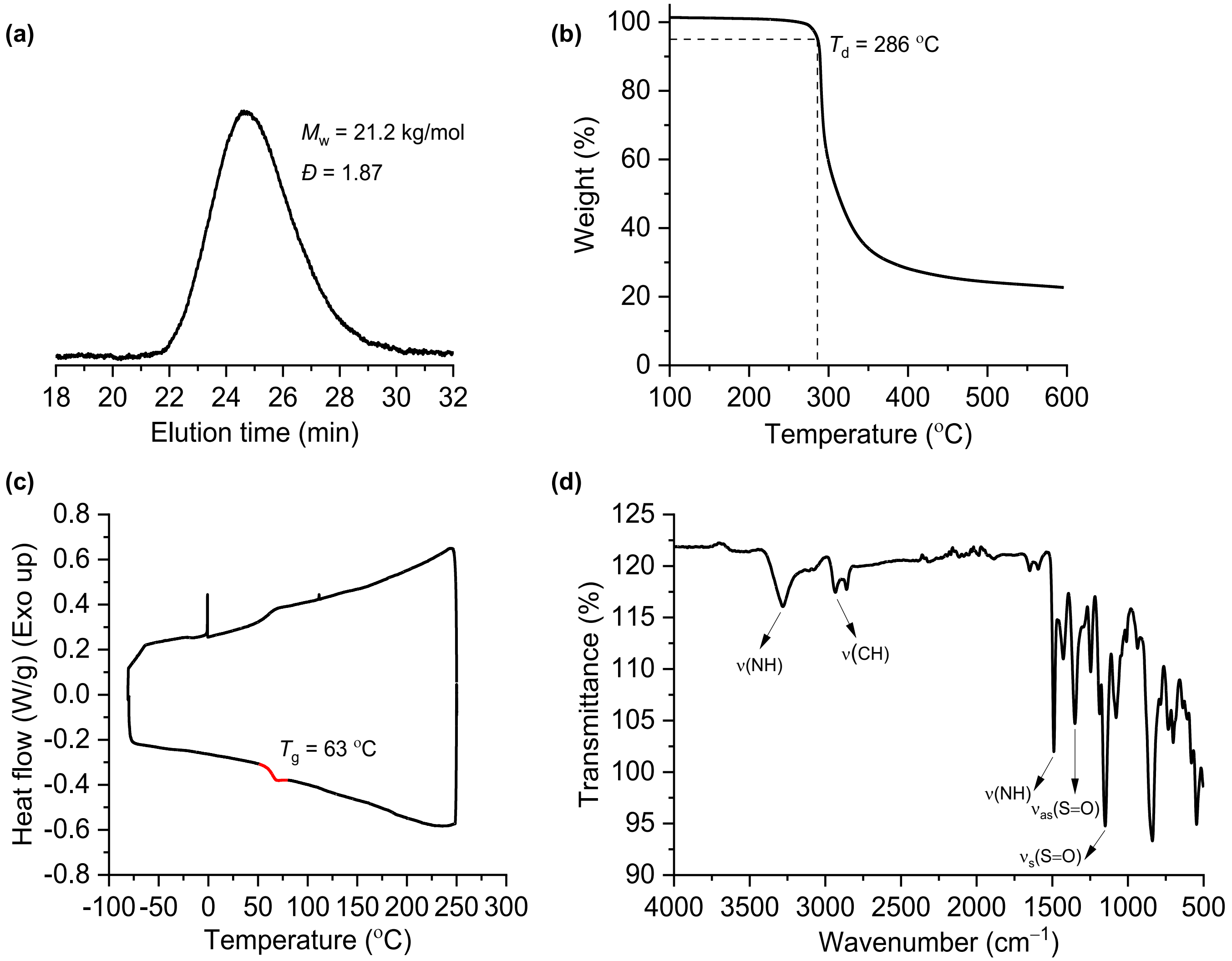

Figure S6. (a) SEC trace; (b) TGA thermogram; (c) DSC thermogram; (d) FT-IR spectrum.

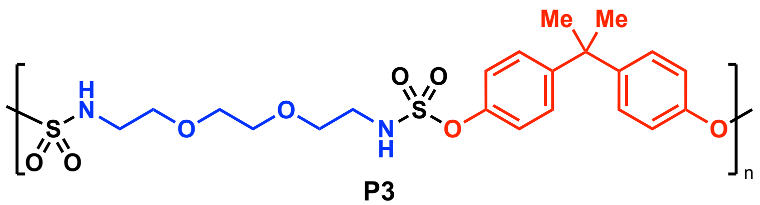

P3

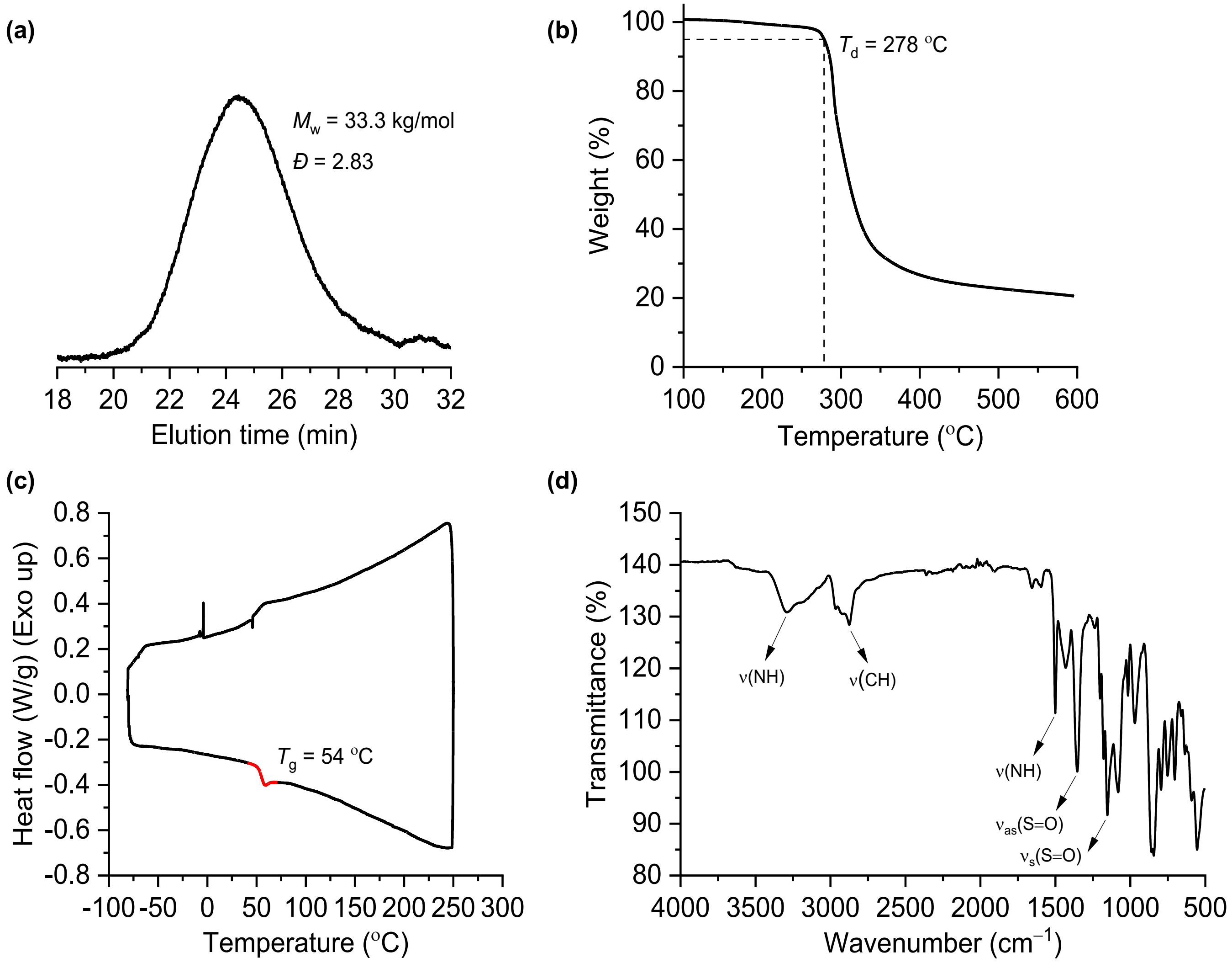

Figure S7. (a) SEC trace; (b) TGA thermogram; (c) DSC thermogram; (d) FT-IR spectrum.

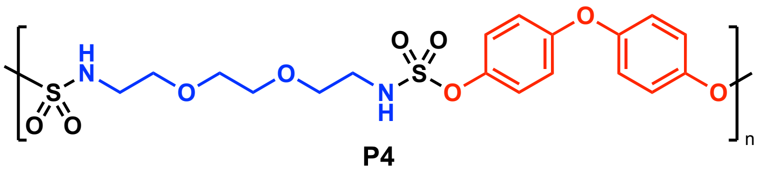

P4

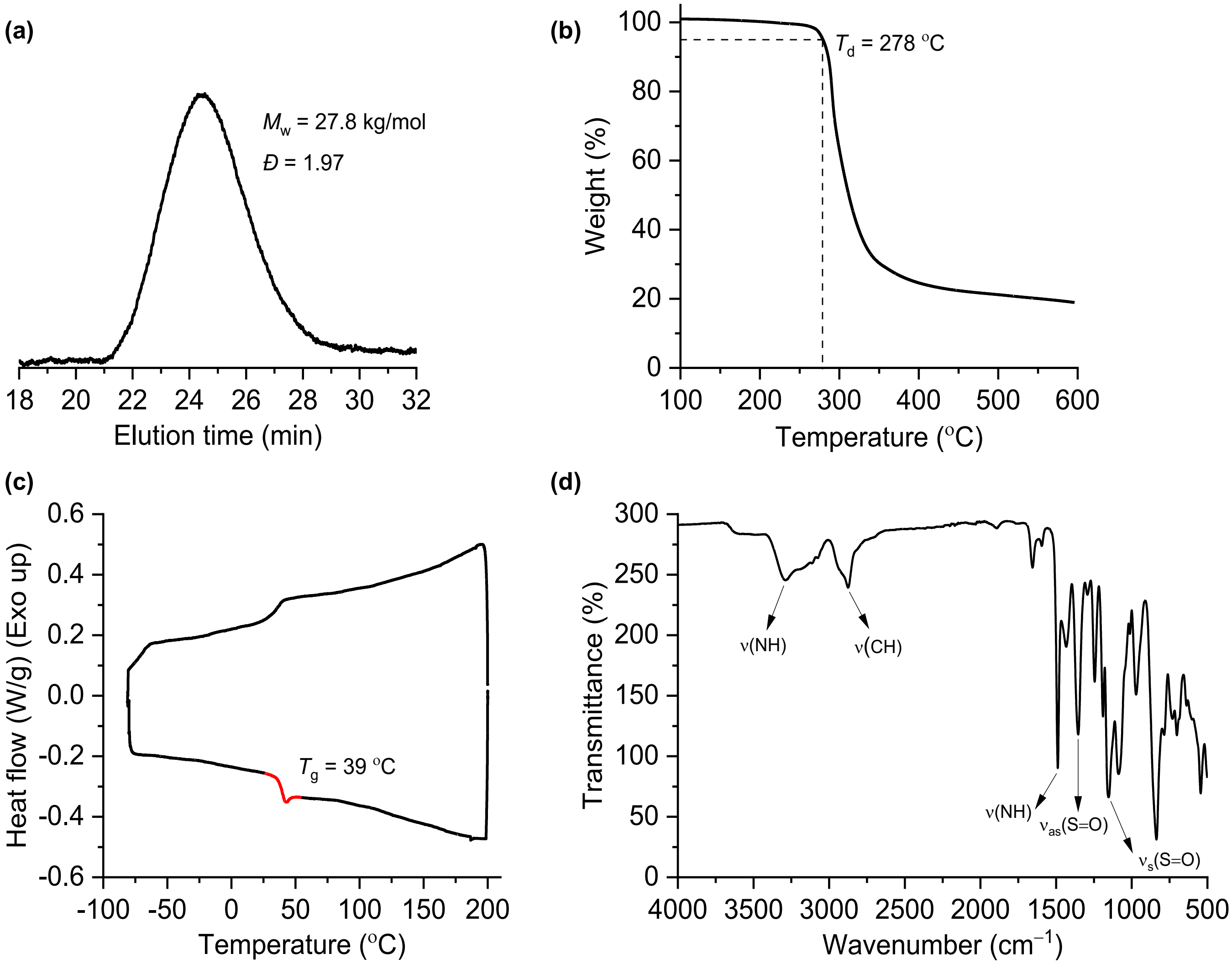

Figure S8. (a) SEC trace; (b) TGA thermogram; (c) DSC thermogram; (d) FT-IR spectrum.

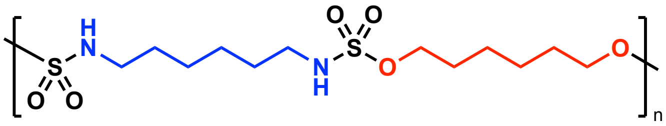

P5

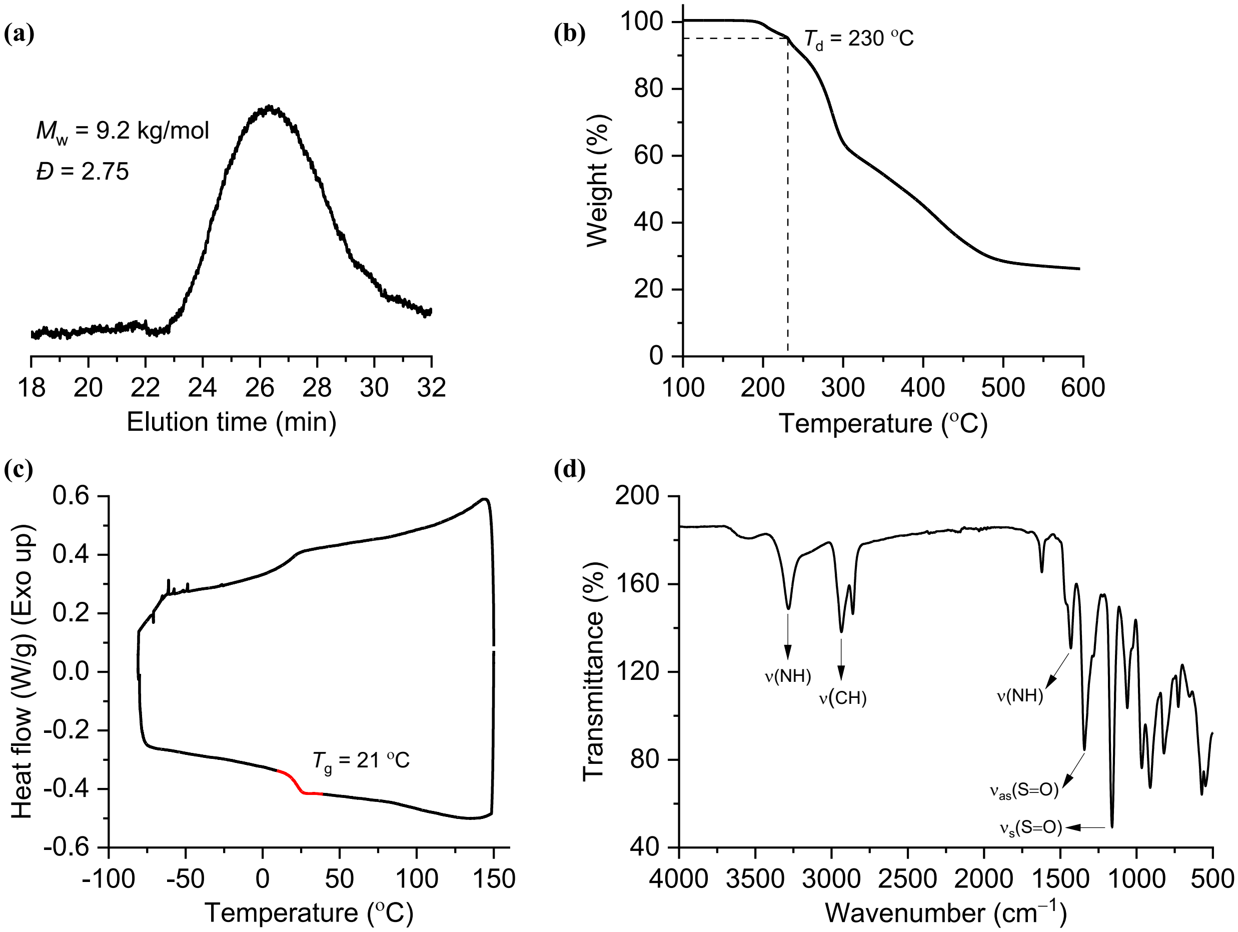

Figure S9. (a) SEC trace; (b) TGA thermogram; (c) DSC thermogram; (d) FT-IR spectrum.

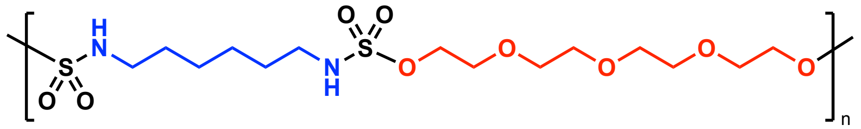

P6

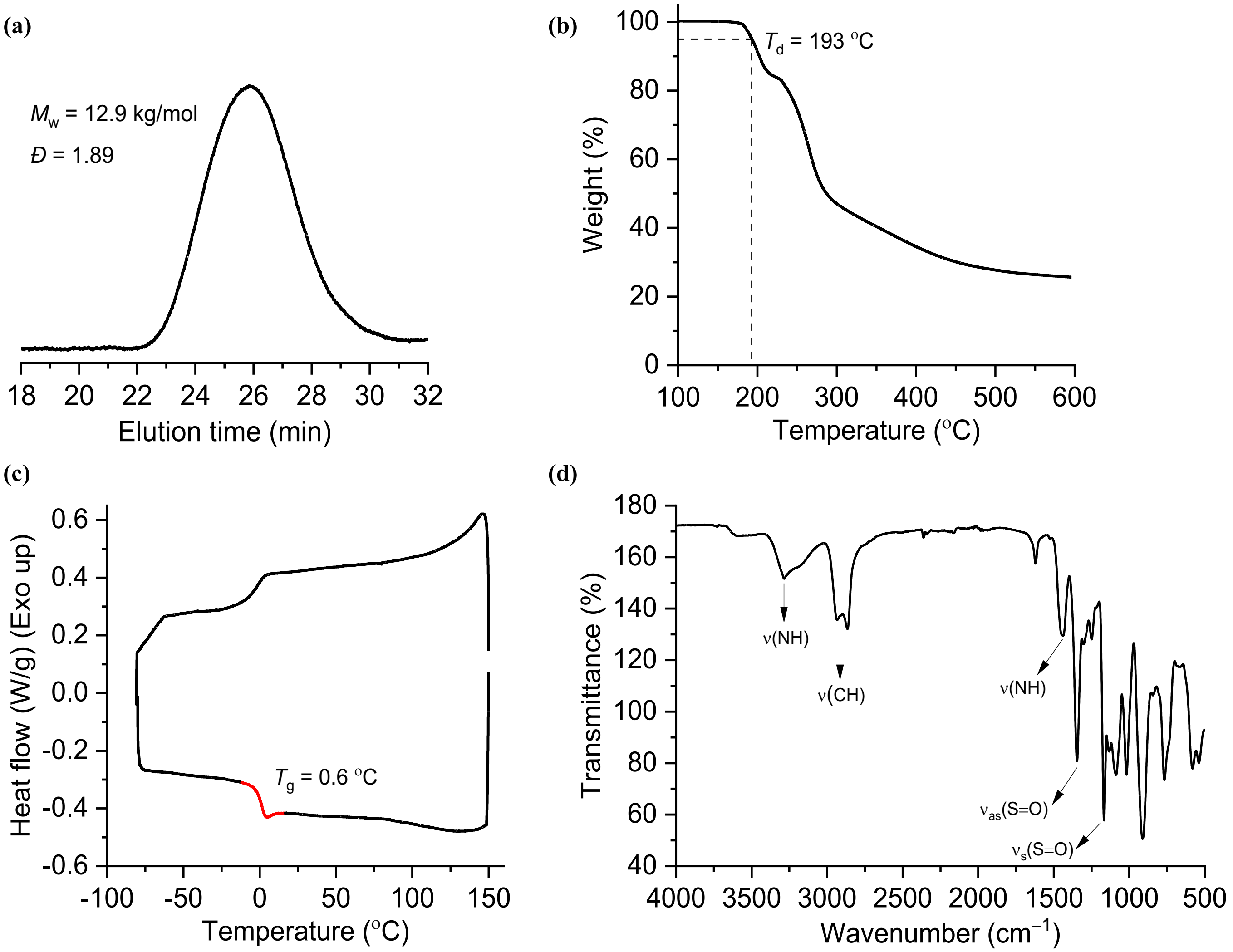

Figure S10. (a) SEC trace; (b) TGA thermogram; (c) DSC thermogram; (d) FT-IR spectrum.

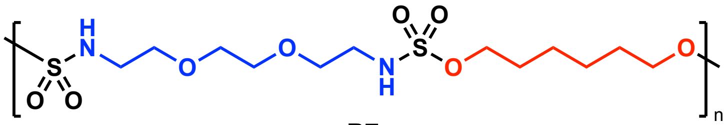

P7

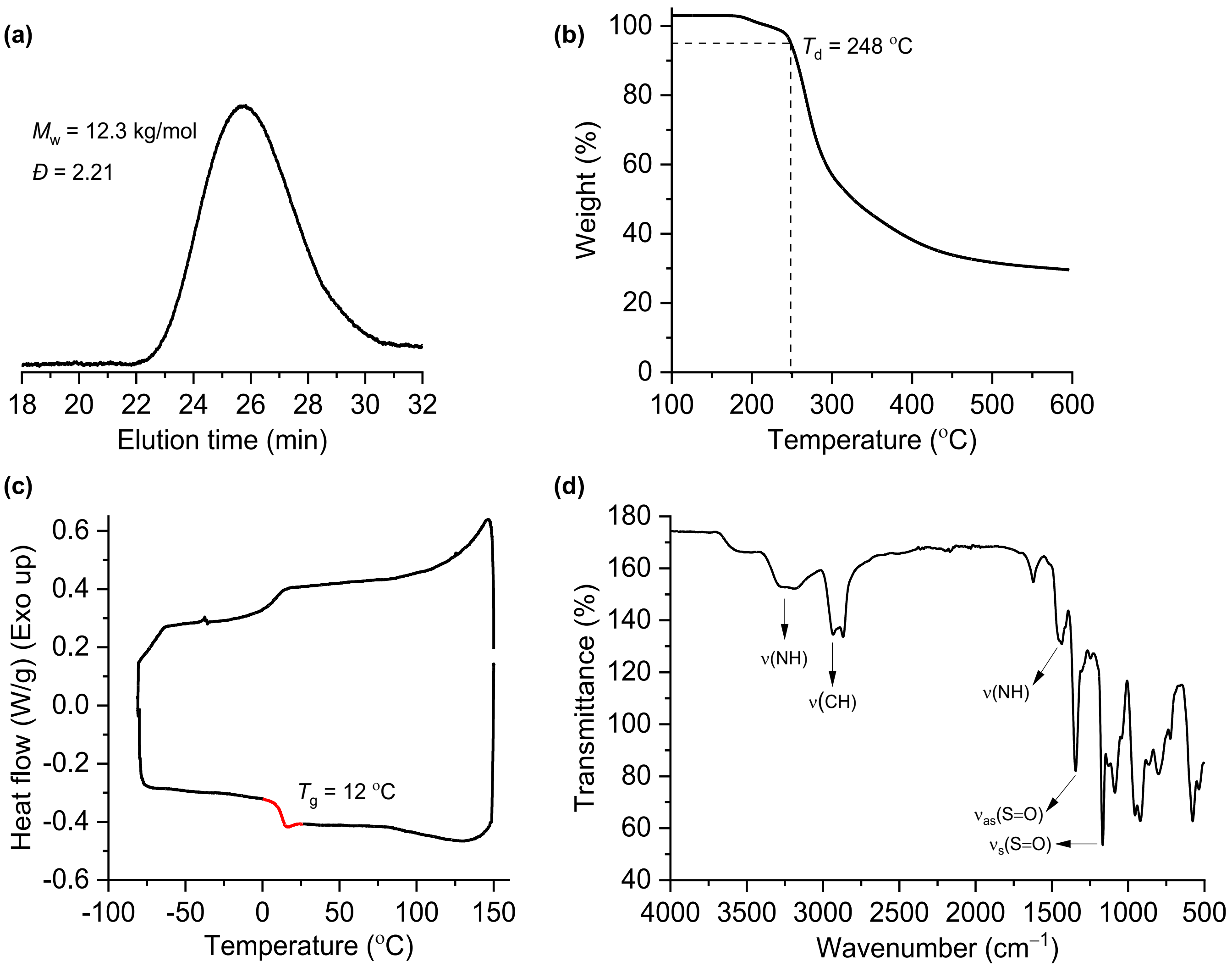

Figure S11. (a) SEC trace; (b) TGA thermogram; (c) DSC thermogram; (d) FT-IR spectrum.

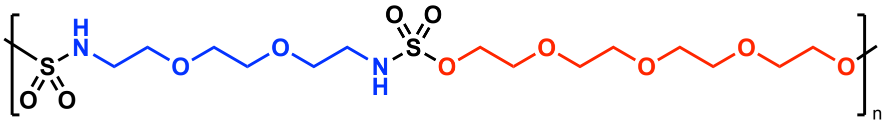

P8

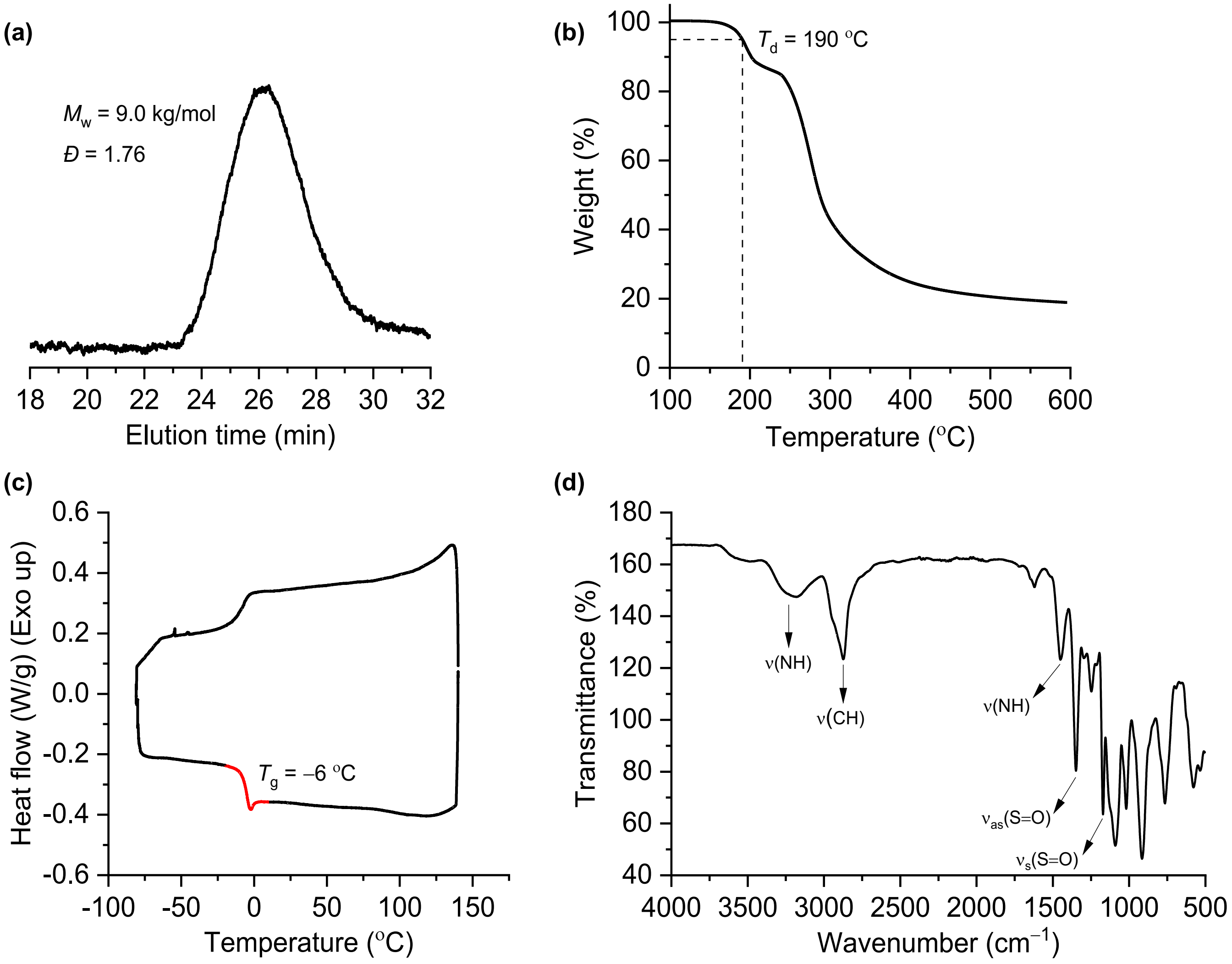

Figure S12. (a) SEC trace; (b) TGA thermogram; (c) DSC thermogram; (d) FT-IR spectrum.

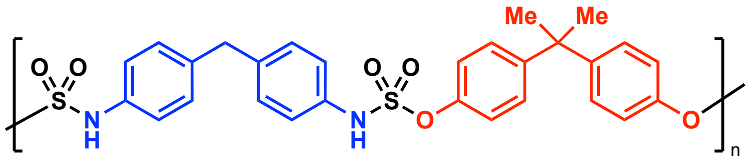

P9

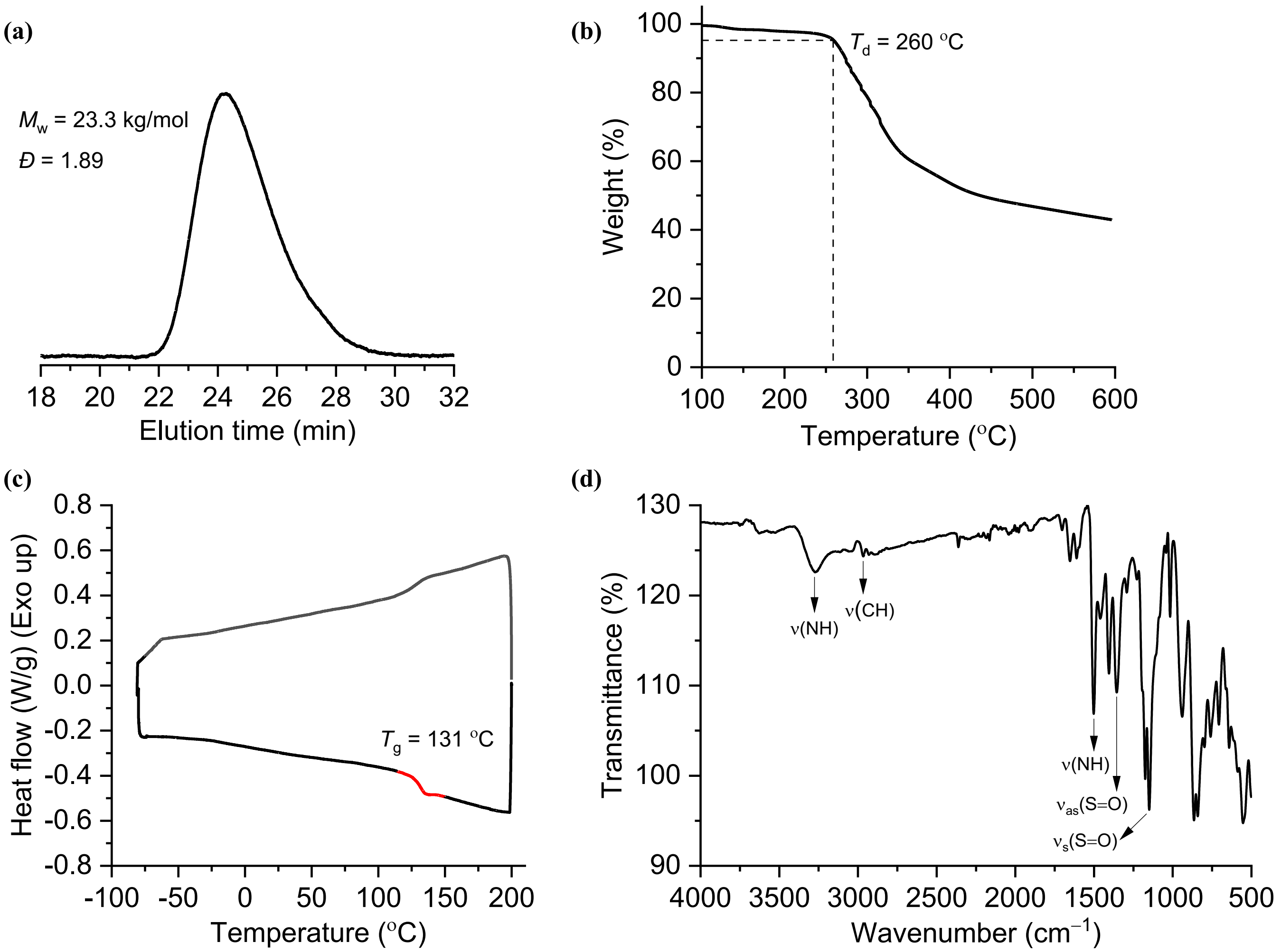

Figure S13. (a) SEC trace; (b) TGA thermogram; (c) DSC thermogram; (d) FT-IR spectrum.

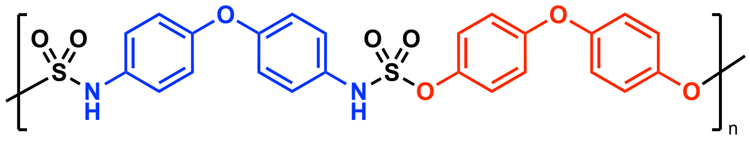

P10

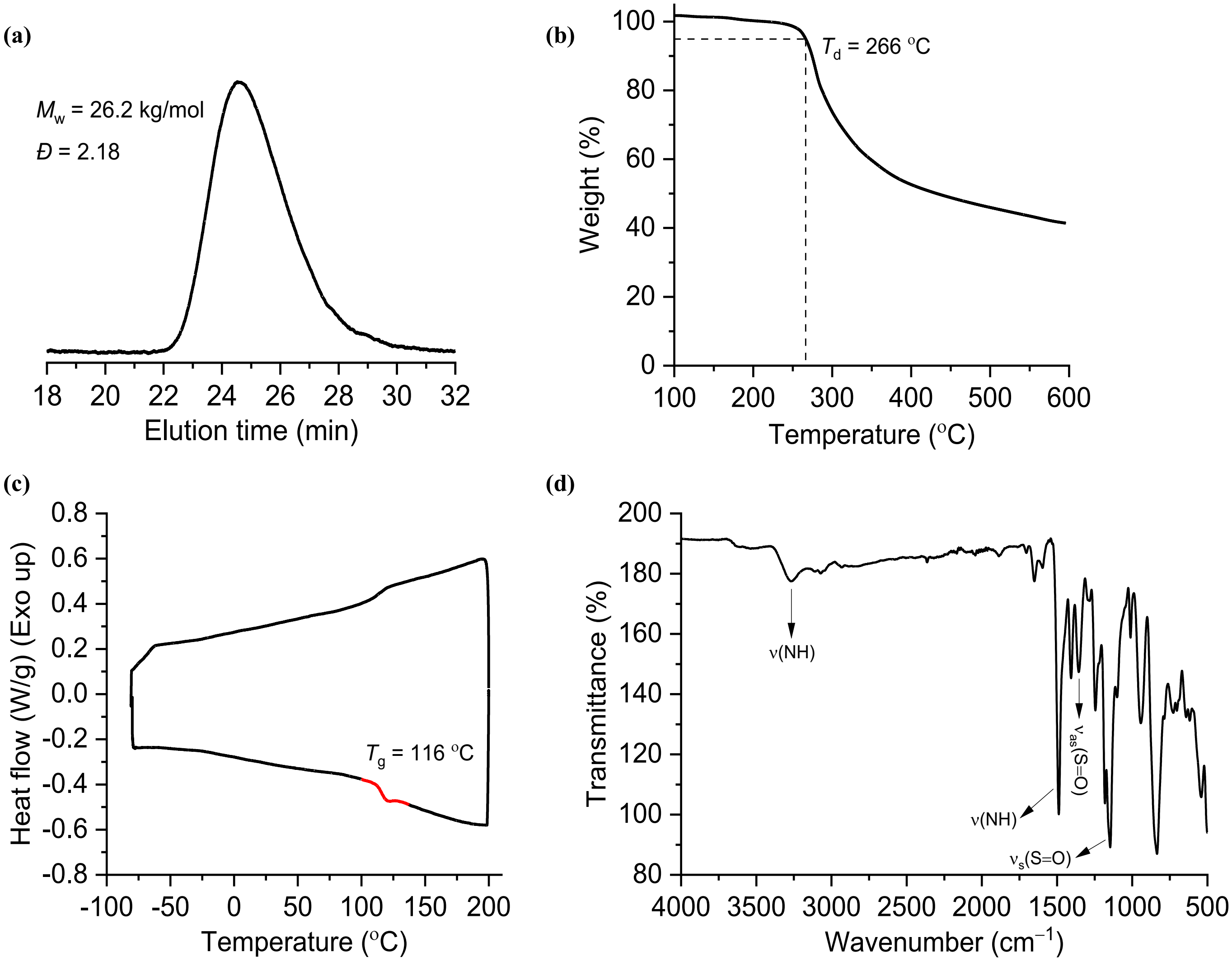

Figure S14. (a) SEC trace; (b) TGA thermogram; (c) DSC thermogram; (d) FT-IR spectrum.

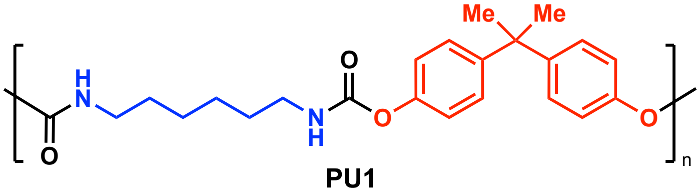

PU1

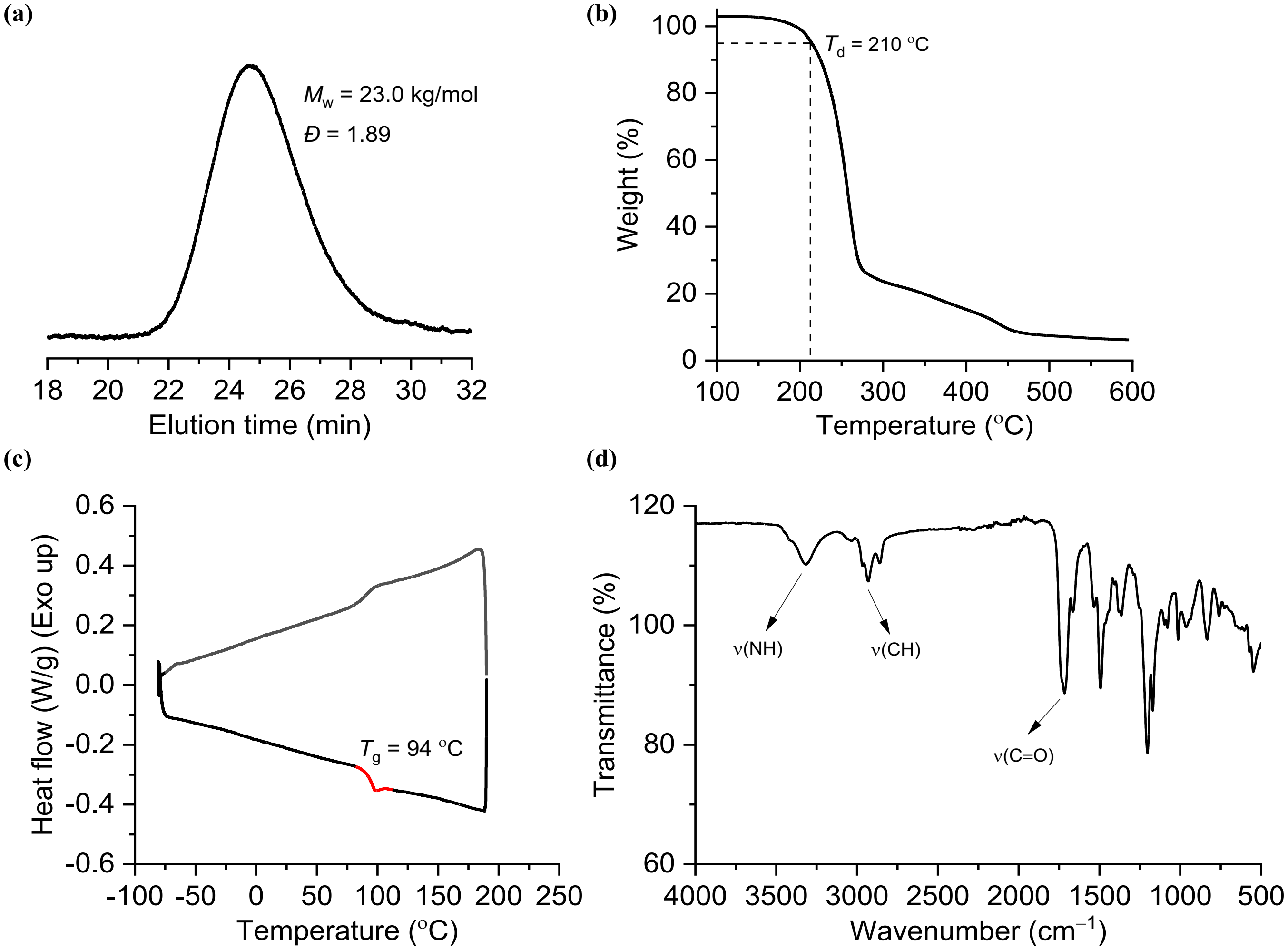

Figure S15. (a) SEC trace; (b) TGA thermogram; (c) DSC thermogram; (d) FT-IR spectrum.

Powder XRD analysis

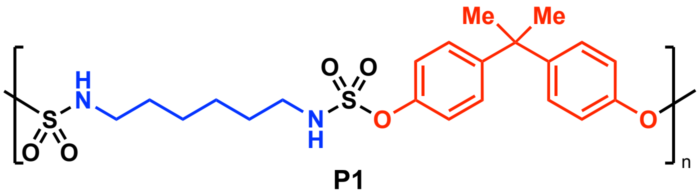

P1

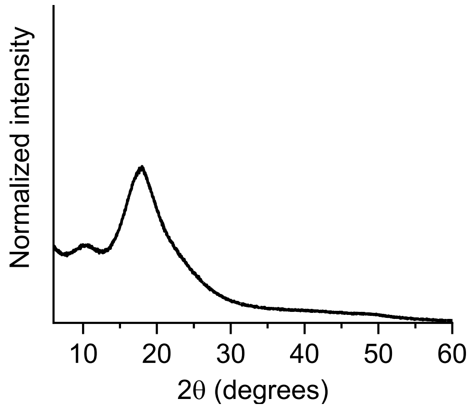

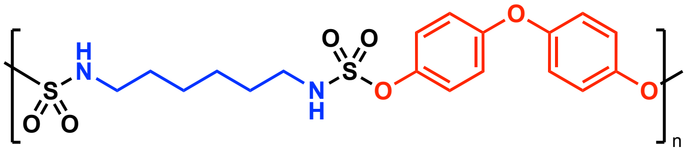

P2

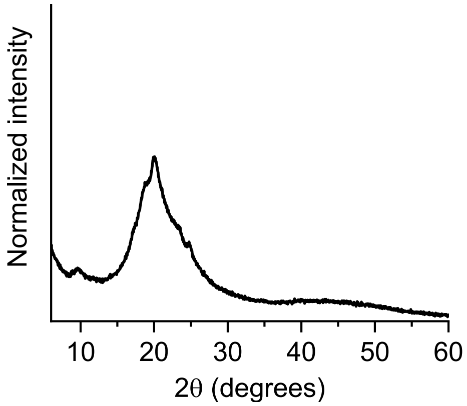

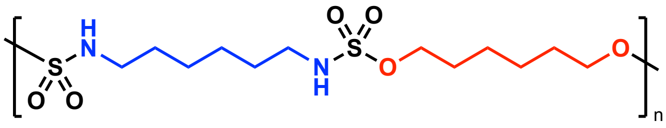

P5

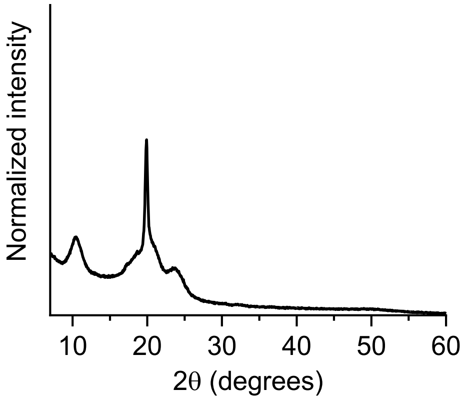

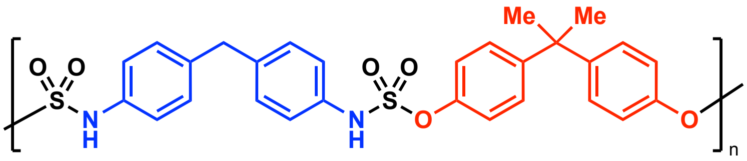

P9

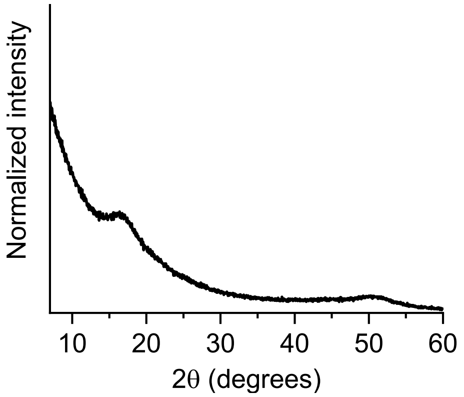

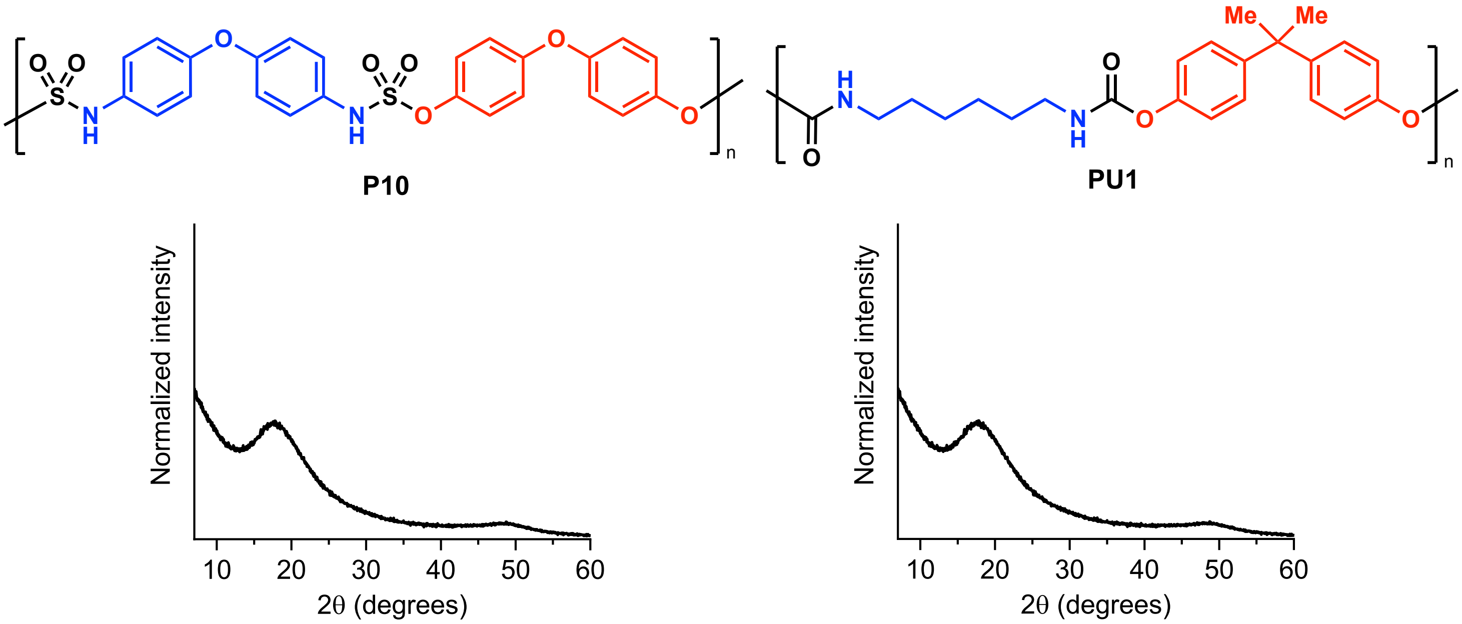

Figure S16. Powder XRD pattern of polysulfamates **P1**, **P2**, **P5**, **P9**, **P10** and polyurethane **PU1**.

5. NMR spectra of synthesized monomers and polymers

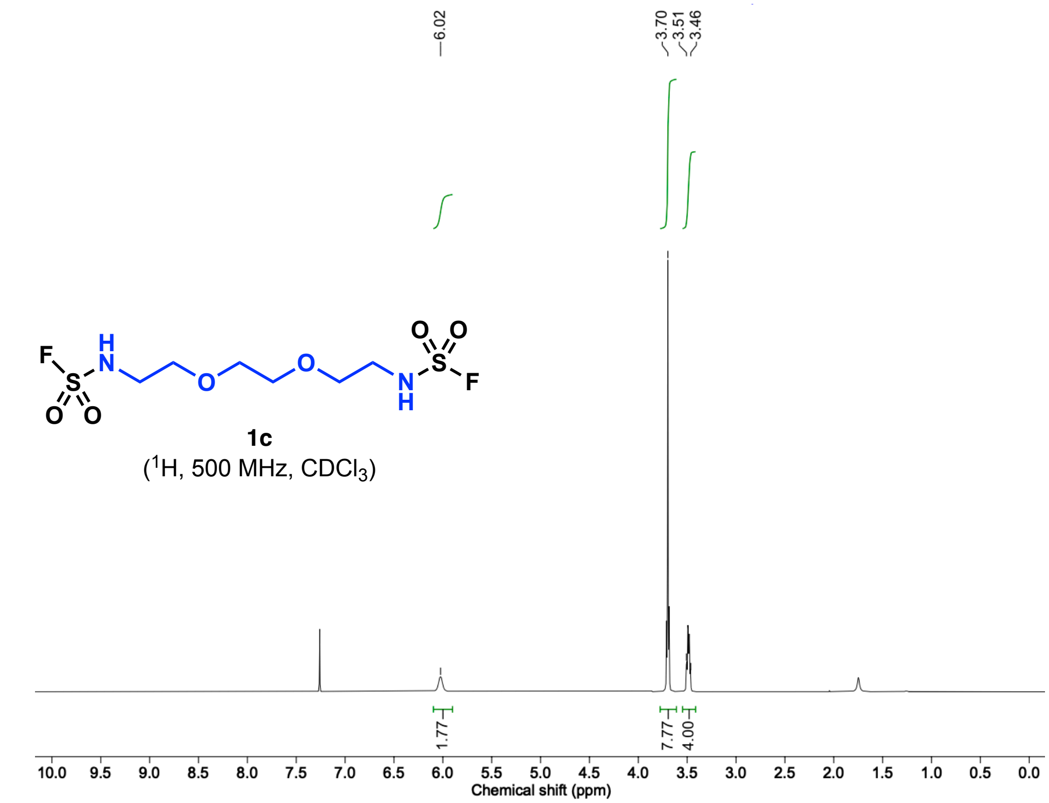

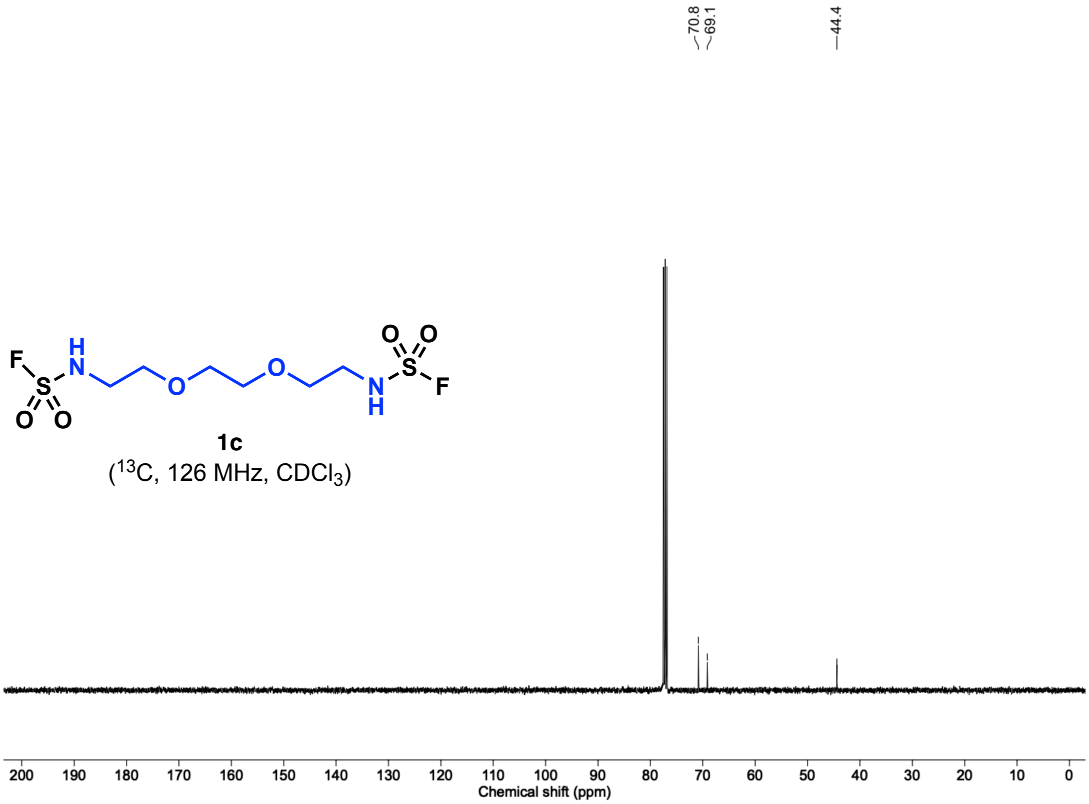

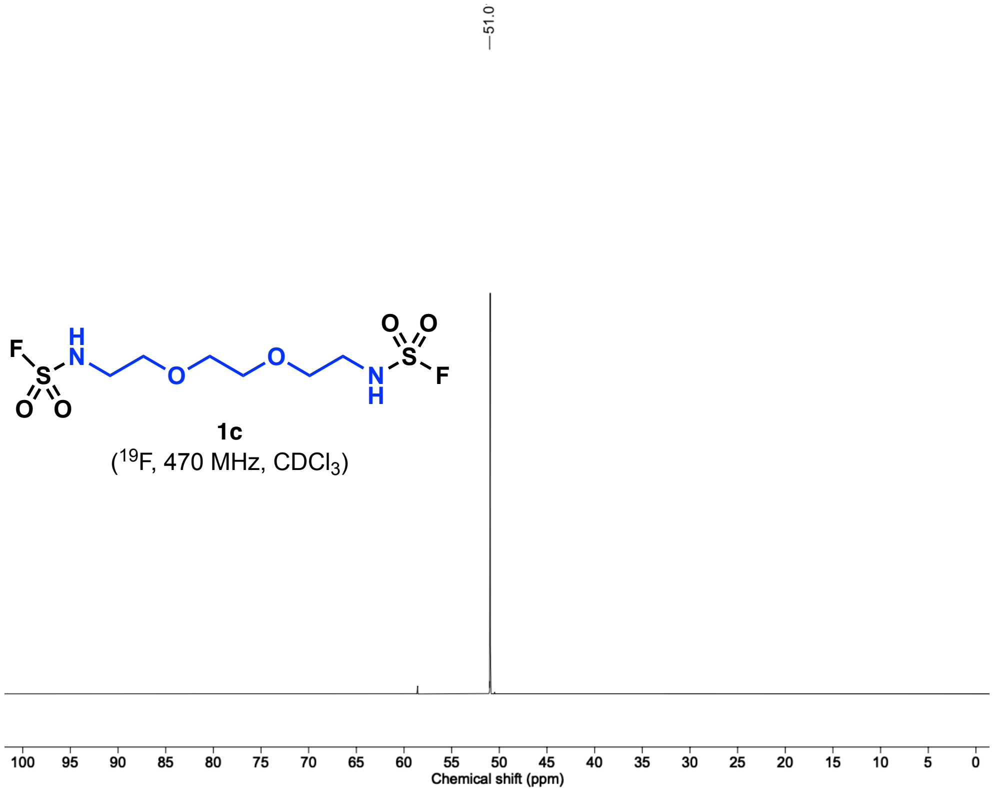

1c
(19F, 470 MHz, CDCl3)
—51.0
100 95 90 85 80 75 70 65 60 55 50 45 40 35 30 25 20 15 10 5 0
Chemical shift (ppm)

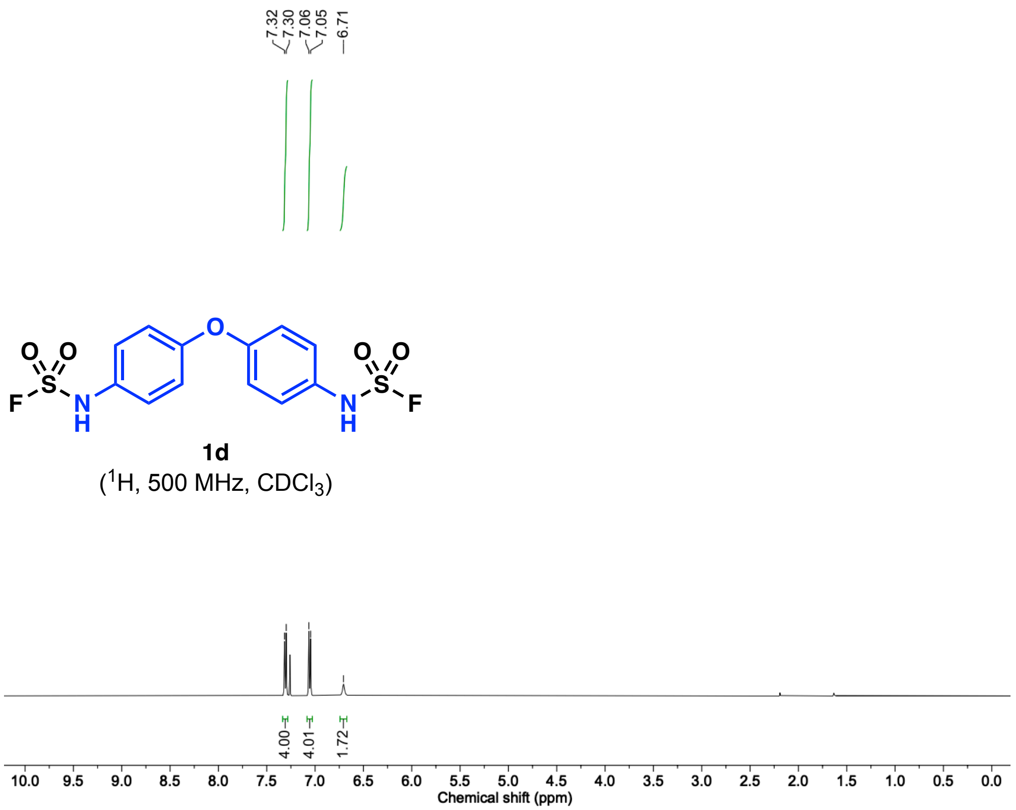

1d

(^{1}H, 500 MHz, $CDCl_3$)

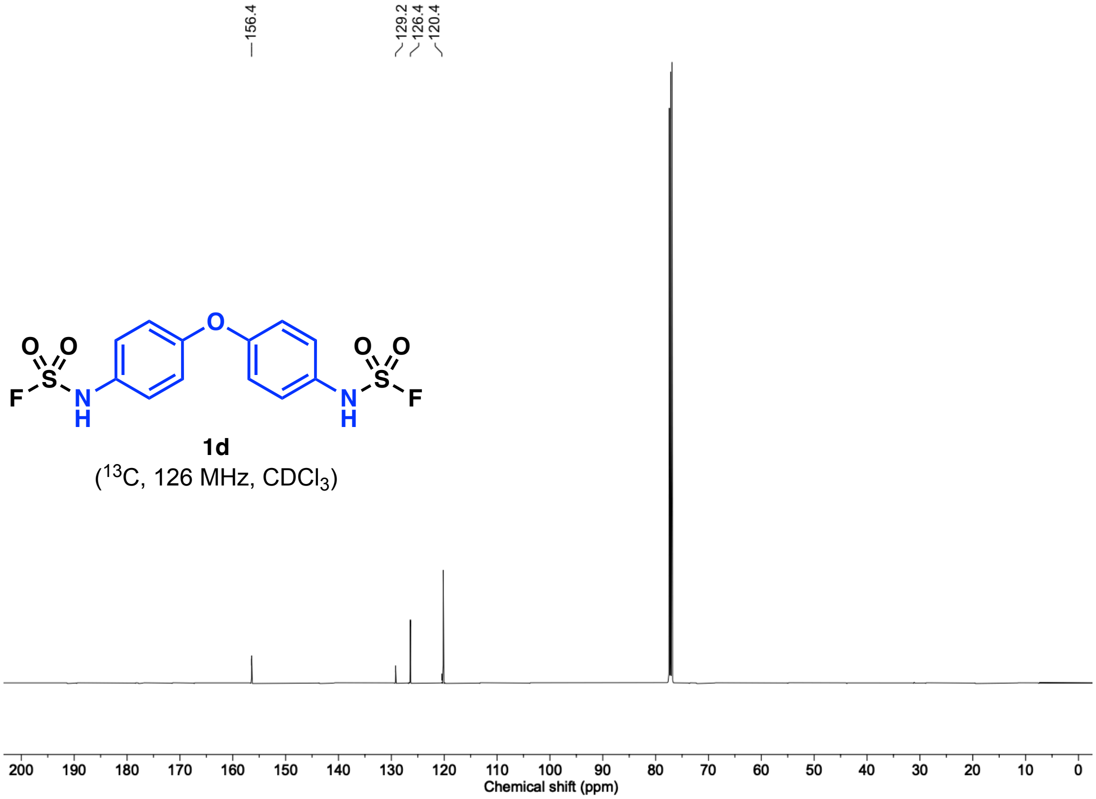

1d

(^{13}C, 126 MHz, $CDCl_3$)

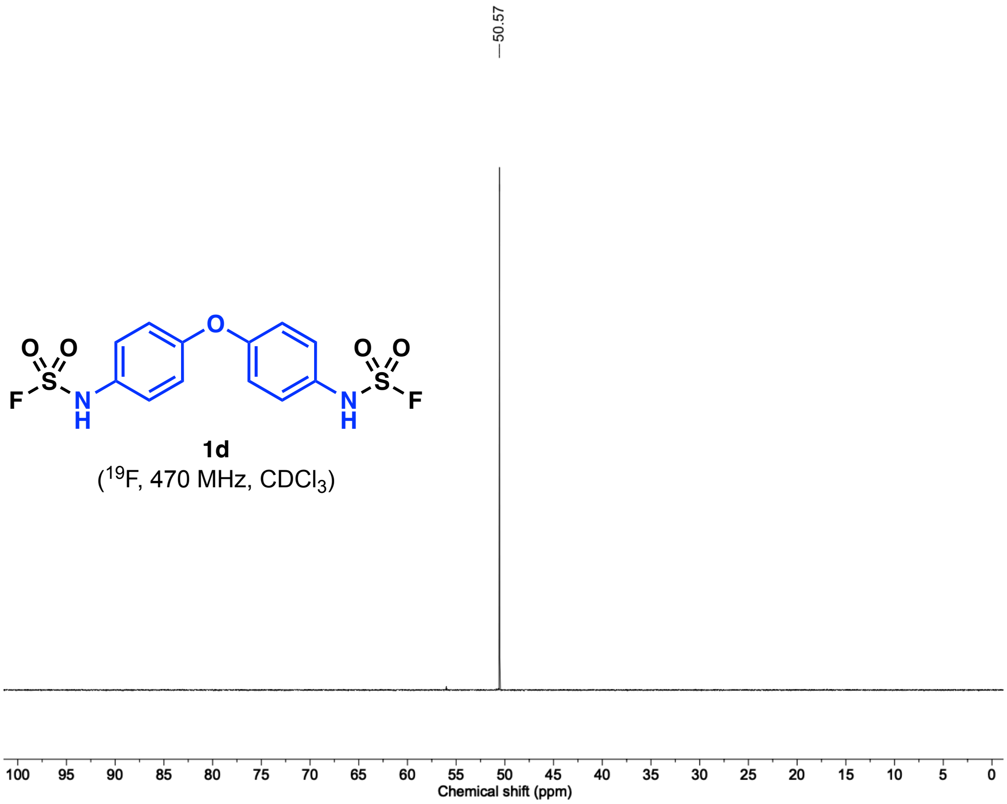
50.57
100 95 90 85 80 75 70 65 60 55 50 45 40 35 30 25 20 15 10 5 0
Chemical shift (ppm)
1d
(^{19}F, 470 MHz, $CDCl_3$)

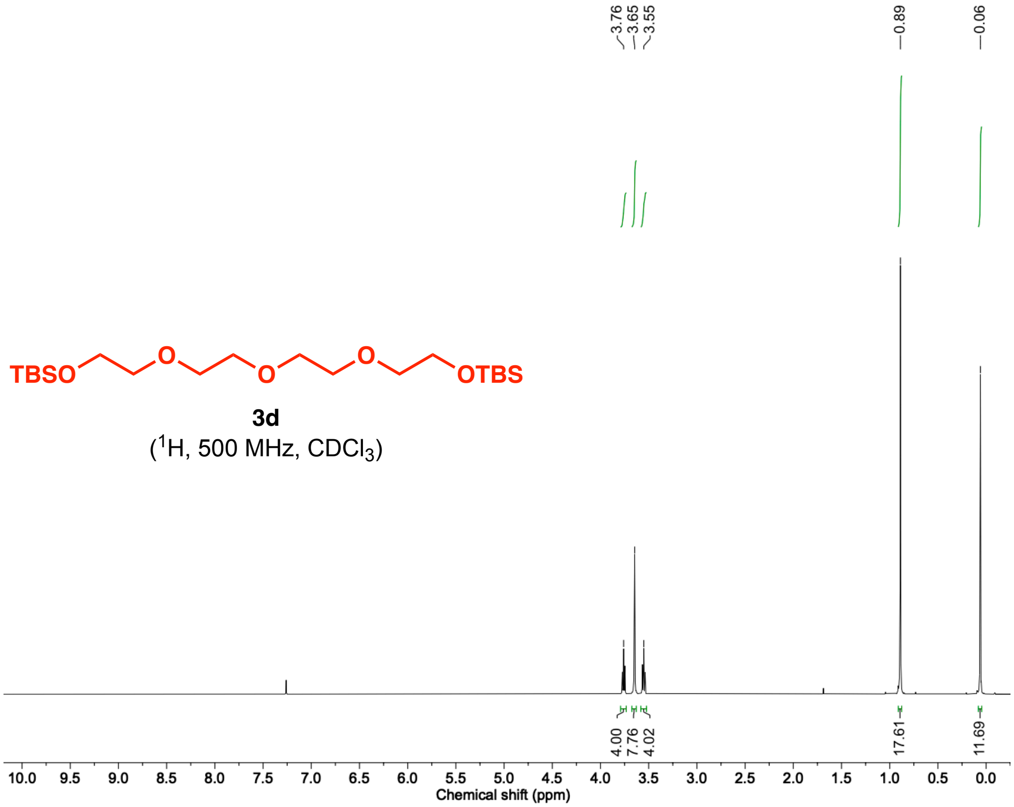

3d
(^{1}H, 500 MHz, $CDCl_3$)

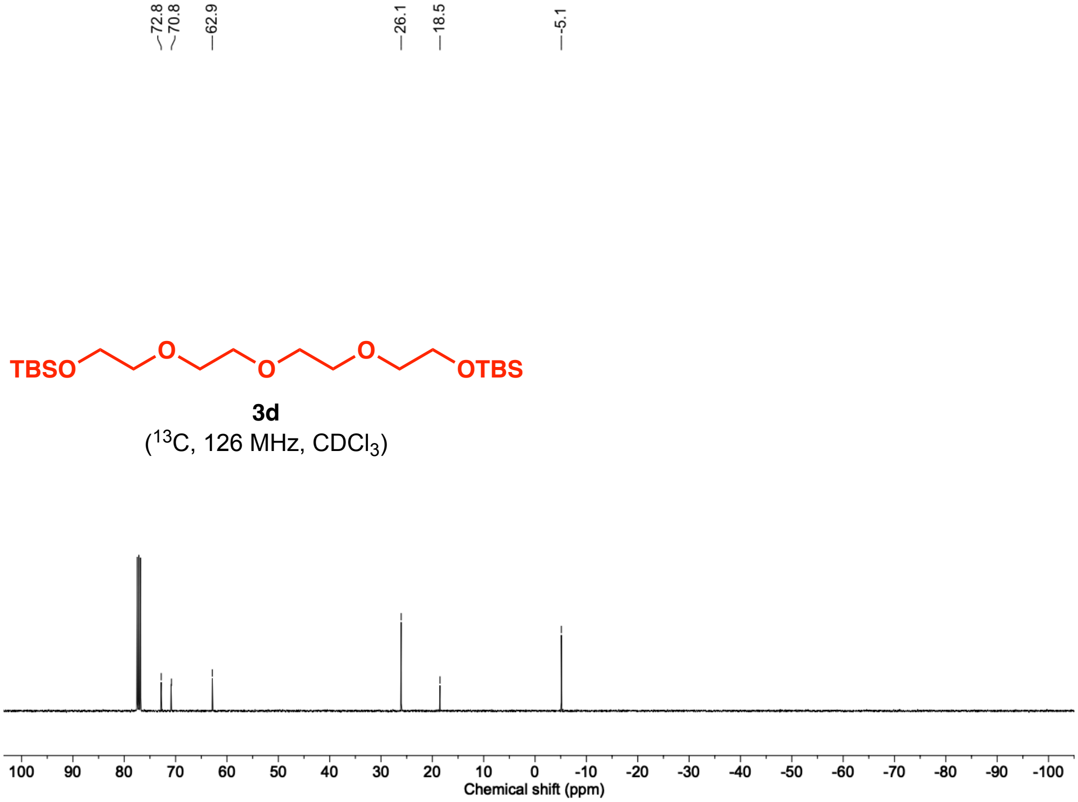

3d
(^{13}C, 126 MHz, $CDCl_3$)

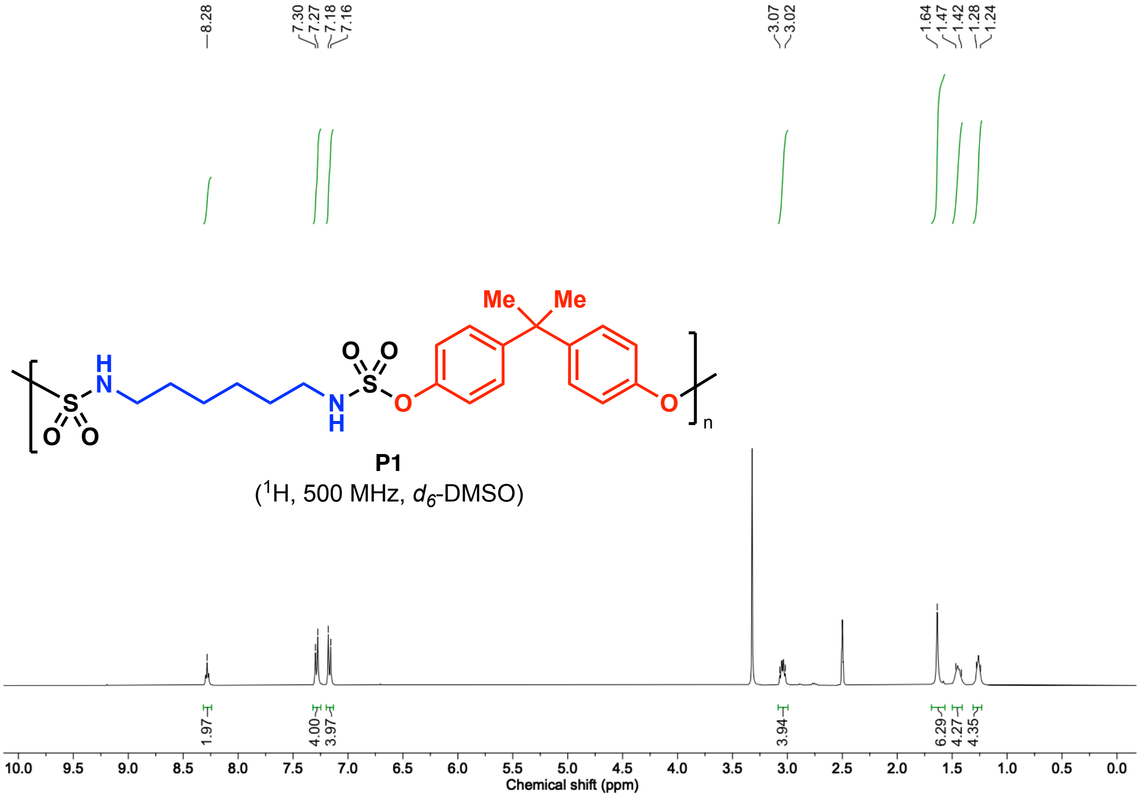

P1
(^{1}H, 500 MHz, d_6-DMSO)

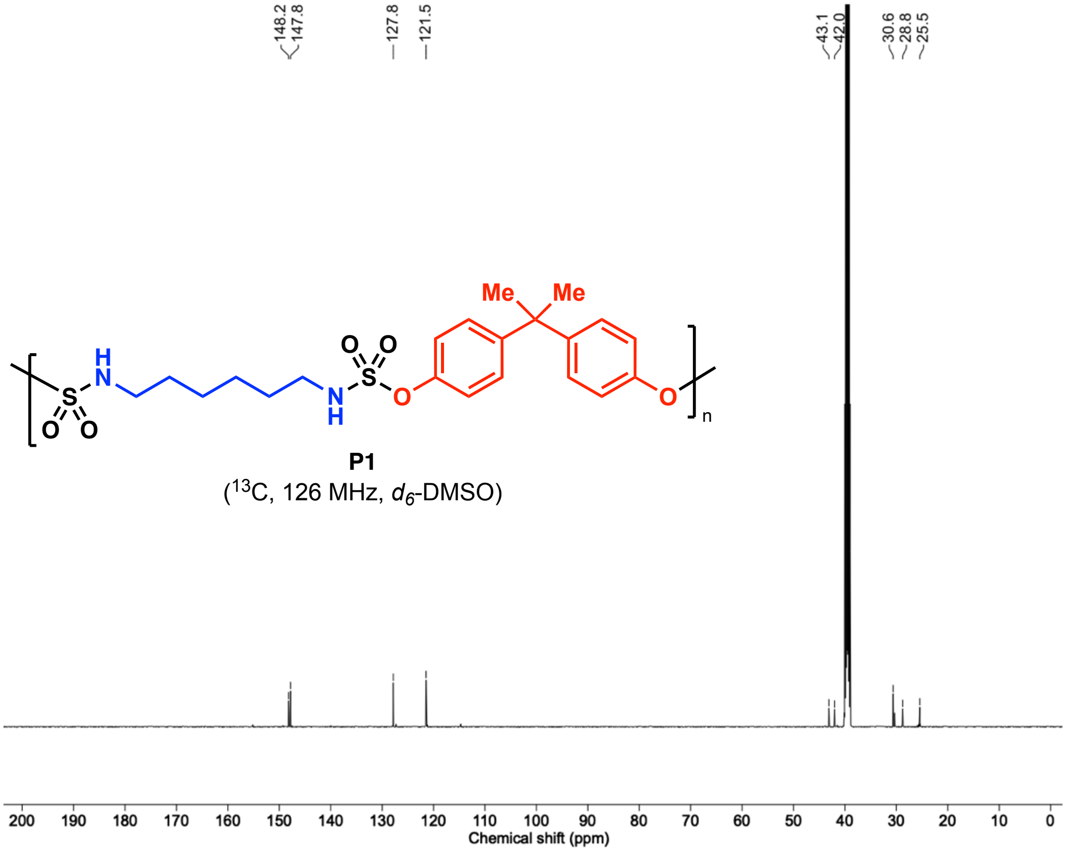

P1
(^{13}C, 126 MHz, d_6-DMSO)

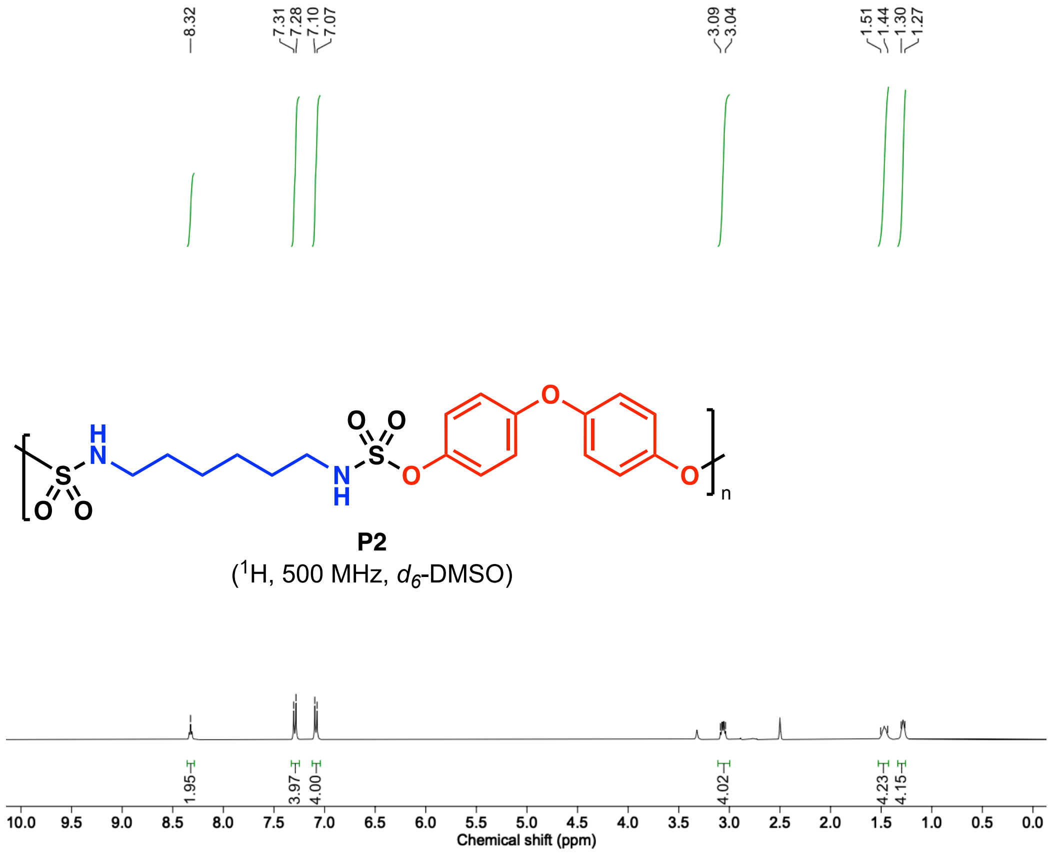
8.32
7.31
7.28
7.10
7.07
3.09
3.04
1.51
1.44
1.30
1.27
P2
(1H, 500 MHz, d6-DMSO)
1.95
3.97
4.00
4.02
4.23
4.15
Chemical shift (ppm)

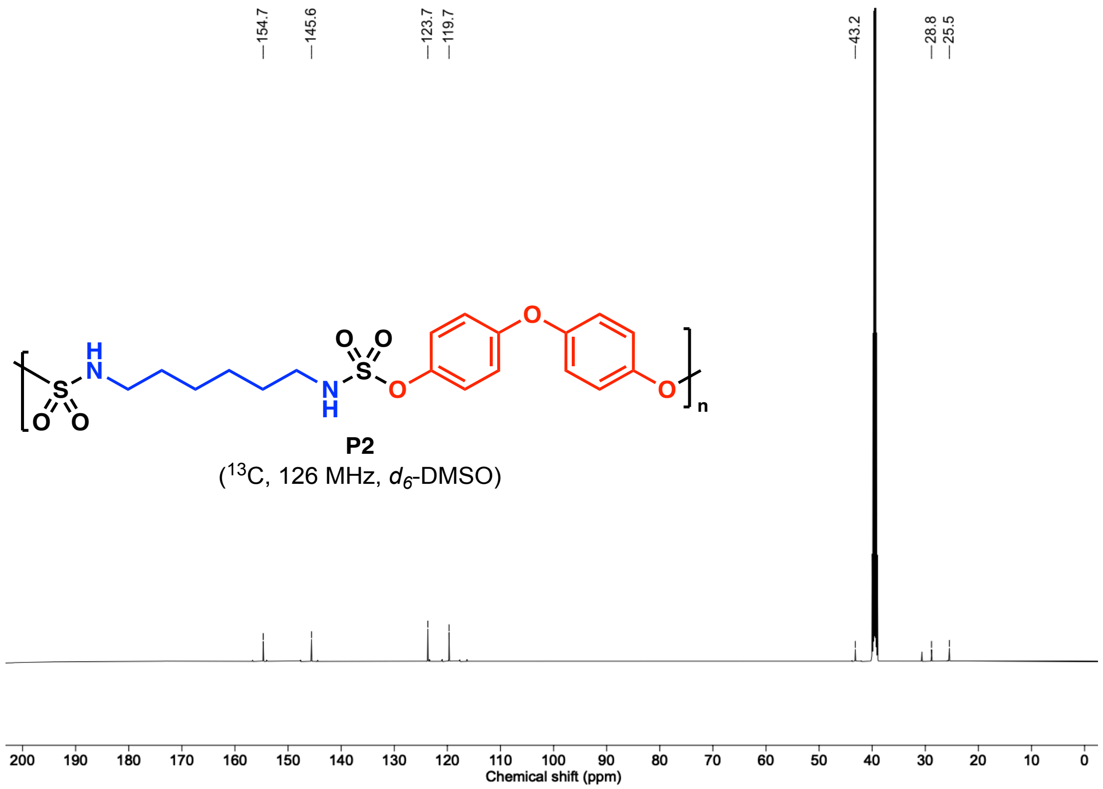
154.7
145.6
123.7
119.7
43.2
28.8
25.5
P2
(13C, 126 MHz, d6-DMSO)
Chemical shift (ppm)

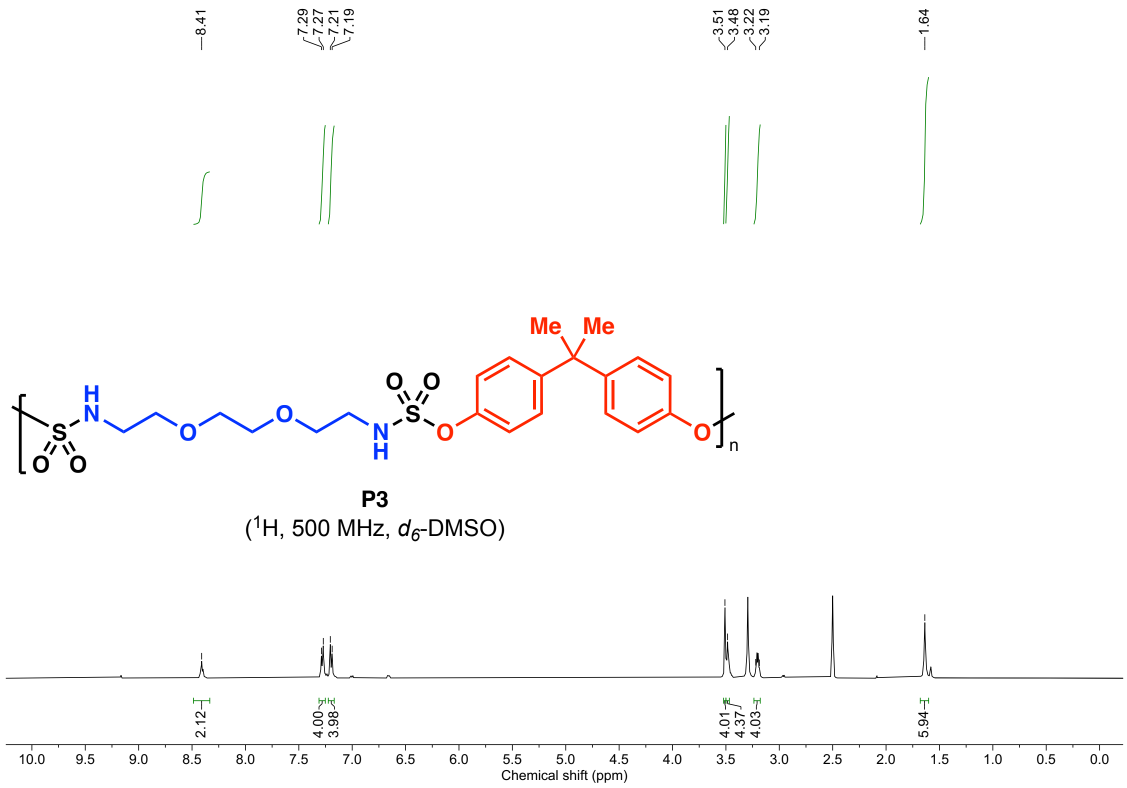

P3

(^{1}H, 500 MHz, d_6-DMSO)

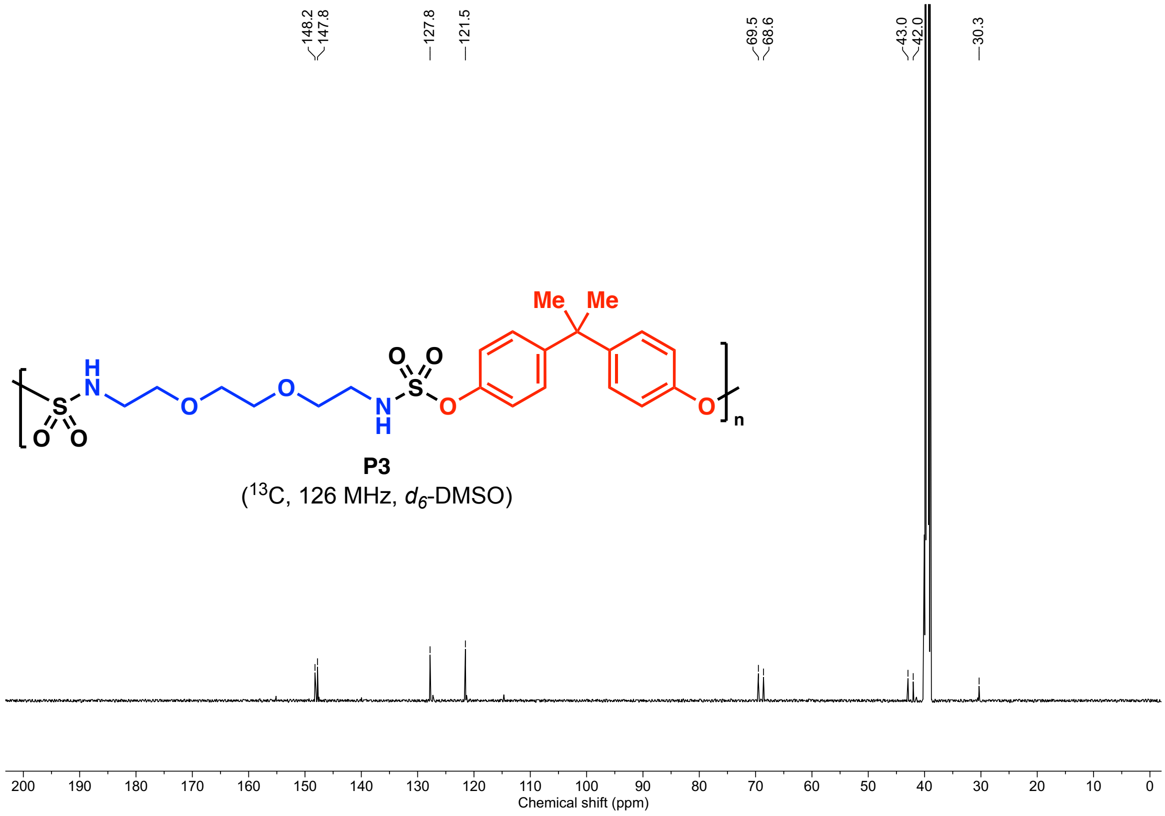

P3

(^{13}C, 126 MHz, d_6-DMSO)

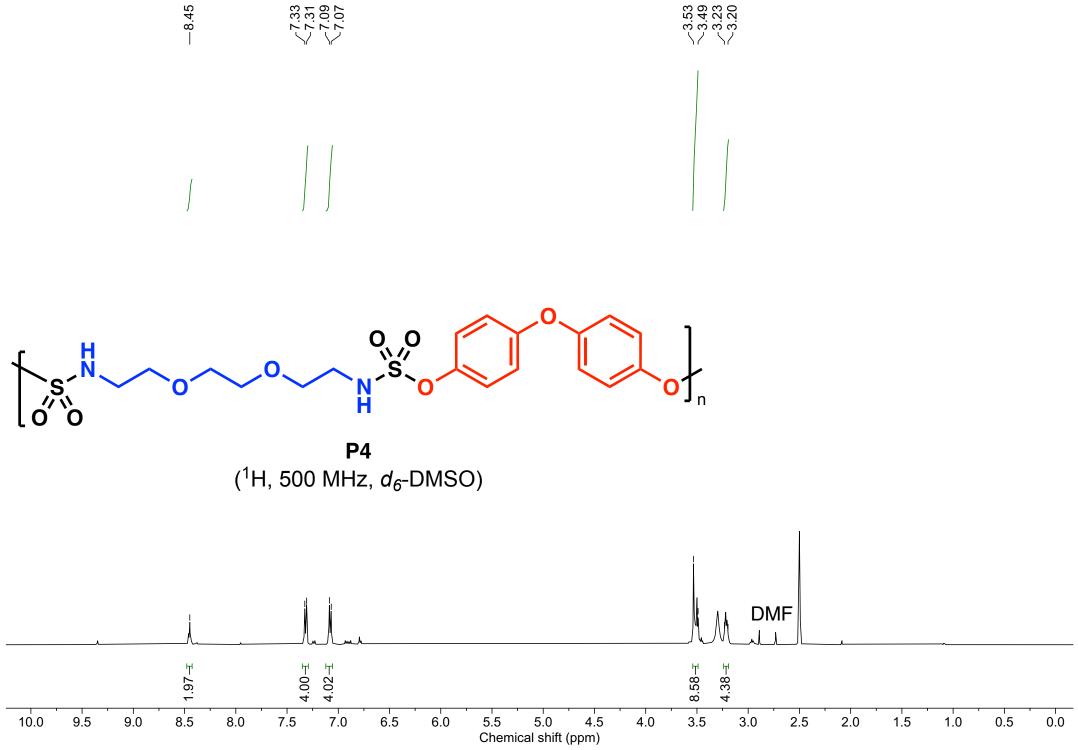

P4

(^{1}H, 500 MHz, d_6-DMSO)

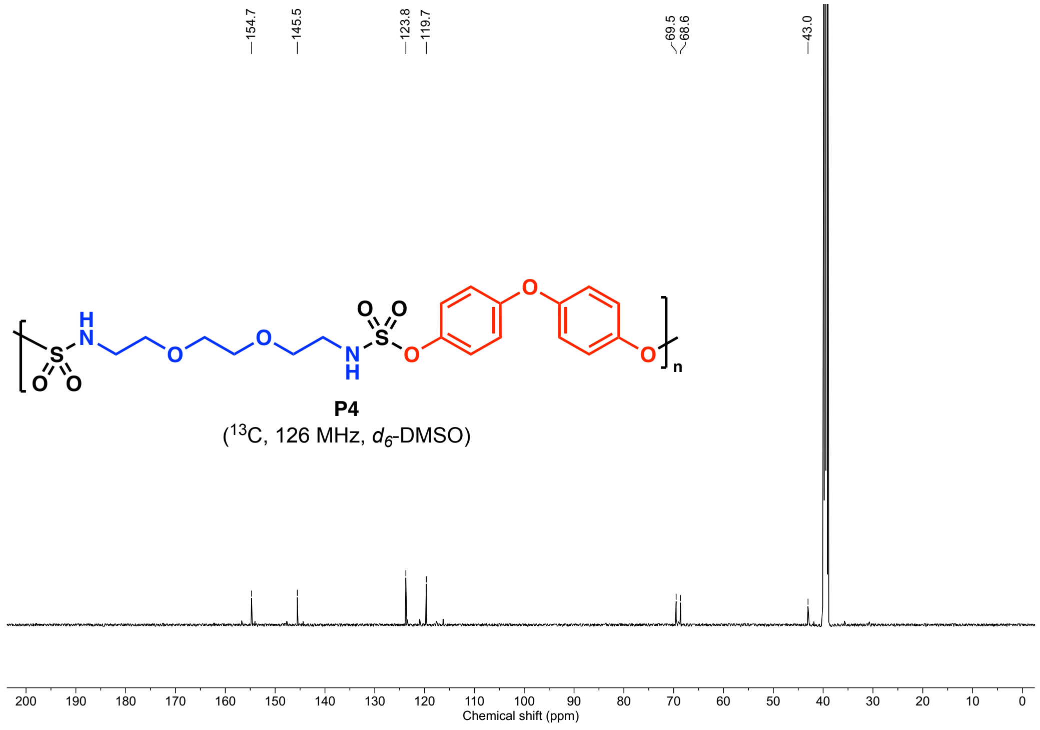

P4

(^{13}C, 126 MHz, d_6-DMSO)

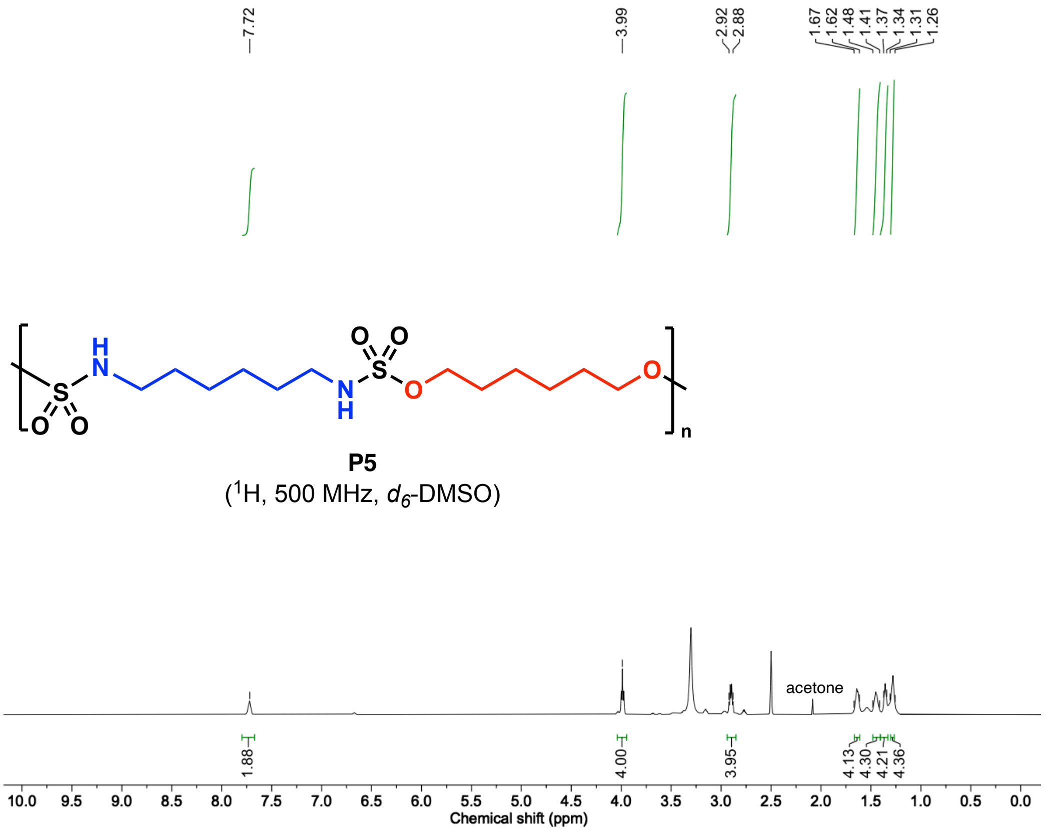

P5
(^{1}H, 500 MHz, d_6-DMSO)

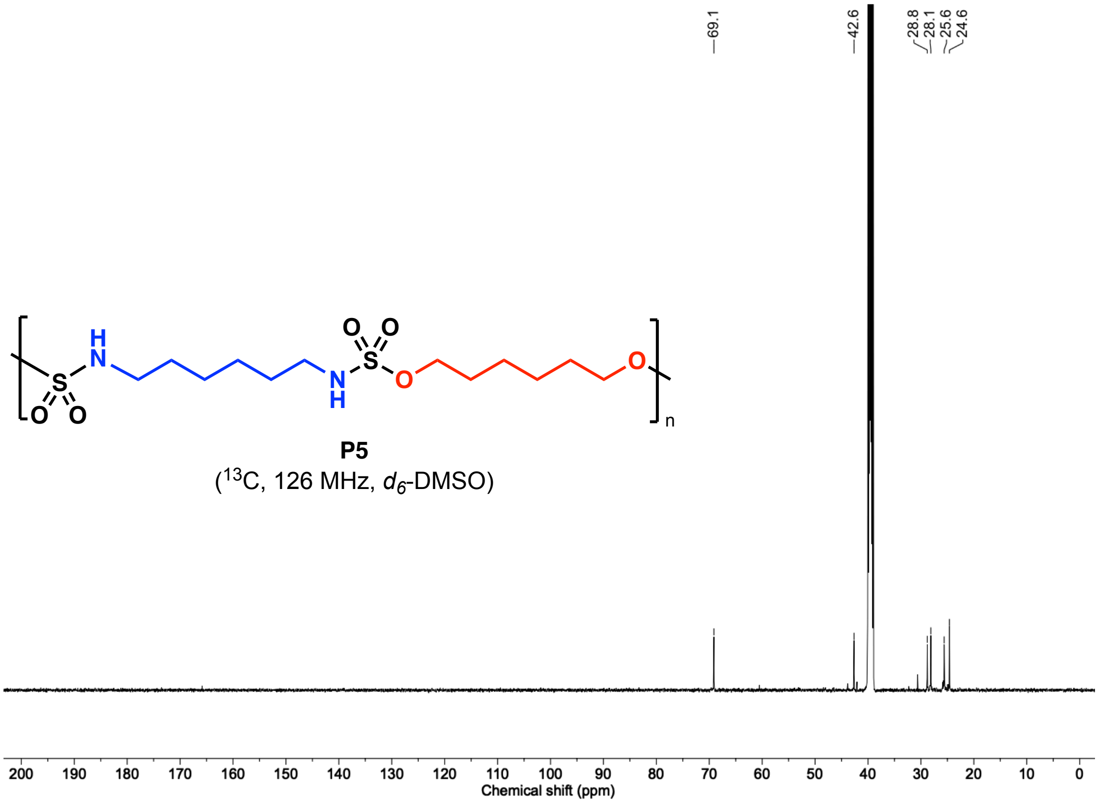

P5
(^{13}C, 126 MHz, d_6-DMSO)

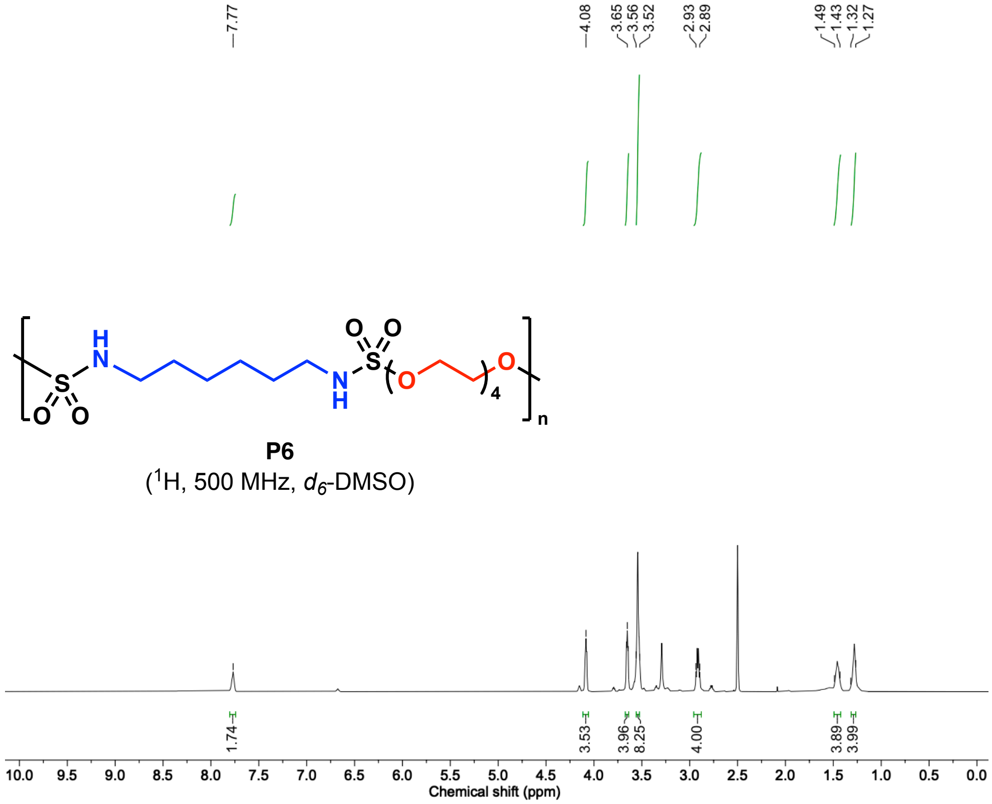
7.77
4.08
3.65
3.56
3.52
2.93
2.89
1.49
1.43
1.32
1.27
H
N
S
O
O
N
H
S
O
O
O
O
4
n
P6
(^{1}H, 500 MHz, d_6-DMSO)
1.74
3.53
3.96
8.25
4.00
3.89
3.99
10.0 9.5 9.0 8.5 8.0 7.5 7.0 6.5 6.0 5.5 5.0 4.5 4.0 3.5 3.0 2.5 2.0 1.5 1.0 0.5 0.0
Chemical shift (ppm)

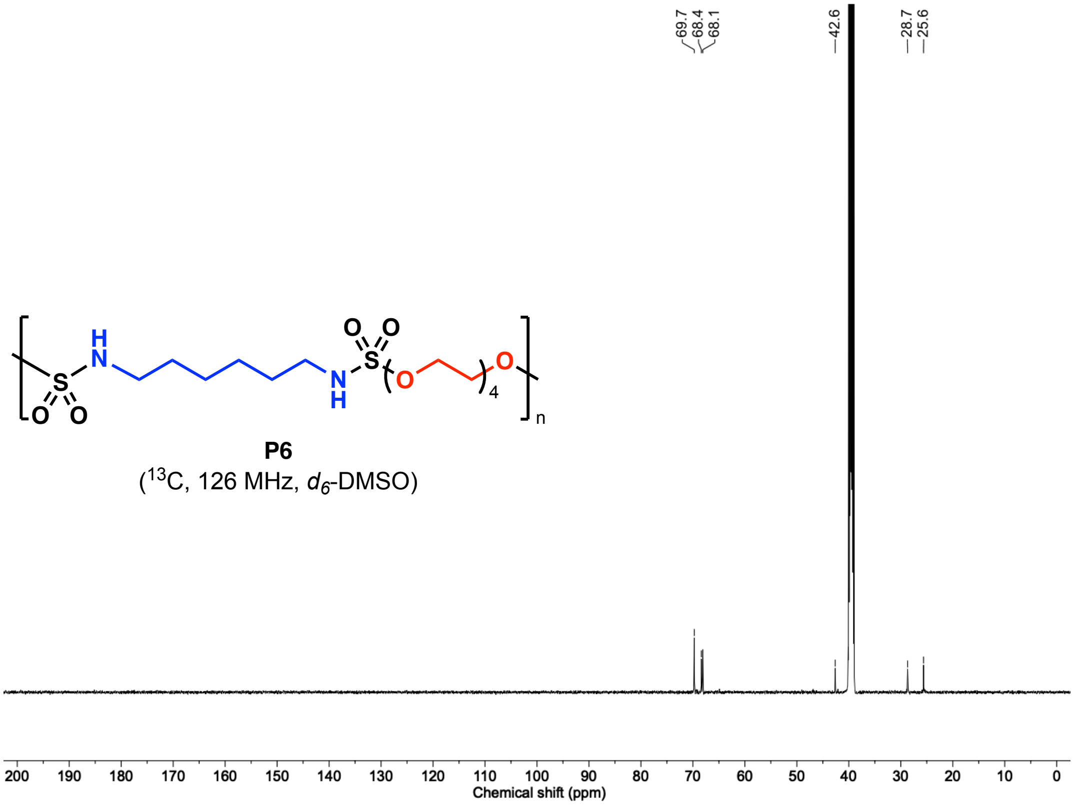
69.7
68.4
68.1
42.6
28.7
25.6
H
N
S
O
O
N
H
S
O
O
O
O
4
n
P6
(^{13}C, 126 MHz, d_6-DMSO)
200 190 180 170 160 150 140 130 120 110 100 90 80 70 60 50 40 30 20 10 0
Chemical shift (ppm)

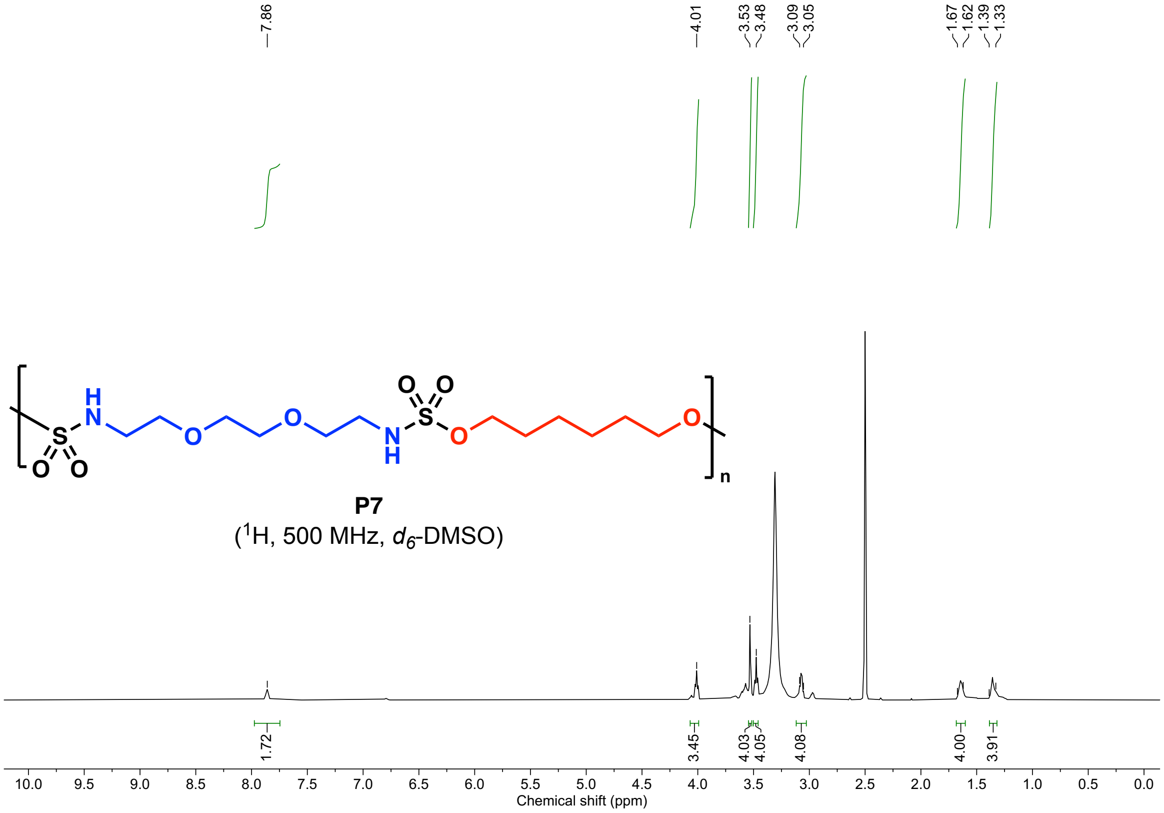
7.86
4.01
3.53
3.48
3.09
3.05
1.67
1.62
1.39
1.33
P7
(^{1}H, 500 MHz, d_6-DMSO)
1.72
3.45
4.03
4.05
4.08
4.00
3.91
Chemical shift (ppm)

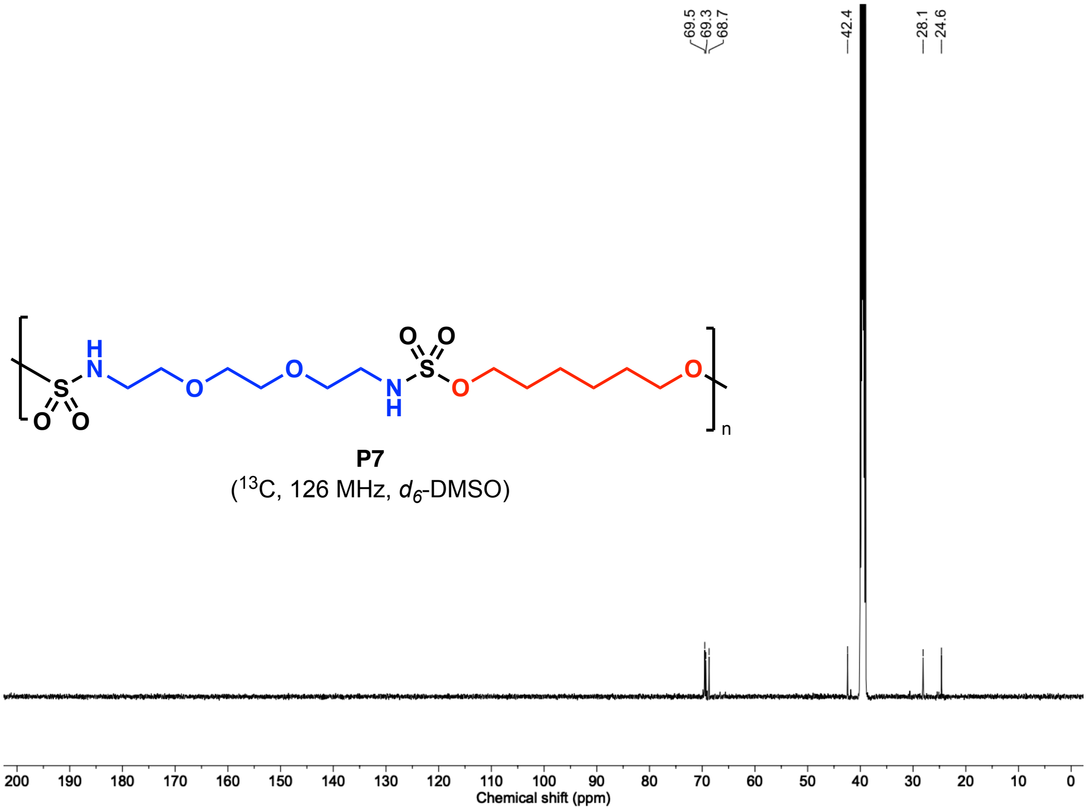
69.5
69.3
68.7
42.4
28.1
24.6
P7
(^{13}C, 126 MHz, d_6-DMSO)
Chemical shift (ppm)

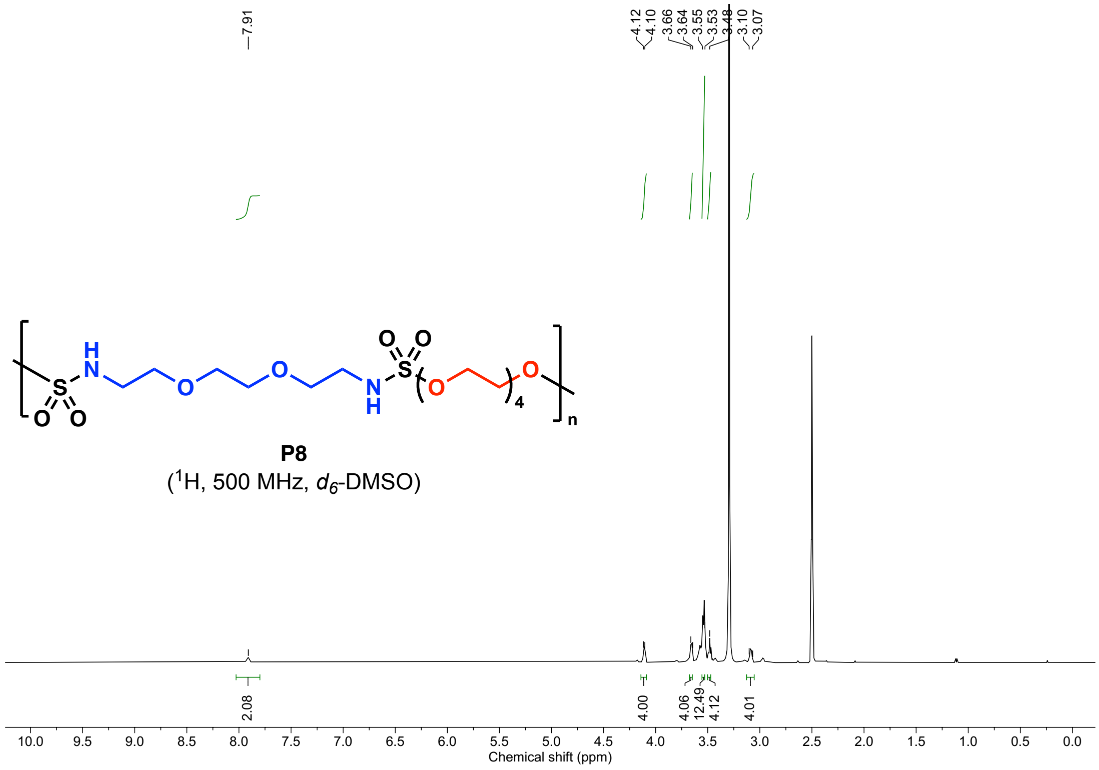

P8
(^{1}H, 500 MHz, d_6-DMSO)

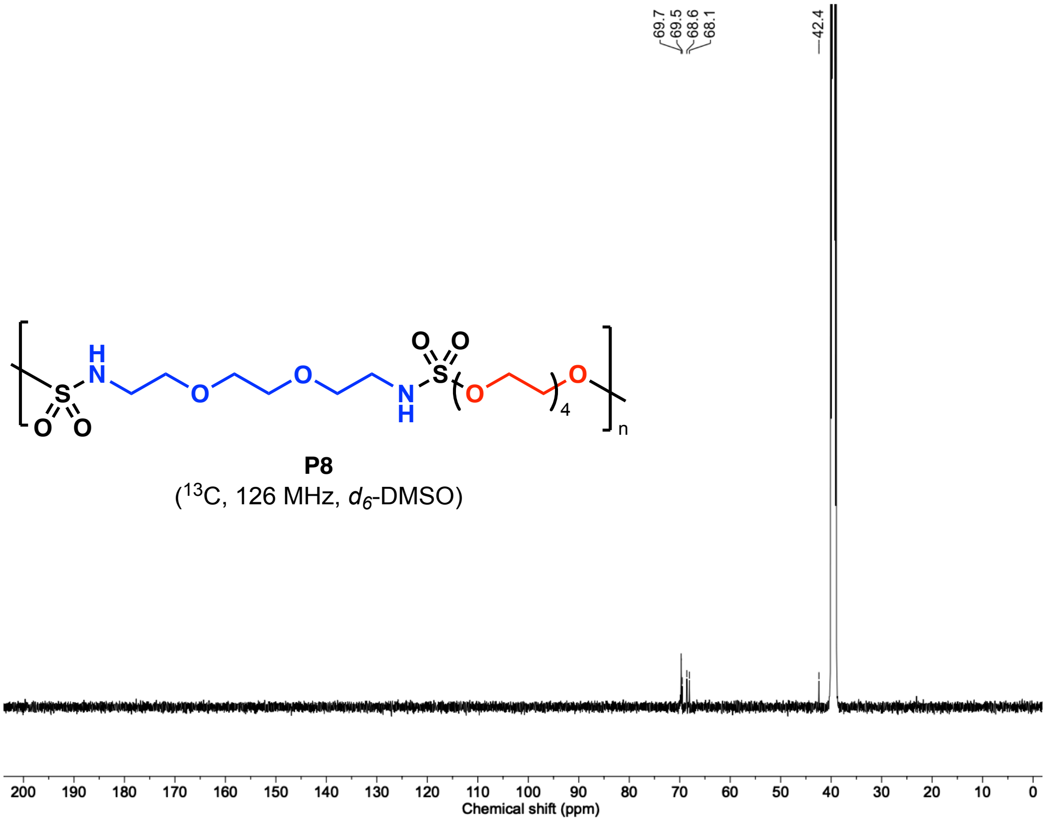

P8
(^{13}C, 126 MHz, d_6-DMSO)

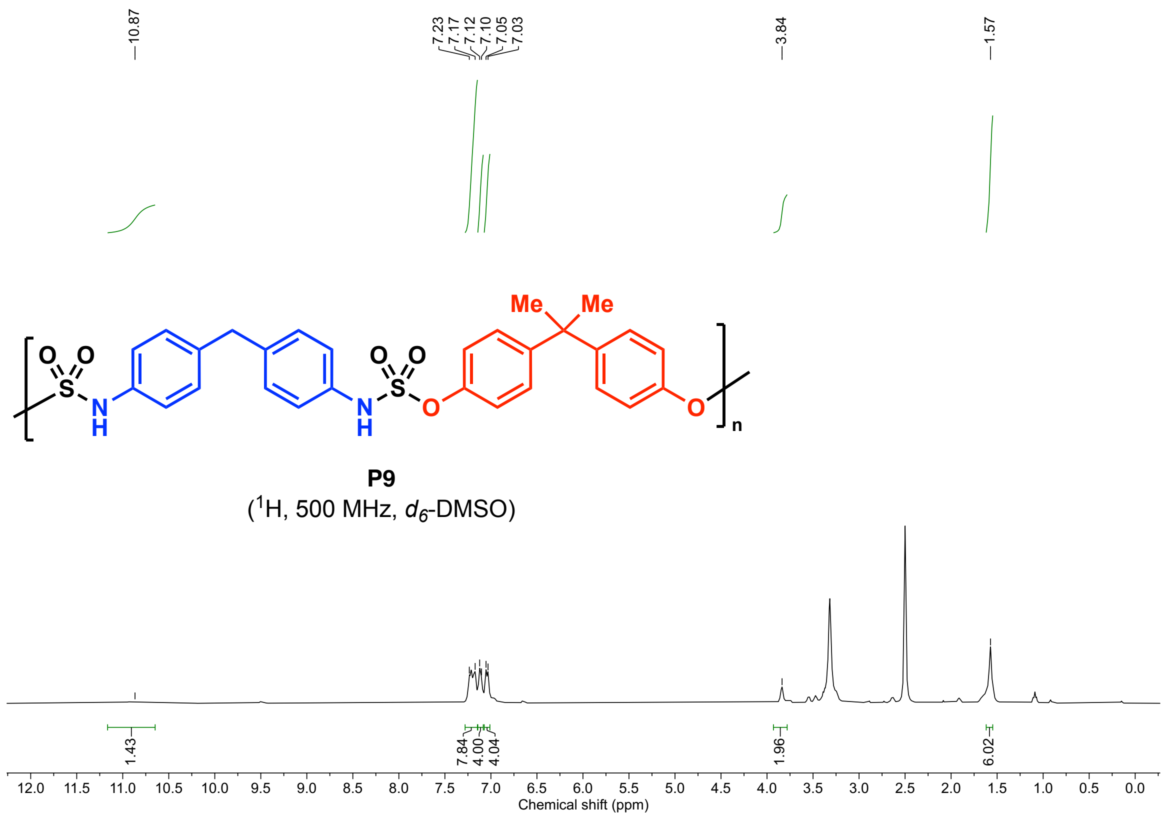

P9
(^{1}H, 500 MHz, d_6-DMSO)
Chemical shift (ppm)

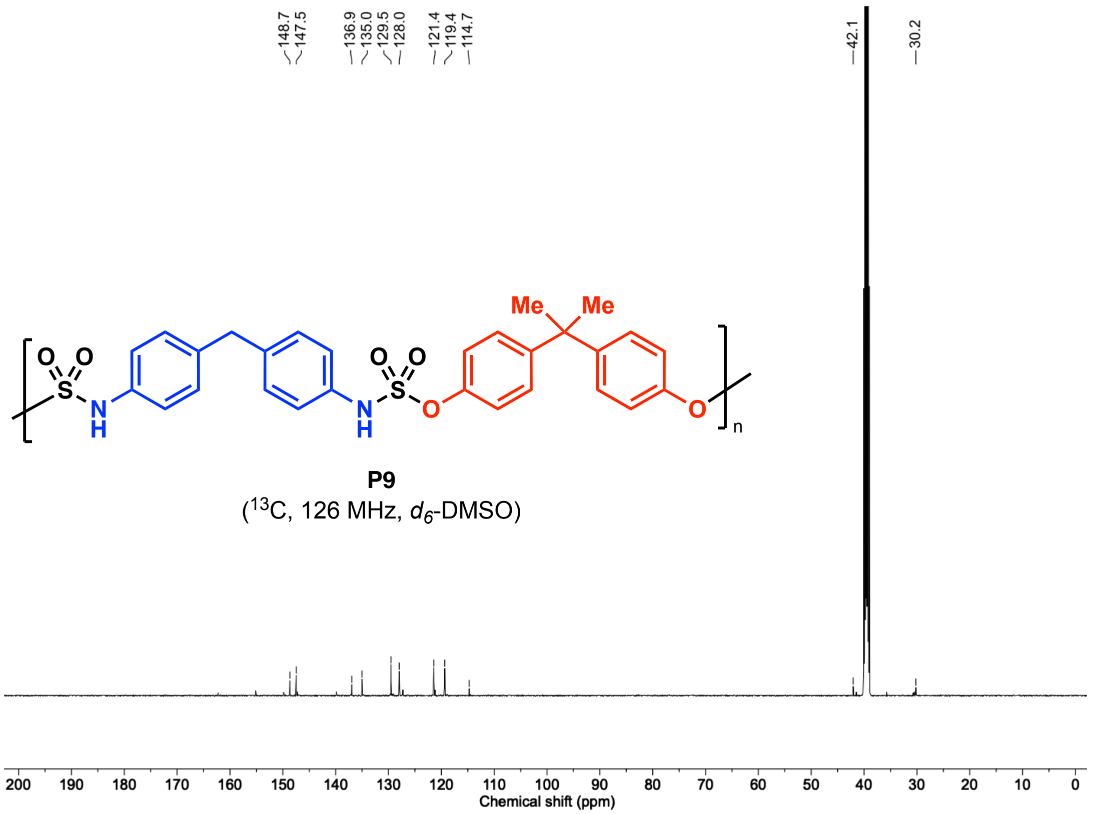

P9
(^{13}C, 126 MHz, d_6-DMSO)
Chemical shift (ppm)

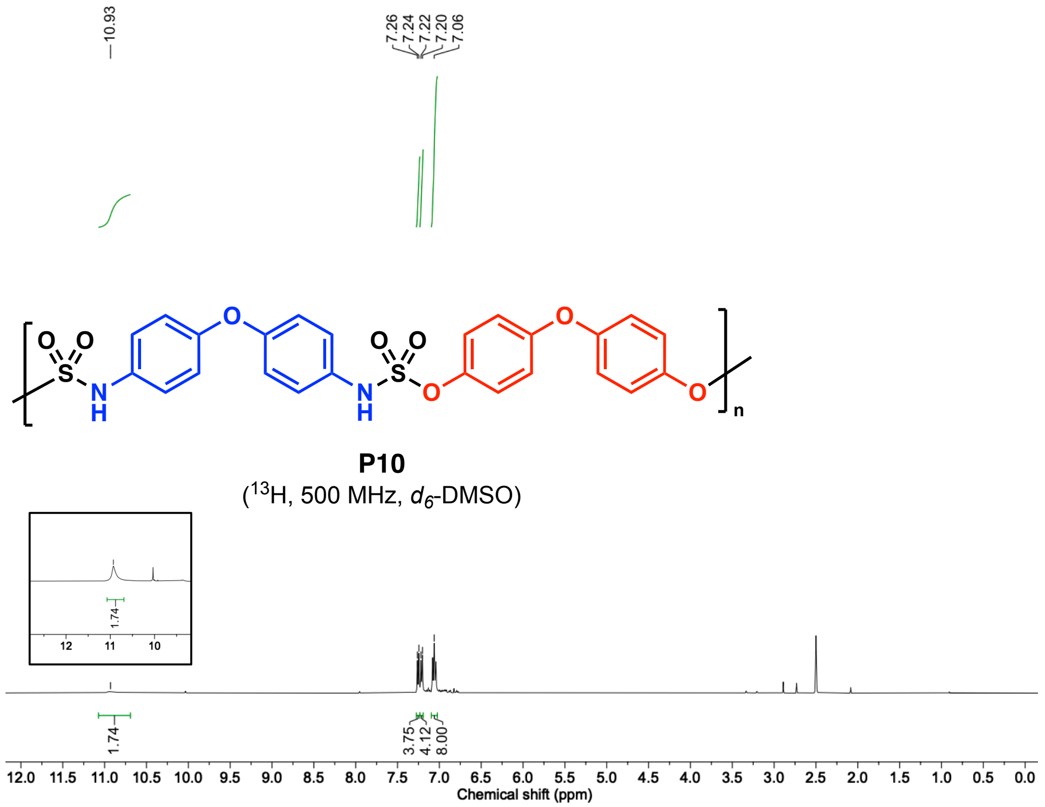

P10
(^{13}H, 500 MHz, d_6-DMSO)

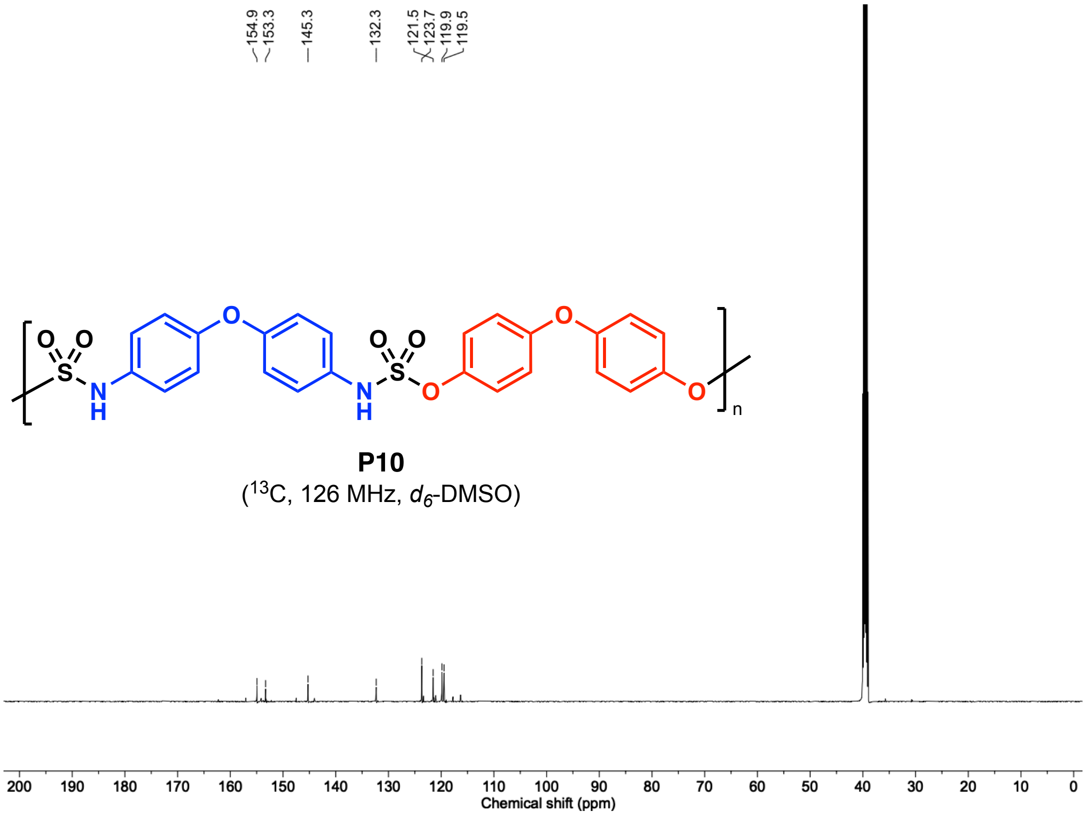

P10
(^{13}C, 126 MHz, d_6-DMSO)

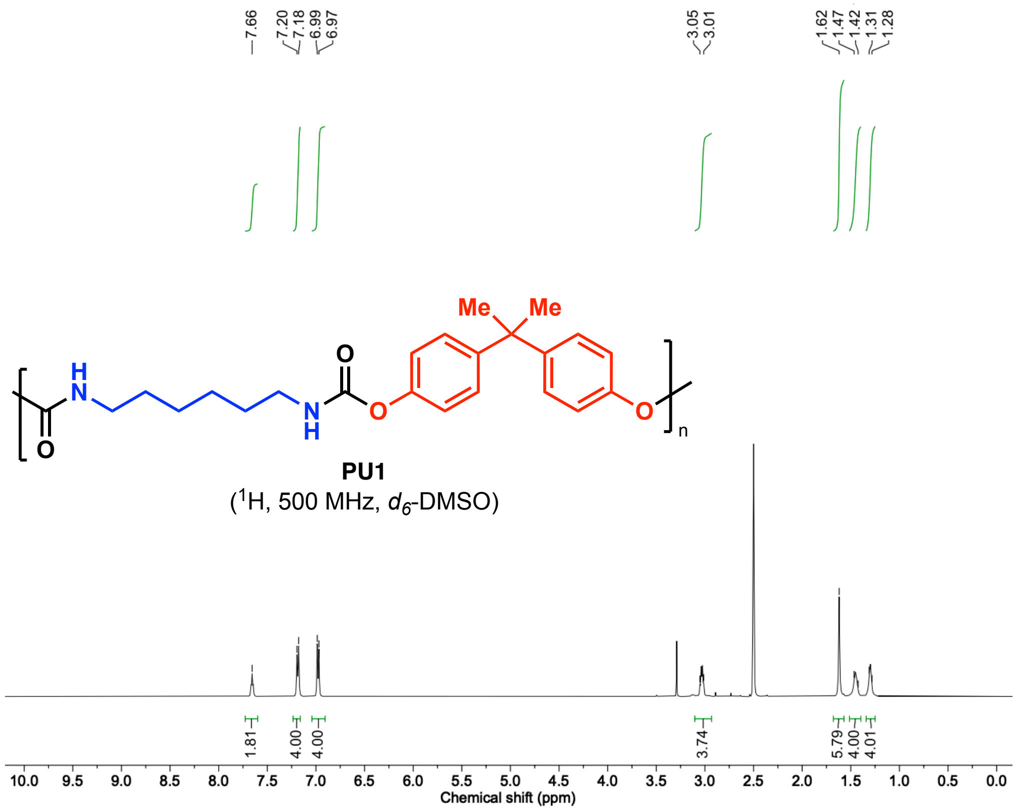
7.66
7.20
7.18
6.99
6.97
3.05
3.01
1.62
1.47
1.42
1.31
1.28
Me
Me
H
N
O
N
H
O
O
O
n
PU1
(1H, 500 MHz, d6-DMSO)
1.81
4.00
4.00
3.74
5.79
4.00
4.01
10.0 9.5 9.0 8.5 8.0 7.5 7.0 6.5 6.0 5.5 5.0 4.5 4.0 3.5 3.0 2.5 2.0 1.5 1.0 0.5 0.0
Chemical shift (ppm)

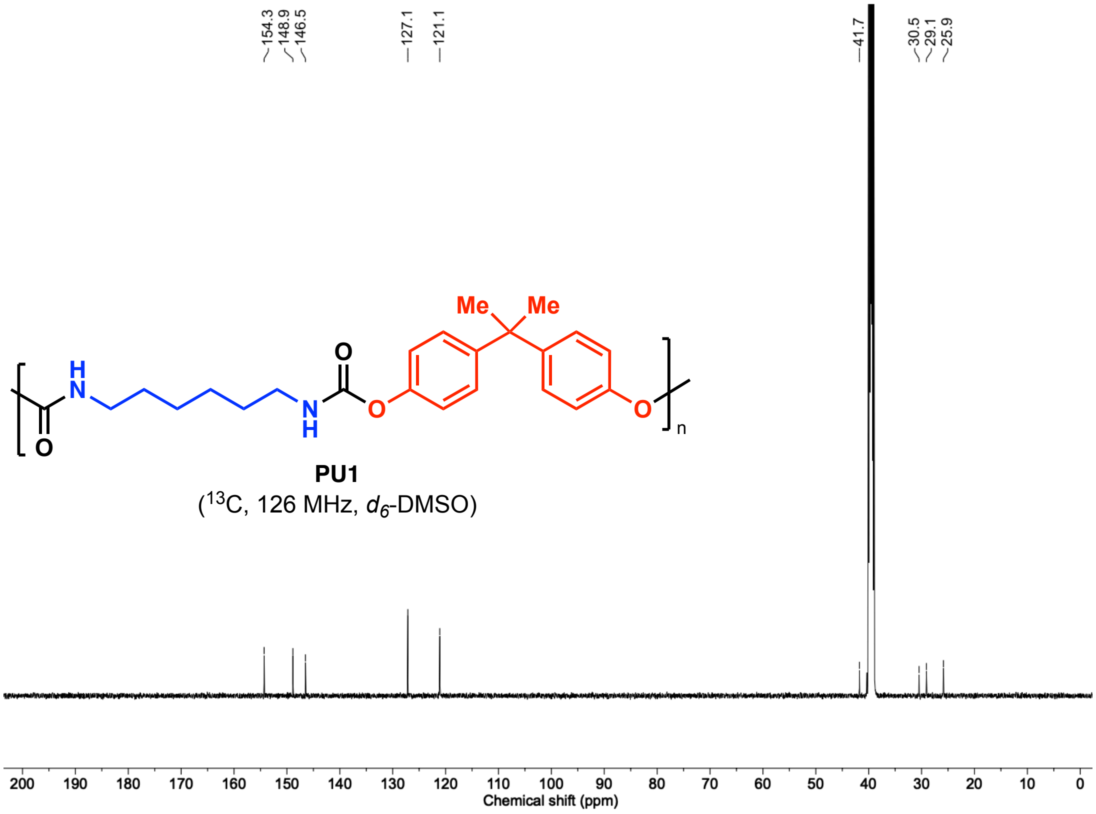
154.3
148.9
146.5
127.1
121.1
41.7
30.5
29.1
25.9
Me
Me
H
N
O
N
H
O
O
O
n
PU1
(13C, 126 MHz, d6-DMSO)
200 190 180 170 160 150 140 130 120 110 100 90 80 70 60 50 40 30 20 10 0
Chemical shift (ppm)

6. References:

(Following the numbering of the main text)

[36] R. W. Kulow, J. W. Wu, C. Kim, Q. Michaudel. Synthesis of unsymmetrical sulfamides and polysulfamides via SuFEx click chemistry. *Chem. Sci.* **2020**, *11*, 7807–7812.

[54] J. Dong, K. B. Sharpless, L. Kwisnek, J. S. Oakdale, V. V. Fokin. SuFEx-Based Synthesis of Polysulfates. *Angew. Chem. Int. Ed.* **2014**, *53*, 9466–9470.

[58] Z. Wu, J. W. Wu, Q. Michaudel, A. Jayaraman. Investigating the Hydrogen Bond-Induced Self-Assembly of Polysulfamides Using Molecular Simulations and Experiments. *Macromolecules* **2023**, *56*, 5033–5049.

[69] A. Basterretxea, Y. Haga, A. Sanchez-Sanchez, M. Isik, L. Irusta, M. Tanaka, K. Fukushima, H. Sardon. Biocompatibility and hemocompatibility evaluation of polyether urethanes synthesized using DBU organocatalyst. *Eur. Polym. J.* **2016**, *84*, 750–758.

[70] W. C. Oliver, G. M. Pharr. An improved technique for determining hardness and elastic modulus using load and displacement sensing indentation experiments. *J. Mat. Res.* **1992**, *7*, 1564–1583.

[72] A. Abri, S. Ranjdar. Preparation of Nano Silica Supported Sodium Hydrogen Sulfate: As an Efficient Catalyst for the Trimethyl, Triethyl and t-Butyldimethyl Silylations of Aliphatic and Aromatic Alcohols in Solution and under Solvent-free Conditions. *J. Chin. Chem.Soc.* **014**, *61*, 929–934.
